# Design and Synthesis of Novel Betulin Derivatives Containing Thio-/Semicarbazone Moieties as Apoptotic Inducers through Mitochindria-Related Pathways

**DOI:** 10.3390/molecules26216356

**Published:** 2021-10-21

**Authors:** Jiafeng Wang, Jiale Wu, Yinglong Han, Jie Zhang, Yu Lin, Haijun Wang, Jing Wang, Jicheng Liu, Ming Bu

**Affiliations:** 1 College of Pharmacy, Qiqihar Medical University, Qiqihar 161006, China; wangjiafeng410323@163.com (J.W.); wwwwjl55@163.com (J.W.); han13836240549@163.com (Y.H.); zhangjie_qmu@163.com (J.Z.); linyu7373@163.com (Y.L.); wanghaijun@qmu.edu.cn (H.W.); qyyx305@163.com (J.W.); 2 Research Institute of Medicine & Pharmacy, Qiqihar Medical University, Qiqihar 161006, China; qyyliu@163.com

**Keywords:** betulin derivatives, thiosemicarbazone, semicarbazone, antitumor, apoptosis

## Abstract

Two new series of betulin derivatives with semicarbazone (**7a**–**g**) or thiosemicarbazone (**8a**–**g**) groups at the C-28 position were synthesized. All compounds were evaluated for their in vitro cytotoxicities in human hepatocellular carcinoma cells (HepG2), human breast carcinoma cells (MCF-7), human lung carcinoma cells (A549), human colorectal cells (HCT-116) and normal human gastric epithelial cells (GES-1). Among these compounds, **8f** displayed the most potent cytotoxicity with an IC_50_ value of 5.86 ± 0.61 μM against MCF-7 cells. Furthermore, the preliminary mechanism studies in MCF-7 cells showed that compound **8f** could trigger the intracellular mitochondrial-mediated apoptosis pathway by losing MMP level, which was related with the upregulation of Bax, P53 and cytochrome c expression; the downregulation of Bcl-2 expression; activation of the expression levels of caspase-3, caspase-9, cleaved caspase-3 and cleaved caspase-9; and an increase in the amounts of intracellular reactive oxygen species. These results indicated that compound **8f** may be used as a valuable skeleton structure for developing novel antitumor agents.

## 1. Introduction

**Figure 1:**
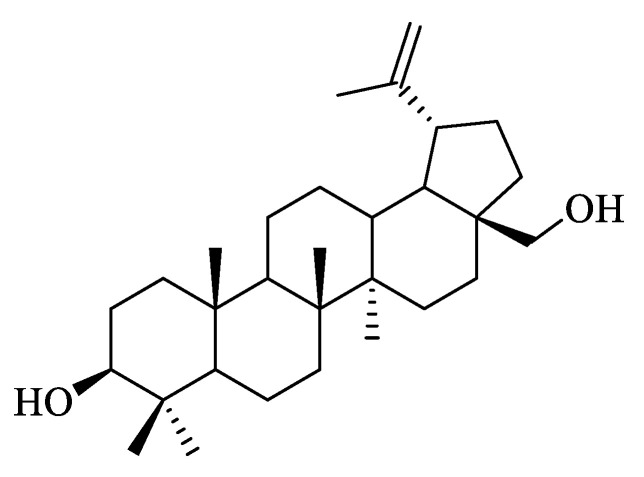
Structure of betulin (BE, **1**).

Nowadays, cancer has become the second leading cause of human death worldwide [1]. The most effective therapies used in cancer treatment continue to be traditional cytotoxic agents [2]. It is worth noting that the exploitation of high-effect and low-toxicity anticancer drugs based on natural products is still one of the effective directions in the field of drug research [3,4,5]. Betulin (BE, lup-20(29)-ene-3β,28-diol, **1**) is an important natural lupine-type triterpenoid widely distributed in plenty of plants, especially abundant in the bark of birch trees (Figure 1) [6]. Betulin has been shown to exert various biological and pharmacological activities, such as antibacterial [7,8], anti-HIV [9,10,11] and anti-inflammatory properties [12,13,14,15]. Recently, plenty of studies have reported that betulin and its derivatives have significant antitumor activities against many kinds of cancer cell lines, such as colorectal carcinoma (HT29, HCT116) [16,17], lung carcinoma (A549) [18], liver carcinoma (SK-HEP-1, HepG2) [19,20], breast carcinoma (MCF-7, MDA-MB231) [21], prostate carcinoma (PC3) [22] as well as cervical carcinoma (HeLa) [23] and leukemia (HL-60, K562, U937) [24,25,26]. However, the high hydrophobicity of betulin hampers its further development as a cytotoxic drug [27,28,29].

**Figure 2:**
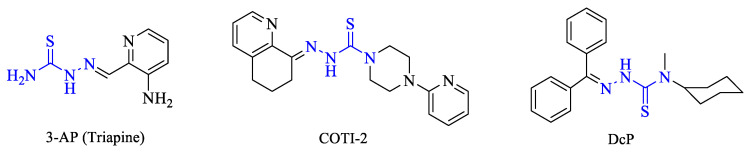
Representative compounds containing thiosemicarbazone groups.

Thiosemicarbazones and semicarbazones are classes of Schiff bases, which are highly regarded by medicinal chemists due to their extensive pharmacological properties or bioactivities, such as antifungal [30], anti-chlamydial [31], antibacterial [32,33,34] and antimalarial activities [35,36,37,38]. Notably, thio-/semicarbazone compounds have a wide antitumor spectrum against various tumor types, such as breast cancer [39,40,41], non-small-cell lung cancer [42], pancreatic cancer [43], leukemia [44], bladder cancer [45] and prostate cancer [46]. Recently, several representative compounds containing thio-/semicarbazone groups in the clinical trial have been reported, such as DcP [47], 3-AP [48] and COTI-2 [49]. 3-AP was initially designed as a potent ribonucleotide reductase (RR) inhibitor for cancer treatment and has since entered Phase II clinical trials [50]. Representative compounds containing thiosemicarbazone groups are shown in Figure 2.

On the one hand, our previous work mainly focused on the structural modifications of natural compounds [51,52,53]. We successfully synthesized a series of endoperoxide steroidal derivatives containing semicarbazone or thiosemicarbazone groups and obtained several new structures with significant antitumor activity [54]. On the other hand, there are three independent active positions in betulin, namely, the isopropenyl side chain at C-19 and two hydroxyl groups at C-3 and C-28. It is quite feasible to make a chemical modification of different sites to obtain novel betulin derivatives with desired biological properties. According to the structure–activity relationship of betulin, introducing a hydrogen donor group at the C-28 position may improve cytotoxic activity significantly.

**Figure 3:**
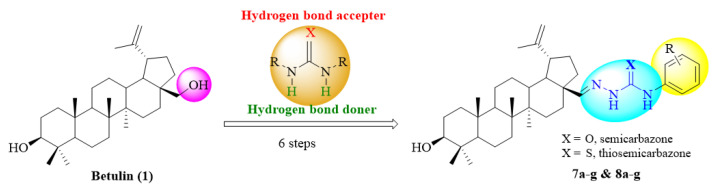
Design strategy of novel betulin derivatives.

Thus, inspired by the good biological property of thio-/semicarbazones, in view of the potential medicinal research value of betulin and in continuation of an ongoing program aiming at developing more potential anticancer drugs, in the present study, 14 betulin derivatives modified at the C-28 position with thio-/semicarbazide groups were designed and synthesized (Figure 3). We hope to obtain valuable information about the impact of thio/semicarbazone moiety at the C-28 position on cytotoxic activity and their underlying mechanisms of antitumor effect.

## 2. Results and Discussion

### 2.1. Synthesis of Botulin Derivatives

**Scheme 1:**
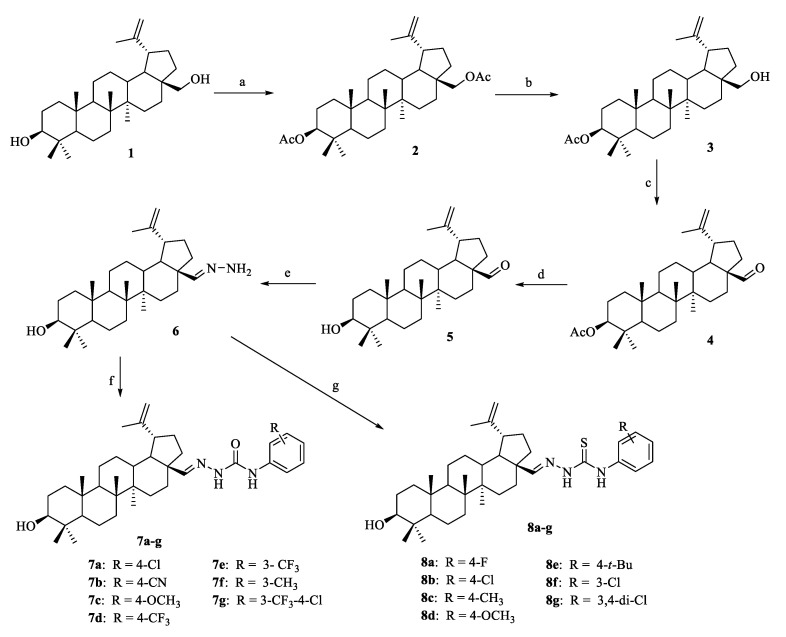
Synthetic pathway to compounds **7a**–**g** and **8a**–**g**. Reagents and conditions: (**a**) Ac_2_O, DMAP, pyridine, room temperature, 6 h, 80%; (**b**) Ti(*i*-PrOH)_4_, *i*-PrOH, 85 °C, 5 h, 76%; (**c**) PCC, CH_2_Cl_2_, 35 °C, 1 h, 80%; (**d**) NaOH, CH_3_OH, 80 °C, 2 h, 65%; (**e**) NH_2_NH_2_-H_2_O, CH_3_CH_2_OH, 40 °C, 5 h, 70%; (**f**) CH_3_CH_2_OH, AcOH, phenyl isocyanates for **7a**–**g**, room temperature, 2~5 h, 75%~88%; (**g**) CH_3_CH_2_OH, AcOH, phenyl thioisocyanates for **8a**–**g**, room temperature, 2~5 h, 72%~87%.

The general synthesis route of betulin derivatives is shown in Scheme 1. Two hidroxyls of betulin (**1**) were acetylated with acetic anhydride in dry pyridine to synthesis compound **2**. Compound **2** further reacted with Ti(*i*-PrOH)_4_ in dry isopropyl alcohol for selective deacetylation at C-28 to give compound **3**. Then, the 28-OH of betulin was oxidized to a carbonyl group in the presence of pyridinium chlorochromate in dry dichloromethane to give compound **4**. Subsequently, compound **4** reacted with sodium hydroxide for deacetylation at C-3 to give compound **5**. Compound **5** was further reacted with hydrazine hydrate in ethanol to get hydrazine **6**. At last, hydrazine **6** reacted with phenyl isothiocyanate or isocyanate substituents in the presence of acetic acid in ethanol to obtain target novel betulin-(thio-)semicarbazone derivatives **7a**–**g** and **8a**–**g.** The chemical structures of all new synthesized compounds were characterized by HRMS and NMR spectrum methods (Supplementary Materials).

### 2.2. Biological Evaluation of Betulin Derivatives

#### 2.2.1. Betulin Derivatives Inhibited Proliferation of Various Human Cancer Cells

The in vitro cytotoxicities of all betulin derivatives **7a**–**g** and **8a**–**g** were evaluated using MTT assays against human hepatocellular carcinoma cells (HepG2), human breast carcinoma cells (MCF-7), human lung carcinoma cells (A549), human colorectal cells (HCT-116) and normal gastric cells (GES-1). Mitomycin C was tested as a positive drug control. The cytotoxicities of all compounds are summarized as IC_50_ values in Table 1. The results showed that most of compounds have remarkable cytotoxicities toward all four tested human tumor cell lines and were more potent than betulin parent.

For the HepG2 cell line, the semicarbazone series of compounds **7a**, **7b**, **7d**, **7e** and **7g** (X = O) displayed lower cytotoxic activities than betulin (IC_50_ = 20.42 μM). The substitutions (methyl, methoxy) on the semicarbazone moiety led to compounds **7c** and **7f** without improvement in cytotocxicity. Compound **7e** possessing 3-trifluoromethyl group displayed significant cytotoxic activity with IC_50_ value of 8.93 μM. It is about 2.28-fold higher than botulin. The thiosemicarbazone series of compounds **8a**, **8b**, **8f** and **8g** also displayed lower cytotoxic activities than betulin. For compound **8f** possessing 3-chloro group (IC_50_ = 6.87 μM), the cytotoxicity was 2.97-fold higher than betulin. Furthermore, compounds **7e** and **8f** were less toxic to GES-1 cells with IC_50_ values of 196.18 and 214.60 μM, respectively. The data showed that the incorporation of a 3-chloro or 3-trifluoromethyl group with semicarbazone or thiosemicarbazone moieties at the C28 of betulin led to significant improvement in cytotoxic activity than tertiary butyl or methyl. 

For MCF-7 cell line, compounds **7b**, **7e**, **7g**, **8a**, **8b** and **8f** also possessed stronger cytotoxicity than that of betulin, and all IC_50_ values were lower than 10 μM. Among them, compound **8f** (IC_50_ = 5.86 μM) was the most active one, which was 3.32-fold more potent than betulin. Moreover, compound **8f** (IC_50_ = 214.60 μM) showed lower cytotoxicity against GES-1 cells. The compound **7e** (IC_50_ = 5.96 μM) was also 3.10-fold more than betulin. The results suggested that the electron-donating substitution with semicarbazone or thiosemicarbazone moiety at the C28 of betulin was beneficial for compounds to displayed remarkable cytotoxicity against MCF-7 cells.

Taken together, the two series of betulin derivatives generally exhibited potent cytotoxicity compared with betulin against MCF-7 and HepG2 cell lines. The results suggest that both the conjugated N,N,S- and N,N,O-tridentate donor sets are essential for the cytotoxicities of the novel betulin derivatives.

Among these compounds under biological study, the thiosemicarbazone derivative **8f** and semicarbazone derivative **7e** were the most potent compounds against MCF-7 cell lines, with IC_50_ values of 5.96 and 5.86 μM, respectively). One of the major indices of a potent effective anti-cancer drug lies in that it can inhibit cancer cell growth and subsequently induce apoptosis. Here, compound **8f** was chosen for subsequent biological functions experiments in MCF-7 cells.

#### 2.2.2. Betulin Derivatives Induced Apoptosis in Various Human Cancer Cells

**Figure 4:**
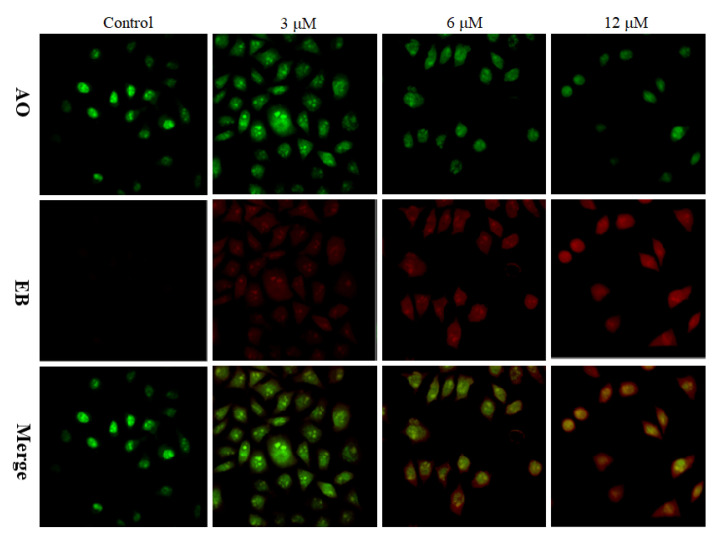
Compound **8f** induced MCF-7 cells apoptosis. MCF-7 cells were co-cultured with compound **8f** (0, 3, 6 and 12 μM) for 24 h and then stained with AO/EB.

Firstly, the Acridine Orange/Ethidium Bromide (AO/EB) staining of MCF-7 cells treated with compound **8f** was observed under a fluorescence microscope. A large number of normal cells in the control group were stained green and their nuclei were intact. As the concentration of compound **8f** increased (0, 3, 6 and 12 μM), some cells showed apoptotic characteristics such as chromosome pyknosis, fragmentation and sparse cytoplasm, and the number of cells gradually increased. Furthermore, the number of early apoptotic cells and late apoptotic cells also increased, the latter was characterized by the nucleus with EB staining, orange red, concentration or bias. The necrotic cells showed uneven orange-red fluorescence and were not clearly defined and disintegrated or nearly disintegrated. The results were shown in Figure 4.

**Figure 5:**
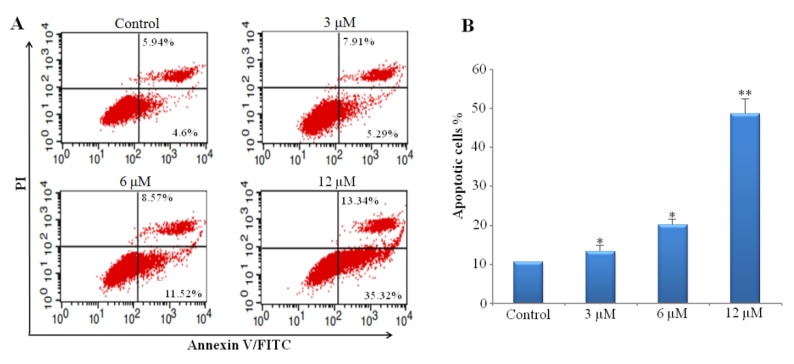
Compound **8f** induced MCF-7 cells apoptosis. (**A**) MCF-7 cells were co-cultured with compound **8f** (0, 3, 6 and 12 μM) for 24 h and then stained with Annexin V-FITC and PI. (**B**) Data were revealed as the means ± SD for independent tests in triplicate. * *p* < 0.05, ** *p* < 0.01 vs. control (**8f** 0 μM) group.

In order to confirm whether tumor cell apoptosis was induced by compound **8f** in tumor cells, the MCF-7 cells were stained in sequence with Annexin V-FITC (AV) and propidium iodide (PI). MCF-7 cells deal with compound **8f** by gradient concentration (0, 3, 6 and 12 μM) for 24 h, and the rates of apoptotic cells were detected by flow cytometry. As shown in Figure 5, after treatment with 3, 6 or 12 μM of **8f** for 24 h, the percentage of apoptosis cells was increased from 13.20% to 48.66%, while the control group’s percentage of apoptosis cells was only 10.54%. Notably, the apoptosis of the MCF-7 cells treatment with compound **8f** increased in a dose-dependent manner. The above results suggested that compound **8f** could induce apoptosis in MCF-7 cells significantly.

#### 2.2.3. Compound **8f** Induced MMP Loss in MCF-7 Cells

**Figure 6:**
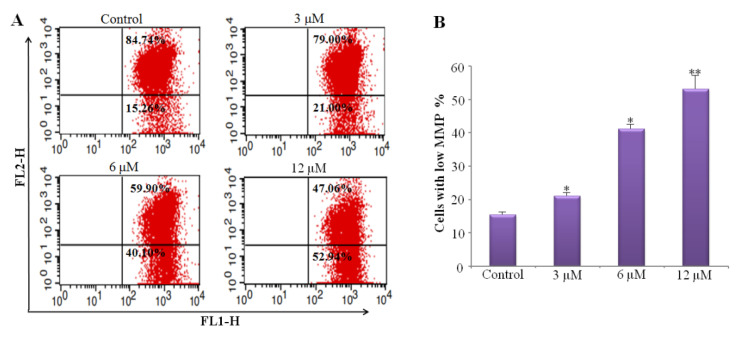
Compound **8f** induced mitochondrial depolarization in MCF-7 cells. (**A**) MCF-7 cells were co-cultured with compound **8f** (0, 3, 6 and 12 μM) for 24 h and then stained with JC-1 cationic dye. (**B**) Data were revealed as the means ± SD for independent tests in triplicate. * *p* < 0.05, ** *p* < 0.01 vs. control (**8f** 0 μM) group.

Mitochondria dysfunction has been proven to be an important role in inducing apoptosis in tumor cells. The loss of mitochondrial membrane potential (MMP) has been considered to be an early manifestation of mitochondrial dysfunction in apoptotic process. Thus, we tried to explore the contribution of mitochondria in compound-**8f**-induced apoptosis in MCF-7 cells. JC-1 cationic dye is an ideal MMP-sensitive probe, which can measure the MMP lost by flow cytometry. As shown in Figure 6, after exposed to compound **8f** from 3.0 to 12.0 μM, the fluorescence intensity decreased from 79.00% to 47.06%, respectively, compared to control group. Notably, the loss of MMP in the MCF-7 cell treatment with compound **8f** occurred in a dose-dependent manner. The results clearly proved that compound **8f** caused MMP to collapse significantly and induced cell apoptosis in MCF-7 cells through the intrinsic mitochondrial-mediated pathways.

#### 2.2.4. Compound **8f** Triggered ROS Generation

**Figure 7:**
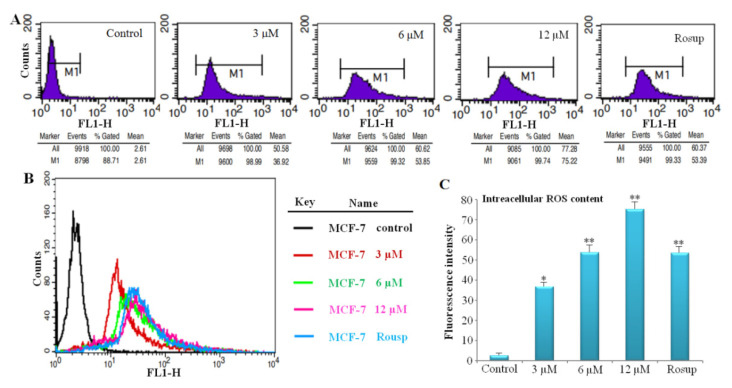
Effects of compound **8f** on intracellular ROS level in MCF-7 cells. MCF-7 cells were co-cultured with **8f** (0, 3, 6 and 12 μM) for 24 h, stained with H_2_DCFDA and analyzed by flow cytometry. (**A**) Representative flow cytometric histograms of ROS level. (**B**) The histograms were merged together. (**C**) The bar graph represents the intracellular ROS level at different concentrations of **8f**. Data were revealed as the means ± SD for independent tests in triplicate. * *p* < 0.05, ** *p* < 0.01 vs. control (**8f** 0 μM) group.

In the past years, many studies reported that intracellular amounts of reactive oxygen species (ROS) play an important role in some kinds of biological processes in tumor cells. In addition, it has been demonstrated that the death-inducing capacity of many chemotherapeutic drugs could be associated with the generation of ROS. Therefore, we explored if ROS stimulated by compound **8f** induced apoptosis in MCF-7 cells. Cells were treated with compound **8f** by gradient concentration (0, 3, 6 and 12 μM) for 24 h and then using H_2_DCFDA staining analysis by flow cytometry. As shown in Figure 7, after exposure to 12 μM of compound **8f** for 24 h, the generation of ROS level was increased to 75.22% compared to the control group (2.61%). Notably, the ROS level of MCF-7 cells treated with compound **8f** increased in a dose-dependent manner. The results demonstrated that compound **8f** significantly induced ROS generation in MCF-7 cells, which could in turn lead to MCF-7 cell apoptosis.

#### 2.2.5. Compound **8f** Regulated Apoptosis-Related Protein Expression

**Figure 8:**
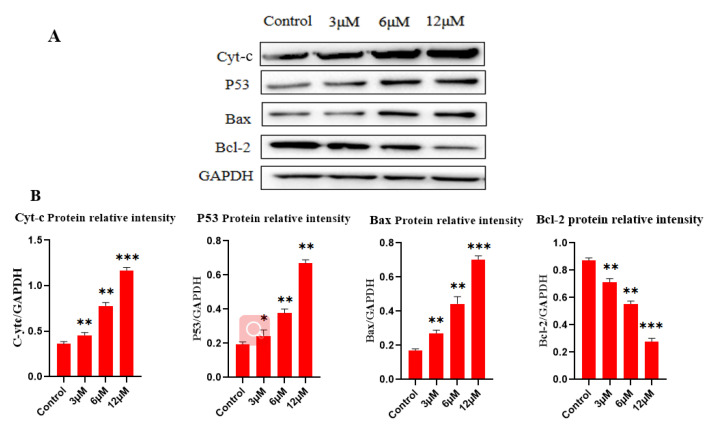
Effects of compound **8f** on the expression of Bax, Bcl-2, Cyt-c and P53 in MCF-7 cells. (**A**) Western blot analysis showing Bax, Bcl-2, Cyt-c and P53 protein expression level. (**B**) Data were revealed as the means ± SD for independent tests in triplicate. * *p* < 0.05, ** *p* < 0.01, *** *p* < 0.001 vs. control (**8f** 0 μM) group.

**Figure 9:**
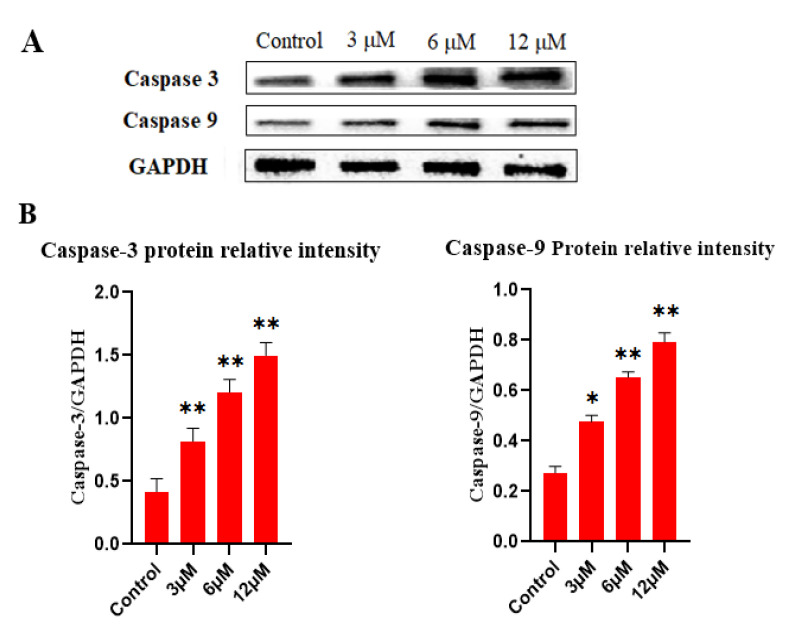
Effects of compound **8f** on the expression of caspase-3 and caspase-9 in MCF-7 cells. (**A**) Western blot analysis showing cleaved caspase-3 and cleaved caspase-9 protein expression level. (**B**) Data were revealed as the means ± SD for independent tests in triplicate. * *p* < 0.05, ** *p* < 0.01 vs. control (**8f** 0 µM) group.

**Figure 10:**
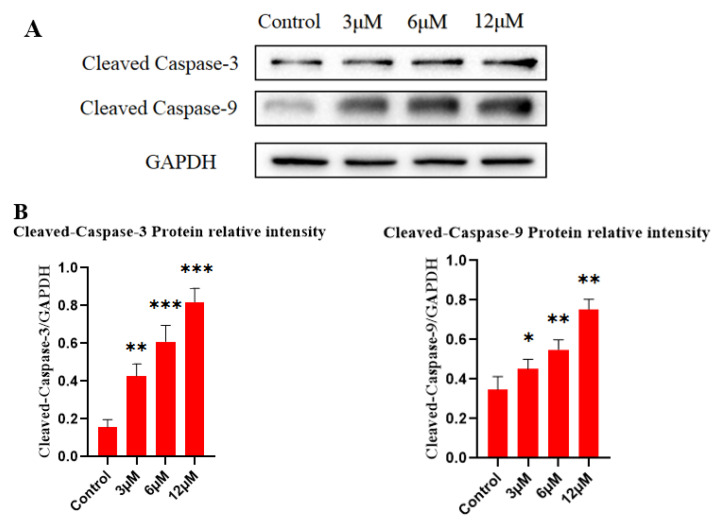
Effects of compound **8f** on the expression of cleaved caspase-3 and cleaved caspase-9 in MCF-7 cells. (**A**). Western blot analysis showing cleaved caspase-3 and cleaved caspase-9 protein expression level. (**B**) Data were revealed as the means ± SD for independent tests in triplicate. * *p* < 0.05, ** *p* < 0.01, *** *p* < 0.001 vs. control (**8f** 0 μM) group.

To further explore the molecular mechanism of compound-**8f**-induced apoptosis in MCF-7 cells, the expression levels of the related apoptotic proteins Bcl-2, Bax, P53, caspase-3, caspase-9, cleaved caspase-3, cleaved caspase-9 and cytochrome c under treatment of compound **8f** were carried out. MCF-7 cells were co-cultured with compound **8f** at the gradient concentrations of 3, 6 and 12 µM for 24 h, and the related apoptotic proteins expression levels were tested by means of Western blotting. The GAPDH expression level was used as an internal control group. As shown in Figure 8, compound **8f** could remarkably suppressed the expression levels of Bcl-2 but increased the expression levels of cytochrome c (cyt-c), P53 and Bax in a dose-dependent manner. As shown in Figure 9, the results indicated that compound **8f** remarkably activated the expression levels of both caspase-3 and caspase-9 in a dose-dependent manner. Meanwhile, the cleavage forms of the caspase proteins were evaluated (Figure 10). Compound **8f** could also activated the expression levels of both cleaved caspase-3 and cleaved caspase-9 in a dose-dependent manner. The above results have further demonstrated that compound **8f** could regulated apoptosis-related protein expression in MCF-7 cells.

## 3. Materials and Methods

### 3.1. Chemistry

All materials and reagents were purchased from commercial suppliers (Energy, Shanghai, China). All unit reaction progress were real-time monitored by TLC using F254 silica gel plates (Biohonor, Guangzhou, China). The intermediates and target derivatives were purified by flash column chromatography (300 mesh silica gel, Yinlong, Qingdao, China). Melting points (mp) of all new derivatives were tested using a MP120 melting point apparatus (Haineng, Fujian, China). NMR spectra were tested using BrukerAvance DRX400 spectrometers 600 MHz for ^1^H NMR and 150 MHz for ^13^C NMR (Bruker, Berlin, Germany). The chemical shifts were expressed in ppm using tetramethylsilane as an internal standard. Low resolution mass spectra were recorded on Esquire 6000 mass spectrometer (Bruker, Berlin, Germany). HRMS spectra were obtained using an Agilent 6250 mass spectrometer (Agilient, San Francisco, CA, USA). The values of MS were recorded in a positive ion mode with ESI source.

#### 3.1.1. Synthesis of 3-O,28-O-Acetyl-betulin (**2**)

To a solution of betulin (**1**, 3.10 g, 7.0 mmol) in pyridine (80 mL) was added 4-dimethylaminopyridine (DMAP, 40 mg, 0.3 mmol) and acetic anhydride (Ac_2_O, 2 mL, 24 mmol) at 0 °C. The reaction was continuously stirred at room temperature for 6 h. The solution was evaporated and then redissolved with CH_2_Cl_2_ (100 mL), washed with sat. NaHCO_3_. The combined solution was washed with brine twice and dried over anhydrous Na_2_SO_4_. The organic solvent was evaporated and then purified by silica gel column chromatography (ethyl acetate/petroleum ether = 1/50) to give **2** (2.95 g, 80%). White solid: mp 216.0–217.5 °C; ^1^H NMR (600 MHz, CDCl_3_) *δ* 4.69 (s, 1H, –C=CH), 4.59 (s, 1H, –C=CH), 4.47 (dd, *J* = 10.6, 5.7 Hz, 1H, AcO–CH), 4.25 (d, *J* = 10.9 Hz, 1H, CH_2_–O), 3.85 (d, *J* = 11.0 Hz, 1H, CH_2_–O), 2.44 (td, *J* = 11.1, 5.8 Hz, 1H), 2.07 (s, 3H, CH_3_–COO), 2.04 (s, 3H, CH_3_–COO), 1.68 (s, 3H, CH_3_–C=C), 1.03 (s, 3H, –CH_3_), 0.97 (s, 3H, –CH_3_), 0.87–0.82 (m, 9H, –CH_3_ × 3), 0.78 (d, *J* = 9.3 Hz, 1H); ^13^C NMR (150 MHz, CDCl_3_) *δ* 171.6, 171.0, 150.2, 109.9, 80.9, 62.8, 55.4, 50.3, 48.8, 47.7, 46.3, 42.7, 40.9, 38.4, 37.8, 37.6, 37.1, 34.5, 34.1, 29.7, 29.6, 27.9, 27.1, 25.2, 23.7, 21.3, 21.1, 20.8, 19.1, 18.2, 16.5, 16.2, 16.0, 14.7; MS (ESI) *m/z*: [M + H]^+^ 527.4.

#### 3.1.2. Synthesis of 3-O-Acetyl-betulin (**3**)

To a solution of 3-*O*,28-*O*-acetyl-betulin (**2**, 3.41 g, 6.4 mmol) in isopropyl alcohol (*i*-PrOH, 160 mL) was added titanium propoxide (Ti(*i*-PrOH)_4_, 10 mL, 35 mmol). The reaction temperature was increased to 85 °C and stirred for 5 h. The solution was evaporated and then CH_2_Cl_2_ (50 mL) and water (50 mL) were added. The filtration was washed with brine twice and dried over anhydrous Na_2_SO_4_. The organic solvent was evaporated and then purified by silica gel column chromatography (ethyl acetate/petroleum ether = 1/7) to give **3** (2.40 g, 76%). White solid: mp 256.1–257.8 °C; ^1^H NMR (600 MHz, CDCl_3_) *δ* 4.68 (d, *J* = 1.8 Hz, 1H, –C=CH), 4.58 (s, 1H, –C=CH), 4.47 (dd, *J* = 11.0, 5.4 Hz, 1H, AcO–CH), 3.85–3.74 (m, 1H, CH_2_-O), 3.33 (d, *J* = 10.8 Hz, 1H, CH_2_-O), 2.38 (td, *J* = 11.0, 5.8 Hz, 1H), 2.04 (s, 3H, CH_3_–COO), 1.69 (s, 3H, CH_3_–C=C), 1.02 (s, 3H, –CH_3_), 0.97 (s, 3H, –CH_3_), 0.88–0.81 (m, 9H, –CH_3_ × 3); ^13^C NMR (150 MHz, CDCl_3_) *δ* 171.0, 150.5, 109.7, 80.9, 60.6, 55.4, 50.3, 48.7, 47.8, 47.8, 42.7, 40.9, 38.4, 37.8, 37.3, 37.1, 34.2, 34.0, 29.7, 29.2, 27.9, 27.0, 25.2, 23.7, 21.3, 20.8, 19.1, 18.2, 16.5, 16.2, 16.0, 14.7; MS (ESI) *m/z*: [M + H]^+^ 485.4.

#### 3.1.3. Synthesis of 3-O-Acetyl-betulinicaldehyde (**4**)

To a solution of 3-*O*-acetyl-betulin (**3**, 300 mg, 0.6 mmol) in CH_2_Cl_2_ (20 mL) was added pyridinium chlorochromate (PCC, 400 mg, 1.8 mmol). The reaction mixture was continuously stirred at 35 °C for 1 h. Then, silica gel (1.50 g) was added into mixture and concentrated to a dry powder. The crude product was purified by silica gel column chromatography to give **4** (240 mg, 80%). White solid: mp 270.2–271.8 °C; ^1^H NMR (600 MHz, CDCl_3_) *δ* 9.67 (d, *J* = 1.1 Hz, 1H, –CHO), 4.76 (d, *J* = 0.9 Hz, 1H, C=C–H), 4.63 (s, 1H, C=C–H), 4.47 (dd, J = 10.9, 5.5 Hz, 1H, AcO–CH), 2.86 (td, *J* = 11.2, 5.9 Hz, 1H), 2.04 (s, 3H, CH_3_–COO), 1.70 (s, 3H, –CH_3_), 0.97 (s, 3H, –CH_3_), 0.91 (s, 3H, –CH_3_), 0.84 (d, *J* = 8.5 Hz, 9H, –CH_3_ × 3); ^13^C NMR (150 MHz, CDCl_3_) *δ* 206.7, 171.0, 149.7, 110.2, 80.9, 59.3, 55.4, 50.4, 48.1, 47.6, 42.6, 40.8, 38.7, 38.4, 37.8, 37.1, 34.3, 33.2, 29.8, 29.2, 28.8, 27.9, 25.5, 23.7, 21.3, 20.7, 19.0, 18.2, 16.5, 16.2, 15.9, 14.2; MS (ESI) *m/z*: [M + H]^+^ 483.4.

#### 3.1.4. Synthesis of Betulinicaldehyde (**5**)

The solution of 3-*O*-acetyl-betulinicaldehyde (**4**, 200 mg, 0.4 mmol) in 2% NaOH-MeOH (10 mL) was continuously stirred for 2 h at 80 °C. The solution was evaporated and then redissolved with CH_2_Cl_2_ (100 mL), washed with brine twice and dried over anhydrous Na_2_SO_4_. The crude product was purified by silica gel column chromatography (ethyl acetate/petroleum ether = 1/10) to give **5** (116 mg, 65%). White solid: mp 285.3–287.2 °C; ^1^H NMR (600 MHz, CDCl_3_) *δ* 9.68 (d, *J* = 1.3 Hz, 1H, –CHO), 4.76 (s, 1H, C=C–H), 4.63 (s, 1H, C=C–H), 3.18 (dd, *J* = 11.5, 4.7 Hz, 1H, O–C–H), 2.86 (td, *J* = 11.2, 5.9 Hz, 1H, C=C–CH–), 1.70 (s, 3H, CH_3_–C=C), 0.97 (s, 3H, –CH_3_), 0.95 (s, 3H, –CH_3_), 0.92 (s, 3H, –CH_3_), 0.82 (s, 3H, –CH_3_), 0.75 (s, 3H, –CH_3_); ^13^C NMR (150 MHz, CDCl_3_) *δ* 206.7, 149.7, 110.2, 79.0, 59.3, 55.3, 50.5, 48.1, 47.5, 42.6, 40.8, 38.8, 38.7, 38.7, 37.2, 34.3, 33.2, 29.9, 29.3, 28.8, 28.0, 27.4, 25.5, 20.8, 19.0, 18.3, 16.1, 15.9, 15.4, 14.3; MS (ESI) *m/z*: [M + H]^+^ 441.4.

#### 3.1.5. Synthesis of 28-Hydrazonomethyl-betulin (**6**)

To a solution of betulinicaldehyde (**5**, 1.55 g, 3.5 mmol) in ethanol (80 mL) was added hydrazine hydrate (85%, 2 mL). The reaction mixture was continuously stirred at 40 °C for 5 h. The solvent was evaporated, and crude product was purified by silica gel column chromatography (ethyl acetate/petroleum ether = 1/1) to give **6** (1.25 g, 70%). White solid: mp 243.5–245.0 °C; ^1^H NMR (600 MHz, CDCl_3_) *δ* 7.25 (d, *J* = 22.3 Hz, 2H, NH_2_), 5.14 (s, 1H, N=CH), 4.71 (s, 1H, C=C–H), 4.59 (s, 1H, C=C–H), 3.18 (dd, *J* = 11.2, 4.2 Hz, 1H, OH), 1.69 (s, 3H, CH_3_), 0.98 (t, *J* = 9.4 Hz, 9H, CH_3_ × 3), 0.93–0.88 (m, 1H), 0.82 (s, 1H, CH_3_), 0.76 (s, 3H, CH_3_), 0.68 (d, *J* = 9.3 Hz, 1H); ^13^C NMR (150 MHz, CDCl_3_) *δ* 150.3, 150.1, 109.8, 79.0, 55.3, 50.4, 50.2, 49.3, 48.0, 42.8, 40.9, 38.9, 38.7, 38.4, 37.3, 37.2, 34.3, 32.7, 30.0, 28.02, 27.9, 27.4, 25.3, 20.9, 19.2, 18.3, 16.1, 16.1, 15.4, 14.7; MS (ESI) *m/z*: [M + H]^+^ 445.4.

#### 3.1.6. General Procedure for Synthesis of Compounds **7a**–**g** and **8a**–**g**

To a solution of 28-hydrazonomethyl-betulin (**6**, 1 mmol) in ethanol (20 mL) was added phenyl isocyanate or phenyl isothiocyanate substituent (2 mmol) and five drops of acetic acid. The reaction mixture was continuously stirred at 30 °C for 5~10 h until no material. The solvent was evaporated, and crude product was purified by silica gel column chromatography (dichloromethane/methanol) to obtain compounds **7a**–**g** and **8a**–**g**. 

*Lup-20(29)-ene-3β-ol-28-N-(4-chlorophenyl)semicarbazide (***7a***)*. White solid: yield 81%; m.p. 227.2–228.3 °C; ^1^H NMR (600 MHz, CDCl_3_) *δ* 9.07 (s, 1H, 28-H), 8.01 (s, 1H, NH), 7.46 (d, *J* = 8.7 Hz, 2H, Ar–H), 7.36 (s, 1H, NH), 7.28 (d, *J* = 8.7 Hz, 2H, Ar–H), 4.74 (s, 1H, H-29), 4.64 (s, 1H, H-29), 3.18 (dd, *J* = 11.5, 4.6 Hz, 1H, H-3), 1.71 (s, 3H, 30-CH_3_), 1.01 (s, 3H, 27-CH_3_), 0.97 (s, 3H, 26-CH_3_), 0.92 (s, 3H, 25-CH_3_), 0.80 (s, 3H, 24-CH_3_), 0.76 (s, 3H, 23-CH_3_); ^13^C NMR (150 MHz, CDCl_3_) *δ* 153.6, 149.5, 136.7, 129.0, 128.2, 120.5, 110.3, 78.9, 55.3, 50.7, 50.3, 49.4, 48.2, 42.8, 40.9, 38.9, 38.7, 38.7, 37.2, 37.0, 34.3, 32.7, 29.9, 28.0, 27.9, 27.4, 25.2, 20.8, 19.1, 18.3, 16.1, 16.1, 15.4, 14.7; HRMS calculated for C_37_H_54_ClN_3_O_2_ 608.3983 [M + H]^+^; found: *m/z* 608.3980.

*Lup-20(29)-ene-3β-ol-28-N-(4-cyanophenyl)semicarbazide (***7b***)*. White solid; yield 75%: m.p. 236.5–238.0 °C; ^1^H NMR (600 MHz, CDCl_3_) *δ* 9.16 (s, 1H, H-28), 8.26 (s, 1H, NH), 7.62 (dd, *J* = 20.9, 8.6 Hz, 4H, Ar–H), 7.40 (s, 1H, NH), 4.74 (s, 1H, H-29), 4.65 (s, 1H, H-29), 3.19 (dd, *J* = 11.4, 4.3 Hz, 1H, OH), 2.49 (s, 1H, H-3), 1.72 (s, 3H, 30-CH_3_), 1.01 (s, 3H, 27-CH_3_), 0.97 (s, 3H, 26-CH_3_), 0.92 (s, 3H, 25-CH_3_), 0.79 (s, 3H, 24-CH_3_), 0.76 (s, 3H, 23-CH_3_); ^13^C NMR (150 MHz, CDCl_3_) *δ* 153.1, 150.5, 149.4, 142.3, 133.3, 118.9, 118.8, 110.5, 106.1, 78.9, 55.3, 50.8, 50.3, 49.3, 48.3, 42.8, 40.9, 38.8, 38.7, 38.7, 37.1, 37.0, 34.3, 32.6, 29.9, 29.7, 28.0, 27.9, 27.4, 25.2, 20.8, 19.1, 18.3, 16.1, 16.1, 15.4, 14.7; HRMS calculated for C_38_H_54_N_4_O_2_ 599.4325 [M + H]^+^; found: *m/z* 599.4322.

*Lup-20(29)-ene-3β-ol-28-N-(4-methoxyphenyl)semicarbazide (***7c***)*. White solid: yield 88%; m.p. 214.2–216.0 °C. ^1^H NMR (600 MHz, CDCl_3_) *δ* 9.08 (s, 1H, H-28), 7.86 (s, 1H, NH), 7.40 (d, *J* = 8.8 Hz, 2H, Ar–H), 7.34 (s, 1H, NH), 6.87 (d, *J* = 8.9 Hz, 2H, Ar-H), 4.74 (s, 1H, H-29), 4.63 (s, 1H, H-29), 3.79 (s, 3H, OCH_3_), 3.18 (dd, *J* = 11.5, 4.6 Hz, 1H, OH), 2.51 (td, *J* = 10.9, 5.7 Hz, 1H, H-3), 1.72 (d, *J* = 10.0 Hz, 3H, 30-CH_3_), 1.00 (s, 3H, 27-CH_3_), 0.97 (s, 3H, 26-CH_3_), 0.94 (s, 3H, 25-CH_3_), 0.80 (s, 3H, 24-CH_3_), 0.75 (s, 3H, 23-CH_3_); ^13^C NMR (150 MHz, CDCl_3_) *δ* 155.9, 154.1, 149.7, 148.9, 131.2, 121.3, 114.3, 110.2, 78.9, 55.5, 55.3, 50.6, 50.4, 49.4, 48.2, 42.8, 40.9, 38.8, 38.7, 38.6, 37.2, 37.1, 34.3, 32.7, 29.9, 28.0, 27.9, 27.4, 25.3, 20.8, 19.1, 18.3, 16.1, 16.0, 15.4, 14.7; HRMS calculated for C_38_H_57_N_3_O_3_ 604.4478 [M + H]^+^; found: *m/z* 604.4475.

*Lup-20(29)-ene-3β-ol-28-N-(4-(trifluoromethyl)phenyl)semicarbazide (***7d***)*. Yellowish solid: yield 78%; m.p. 221.5–222.7 °C; ^1^H NMR (600 MHz, DMSO) *δ* 10.42 (s, 1H, H-28), 8.76 (s, 1H, NH), 7.81 (d, *J* = 8.4 Hz, 2H, Ar–H), 7.62 (d, *J* = 8.6 Hz, 2H, Ar–H), 7.57 (s, 1H, NH), 4.72 (s, 1H, H-29), 4.59 (s, 1H, H-29), 4.26 (s, 1H, OH), 2.97 (dd, *J* = 9.7, 6.0 Hz, 1H, H-3), 1.68 (s, 3H, 30-CH_3_), 0.97 (s, 3H, 27-CH_3_), 0.93 (s, 3H, 26-CH_3_), 0.87 (s, 3H, 25-CH_3_), 0.76 (s, 3H, 24-CH_3_), 0.65 (s, 3H, 23-CH_3_); ^13^C NMR (150 MHz, CDCl_3_) *δ* 152.1, 148.5, 138.2, 133.7, 128.9, 122.3, 118.2, 116.1, 109.3, 77.9, 54.3, 49.7, 49.3, 48.3, 47.1, 41.8, 39.8, 37.8, 37.7, 37.6, 36.1, 33.2, 31.5, 28.8, 26.9, 26.8, 26.3, 24.2, 22.5, 19.7, 18.1, 17.2, 15.0, 14.9, 14.3, 13.6; HRMS calculated for C_38_H_54_F_3_N_3_O_2_ 642.4246 [M + H]^+^; found: *m/z* 642.4243.

*Lup-20(29)-ene-3β-ol-28-N-(3-(trifluoromethyl)phenyl)semicarbazide (***7e***)*. Yellowish solid: yield 75%; m.p. 223.4–225.1 °C; ^1^H NMR (600 MHz, DMSO) δ 10.39 (s, 1H, H-28), 8.75 (s, 1H, NH), 8.06 (s, 1H, NH), 7.85 (d, *J* = 8.2 Hz, 1H, Ar-H), 7.57 (s, 1H, Ar–H), 7.50 (t, *J* = 8.0 Hz, 1H, Ar-H), 7.33 (d, *J* = 7.8 Hz, 1H, Ar–H), 4.72 (s, 1H, H-29), 4.59 (s, 1H, H-29), 4.23 (dd, *J* = 18.8, 12.2 Hz, 1H, OH), 2.97 (dd, *J* = 9.8, 6.1 Hz, 1H, H-3), 1.68 (s, 3H, 30-CH_3_), 0.97 (s, 3H, 27-CH_3_), 0.94 (s, 3H, 26-CH_3_), 0.87 (s, 3H, 25-CH_3_), 0.76 (s, 3H, 24-CH_3_), 0.65 (s, 3H, 23-CH_3_); ^13^C NMR (150 MHz, DMSO) *δ* 153.4, 150.1, 149.7, 149.7, 140.5, 130.0, 129.8, 123.5, 118.9, 115.9, 110.5, 77.2, 55.3, 50.3, 50.2, 49.3, 48.3, 42.8, 40.9, 40.5, 38.9, 38.7, 38.4, 37.1, 36.6, 34.3, 32.7, 29.8, 28.5, 28.0, 27.6, 25.2, 20.8, 19.2, 18.4, 16.36, 16.26, 14.91; HRMS calculated for C_38_H_54_F_3_N_3_O_2_ 642.4246 [M + H]^+^; found: *m/z* 642.4243.

*Lup-20(29)-ene-3β-ol-8-N-(m-tolyl)semicarbazide (***7f***).* Yellowish solid: yield 79%; m.p. 224.2–226.0 °C; ^1^H NMR (600 MHz, CDCl_3_) *δ* 8.96 (s, 1H, H-28), 7.97 (s, 1H, NH), 7.32 (dd, *J* = 15.2, 9.9 Hz, 3H, Ar–H), 7.20 (t, *J* = 7.8 Hz, 1H, Ar–H), 6.89 (d, *J* = 7.5 Hz, 1H, NH), 4.74 (s, 1H, H-29), 4.63 (s, 1H, H-29), 3.18 (dd, *J* = 11.5, 4.7 Hz, 1H, OH), 2.53 (td, *J* = 11.0, 5.8 Hz, 1H, H-3), 2.35 (s, 3H, Ar–CH_3_), 1.71 (s, 3H, 30-CH_3_), 1.00 (s, 3H, 27-CH_3_), 0.96 (s, 3H, 26-CH_3_), 0.93 (s, 3H, 25-CH_3_), 0.79 (s, 3H, 24-CH_3_), 0.75 (s, 3H, 23-CH_3_); ^13^C NMR (150 MHz, CDCl_3_) *δ* 153.7, 149.6, 149.0, 138.8, 137.9, 128.8, 124.1, 119.9, 116.4, 110.2, 79.0, 55.3, 50.7, 50.4, 49.3, 48.1, 42.8, 40.9, 38.8, 38.7, 38.6, 37.2, 37.1, 34.3, 32.6, 29.9, 28.0, 27.9, 27.4, 25.3, 21.5, 20.8, 19.2, 18.2, 16.1, 16.0, 15.4, 14.7; HRMS calculated for C_38_H_57_N_3_O_2_ 588.4529 [M + H]^+^; found: *m/z* 588.4526.

*Lup-20(29)-ene-3β-ol-28-N-((3-trifluoromethyl-4-chloro)phenyl)semicarbazide (***7g***)*. Yellowish solid: yield 84%; m.p. 211.6–213.0 °C; ^1^H NMR (600 MHz, DMSO) *δ* 10.42 (s, 1H, H-28), 8.86 (s, 1H, NH), 8.19 (d, *J* = 2.4 Hz, 1H, NH), 7.92 (dd, J = 8.8, 2.0 Hz, 1H, Ar–H), 7.60 (m, 2H, Ar–H), 4.72 (s, 1H, H-29), 4.59 (s, 1H, H-29), 4.24 (d, *J* = 5.1 Hz, 1H, OH), 3.01–2.93 (m, 1H, H-3), 1.67 (s, 3H, 30-CH_3_), 0.97 (s, 3H, 27-CH_3_), 0.93 (s, 3H, 26-CH_3_), 0.87 (s, 3H, 25-CH_3_), 0.76 (s, 3H, 24-CH_3_), 0.65 (s, 3H, 23-CH_3_); ^13^C NMR (151 MHz, DMSO) *δ* 153.3, 150.1, 139.3, 132.1, 127.0, 126.8, 124.8, 124.2, 123.3, 122.4, 118.7, 110.5, 77.3, 55.3, 50.3, 50.2, 49.3, 48.3, 42.8, 40.9, 40.5, 38.9, 38.7, 38.4, 37.2, 34.4, 32.7, 29.8, 28.0, 27.6, 25.3, 20.8, 19.2, 18.4, 16.3, 16.2, 16.2, 14.9; HRMS calculated for C_38_H_53_ClF_3_N_3_O_2_ 676.3857 [M + H]^+^; found: *m/z* 676.3854.

*Lup-20(29)-ene-3β-ol-28-N-(4-fluorophenyl)thiosemicarbazide (***8a***)*. Yellowish solid: yield 86%; m.p. 209.3–211.0 °C; ^1^H NMR (600 MHz, CDCl_3_) *δ* 9.68 (s, 1H, H-28), 8.95 (s, 1H, NH), 7.57 (dd, *J* = 8.9, 4.8 Hz, 2H, Ar–H), 7.52 (s, 1H, NH), 7.07 (t, *J* = 8.6 Hz, 2H, Ar–H), 4.73 (s, 1H, H-29), 4.63 (s, 1H, H-29), 3.19 (dd, *J* = 11.5, 4.6 Hz, 1H, OH), 2.46–2.37 (m, 1H, H-3), 1.70 (s, 3H, 30-CH_3_), 0.97 (s, 3H, 27-CH_3_), 0.95 (s, 3H, 26-CH_3_), 0.82 (s, 3H, 25-CH_3_), 0.77 (s, 3H, 24-CH_3_), 0.67 (s, 1H, 23-CH_3_); ^13^C NMR (150 MHz, CDCl_3_) *δ* 159.8, 149.3, 133.9, 133.8, 126.5, 126.4, 110.5, 78.9, 55.3, 50.9, 50.4, 49.4, 48.3, 42.9, 40.9, 38.9, 38.8, 38.7, 37.2, 36.9, 34.3, 32.7, 29.7, 29.7, 28.0, 27.9, 27.4, 25.2, 21.0, 20.8, 19.1, 18.3, 16.3, 16.1, 15.4, 14.7; HRMS calculated for C_37_H_54_FN_3_OS 608.4050 [M + H]^+^; found: *m/z* 608.4047.

*Lup-20(29)-ene-3β-ol-28-N-(4-chlorophenyl)thiosemicarbazide (***8b***)*. Yellowish solid: yield 87%; m.p. 212.5–214.0 °C; ^1^H NMR (600 MHz, CDCl_3_) *δ* 9.68 (s, 1H, H-28), 9.06 (s, 1H, NH), 7.61 (d, *J* = 8.6 Hz, 2H, Ar–H), 7.50 (s, 1H, NH), 7.34 (d, *J* = 8.5 Hz, 2H, Ar–H), 4.72 (s, 1H, H-29), 4.63 (s, 1H, H-29), 3.19 (dd, *J* = 11.4, 4.4 Hz, 1H, OH), 2.44 (s, 1H, H-3), 1.70 (s, 3H, 30-CH_3_), 1.00 (s, 3H, 27-CH_3_), 0.97 (s, 3H, 26-CH_3_), 0.95 (s, 3H, 25-CH_3_), 0.82 (s, 3H, 24-CH_3_), 0.77 (s, 3H, 23-CH_3_); ^13^C NMR (151 MHz, CDCl_3_) *δ* 155.9, 149.3, 136.4, 131.3, 128.8, 125.5, 110.5, 78.9, 55.3, 50.9, 50.8, 50.4, 49.5, 48.3, 42.9, 40.9, 38.8, 38.8, 38.7, 37.2, 36.8, 34.3, 32.7, 29.8, 28.0, 27.9, 27.4, 25.2, 20. 8, 19.1, 18.3, 16.3, 16.1, 15.4, 14.7; HRMS calculated for C_37_H_54_ClN_3_OS 624.3754 [M + H]^+^; found: *m/z* 624.3752.

*Lup-20(29)-ene-3β-ol-28-N-(p-tolyl)thiosemicarbazide (***8c***)*. White solid: yield 74%; m.p. 206.4–208.2 °C; ^1^H NMR (600 MHz, CDCl_3_) *δ* 9.62 (s, 1H, H-28), 8.96 (s, 1H, NH), 7.49 (d, *J* = 2.5 Hz, 2H, Ar–H), 7.48 (s, 1H, NH), 7.18 (d, *J* = 8.2 Hz, 2H, Ar–H), 4.73 (s, 1H, H-29), 4.63 (s, 1H, H-29), 3.19 (dd, *J* = 11.5, 4.7 Hz, 1H, OH), 2.46 (dd, *J* = 10.6, 4.8 Hz, 1H, H-3), 2.35 (s, 3H, Ar-CH_3_), 1.70 (s, 3H, 30-CH_3_), 1.00 (s, 3H, 27-CH_3_), 0.97 (s, 3H, 26-CH_3_), 0.96 (s, 3H, 25-CH_3_), 0.82 (s, 3H, 24-CH_3_), 0.77 (s, 3H, 23-CH_3_); ^13^C NMR (150 MHz, CDCl_3_) *δ* 175.9, 151.2, 149.3, 135.9, 135.3, 129.3, 124.4, 110.4, 78.9, 55.3, 50.9, 50.8, 50.4, 49.4, 48.2, 42.9, 40.9, 38.9, 38.8, 38.7, 37.2, 36.9, 34.3, 32.6, 29.8, 29.7, 28.0, 27.9, 27.4, 25.2, 21.0, 20.8, 19.1, 18.3, 16.3, 16.1, 15.4, 14.7; HRMS calculated for C_38_H_57_N_3_OS 604.4301 [M + H]^+^; found: *m/z* 604.4297.

*Lup-20(29)-ene-3β-ol-28-N-(4-methoxyphenyl)thiosemicarbazide (***8d***)*. Yellowish solid: yield 88%; m.p. 203.7–205.2 °C; ^1^H NMR (600 MHz, CDCl3) *δ* 9.61 (s, 1H, H28), 8.88 (s, 1H, NH), 7.49 (d, *J* = 3.9 Hz, 2H, Ar–H), 7.47 (s, 1H, NH), 6.92 (d, *J* = 8.9 Hz, 2H, Ar–H), 4.74 (d, *J* = 15.6 Hz, 1H, H-29), 4.63 (s, 1H, H-29), 3.82 (s, 3H, OCH_3_), 3.19 (dd, *J* = 11.5, 4.7 Hz, 1H, OH), 2.45 (td, *J* = 10.8, 6.2 Hz, 1H, H-3), 1.70 (s, 3H, 30-CH_3_), 1.00 (s, 3H, 27-CH_3_), 0.97 (s, 3H, 26-CH_3_), 0.96 (s, 3H, 25-CH_3_), 0.82 (s, 3H, 24-CH_3_), 0.77 (s, 3H, 23-CH_3_); ^13^C NMR (150 MHz, CDCl_3_) *δ* 176.36, 157.90, 151.24, 149.37, 130.85, 126.38, 114.01, 110.4, 78.9, 55.5, 55.3, 50.9, 50.8, 50.4, 49.4, 48.2, 42.9, 40.9, 38.9, 38.8, 38.7, 37.2, 36.9, 34.3, 32.7, 29.8, 29.7, 28.0, 27.9, 27.4, 25.2, 20.8, 19.1, 18.3, 16.2, 16.1, 15.4, 14.7; HRMS calculated for C_38_H_57_N_3_O_2_S 620.4250 [M + H]^+^; found: *m/z* 620.4247.

*Lup-20(29)-ene-3β-ol-28-N-(4-tert-butylphenyl)thiosemicarbazide (***8e***)*. Yellowish solid: yield 72%; m.p. 216.8–218.2 °C; ^1^H NMR (600 MHz, CDCl_3_) *δ* 9.58 (s, 1H, H-28), 9.01 (s, 1H, NH), 7.56 (d, *J* = 8.6 Hz, 2H, Ar–H), 7.49 (s, 1H, NH), 7.44–7.36 (m, 2H, Ar–H), 4.73 (d, *J* = 1.2 Hz, 1H, H-29), 4.63 (s, 1H, H-29), 3.19 (dd, *J* = 11.5, 4.7 Hz, 1H, OH), 2.47 (td, *J* = 10.9, 6.1 Hz, 1H, H-3), 1.70 (s, 3H, 30-CH_3_), 1.33 (s, 9H, t-Bu), 1.01 (s, 3H, 27-CH_3_), 0.98 (s, 3H, 26-CH_3_), 0.97 (s, 3H, 25-CH_3_), 0.83 (s, 3H, 24-CH_3_), 0.76 (s, 3H, 23-CH_3_); ^13^C NMR (150 MHz, CDCl_3_) *δ* 175.6, 151.1, 149.3, 149.0, 135.2, 125.7, 123.7, 110.4, 78.9, 60.4, 55.3, 50.9, 50.4, 49.4, 48.2, 42.9, 40.9, 38.8, 38.8, 38.7, 37.2, 36.9, 34.5, 34.3, 32.6, 31.3, 29.8, 29.7, 28.0, 27.9, 27.4, 25.2, 20.8, 19.1, 18.3, 16.2, 16.1, 15.4, 14.7, 14.2; HRMS calculated for C_41_H_63_N_3_OS 646.4770 [M + H]^+^; found: *m/z* 646.4767.

*Lup-20(29)-ene-3β-ol-28-N-(3-chlorophenyl)thiosemicarbazide (***8f***)*. Yellowish solid: yield 80%; m.p. 209.8–212.0 °C; ^1^H NMR (600 MHz, CDCl_3_) *δ* 9.72 (s, 1H, H-28), 9.00 (s, 1H, NH), 7.70 (t, *J* = 1.9 Hz, 1H, NH), 7.49 (dd, *J* = 10.6, 1.4 Hz, 2H, Ar–H), 7.23 (t, *J* = 8.1 Hz, 1H, Ar–H), 7.12 (dd, *J* = 8.0, 1.0 Hz, 1H, Ar–H), 4.66 (s, 1H, H-29), 4.56 (s, 1H, H-29), 3.12 (dd, *J* = 11.5, 4.6 Hz, 1H, OH), 2.38 (td, *J* = 10.6, 6.0 Hz, 1H, H-3), 1.63 (s, 3H, 30-CH_3_), 0.94 (s, 3H, 27-CH_3_), 0.90 (s, 3H, 26-CH_3_), 0.88 (s, 3H, 25-CH_3_), 0.74 (s, 3H, 24-CH_3_), 0.70 (s, 3H, 23-CH_3_); ^13^C NMR (150 MHz, CDCl_3_) *δ* 174.3, 150.9, 148.2, 138.0, 133.3, 128.6, 124.8, 122.7, 120.8, 109.5, 77.9, 54.3, 49.9, 49.3, 48.4, 47.2, 41.9, 39.9, 37.8, 37.8, 37.7, 36.1, 35.8, 33.4, 31.6, 28.8, 27.0, 26.9, 26.4, 24.2, 19.7, 18.0, 17.2, 15.2, 15.1, 14.4, 13.7; HRMS calculated for C_37_H_54_ClN_3_OS 624.3754 [M + H]^+^; found: *m/z* 624.3752.

*Lup-20(29)-ene-3β-ol-28-N-(3,4-dichlorophenyl)thiosemicarbazide (***8g***)*. Yellowish solid: yield 77%; m.p. 219.8–221.8 °C; ^1^H NMR (600 MHz, CDCl3) *δ* 9.92 (s, 1H, H-28), 9.03 (s, 1H, NH), 7.88 (d, *J* = 2.4 Hz, 1H, Ar–H), 7.58 (s, 1H, Ar–H), 7.53 (dd, *J* = 8.7, 2.4 Hz, 1H, NH), 7.43 (d, *J* = 8.7 Hz, 1H, Ar–H), 4.73 (s, 1H, H-29), 4.63 (s, 1H, H-29), 3.19 (dd, *J* = 11.5, 4.7 Hz, 1H, OH), 2.43 (td, *J* = 10.9, 6.0 Hz, 1H, H-3), 1.70 (s, 3H, 30-CH_3_), 1.00 (s, 3H, 27-CH_3_), 0.97 (s, 3H, 26-CH_3_), 0.94 (s, 3H, 25-CH_3_), 0.81 (s, 3H, 24-CH_3_), 0.77 (s, 3H, 23-CH_3_); ^13^C NMR (150 MHz, CDCl_3_) *δ* 175.3, 152.4, 149.2, 137.3, 132.5, 130.2, 129.3, 125.4, 123.1, 110.5, 78.9, 60.4, 55.3, 51.0, 50.3, 49.4, 48.3, 42.9, 40.9, 38.7, 37.1, 36.7, 34.3, 32.6, 29.7, 29.3, 28.0, 27.9, 25.2, 21.1, 20.7, 19.0, 18.2, 16.2, 16.1, 15.4, 14.7; HRMS calculated for C_37_H_53_Cl_2_N_3_OS 658.3359 [M + H]^+^; found: *m/z* 658.3362.

### 3.2. Biological Evaluation

#### 3.2.1. In Vitro Cytotoxicity

All cell lines were purchased from Shanghai cell Bank of the Chinese Academy of Science. Cells were cultured in RPMI-1640 medium added with 100 units/mL of penicillin, 10% FBS and 100 μg/mL streptomycin at 37 °C in an atmosphere of 5% CO_2_. Cytotoxic activities of all tested compounds against four cell lines were evaluated by MTT assay. Cells in the logarithmic growth phase were cultured in 96-well plates (1 × 10^4^ cells per well) for 24 h. Then the cells were co-cultured with compounds at different concentrations (from 5 μM to 80 μM) for 48 h, then 10 μL MTT solution (5 mg/mL in PBS, Sigma Chemical Co., Ltd., St. Louis MO, USA) was added for 2 h. The solution was removed and then added 100 μL DMSO, and the absorbance (OD value) was recorded at 490 nm using microplate reader (Bio-Rad iMARK, Hercules, CA, USA). The IC_50_ values were calculated by SPSS nonlinear regression analysis (IBM SPSS Statistics for Windows, version 20.0; IBM Corp., Armonk, NY, USA, 2013) [55,56,57].

#### 3.2.2. AO/EB Staining

The MCF-7 cells in the logarithmic growth phase were cultured into 6-well plates (5 × 10^4^ cells/mL). The cells incubated for overnight at 37 °C in an atmosphere of 5% CO_2_. Then the MCF-7 cells were treated with compound **8f** (0, 3, 6 and 12 μM) for 24 h. The cover slip with monolayer MCF-7 cells was inverted on the glass slide with 20 μL of AO/BE stain (100 μg/mL). The fluorescence intensity was read using a fluorescence microscope (Zeiss, Oberserve A1, Oberkochen, Germany) [58,59].

#### 3.2.3. Cell Apoptosis Analysis

Apoptosis was determined by staining cells with an Annexin VFITC/PI detection kit (BD Biosciences, San Jose, CA, USA). Briefly, MCF-7 cells were seeded on six-well plates (6 × 10^4^ cells/mL) and incubated with compound **8f** (0, 3, 6 and 12 μM) for 24 h. Cells were washed twice with cold PBS and resuspended in 500 μL of 1 × binding buffer, and then 10 μL of Annexin V-FITC and 5 μL of PI were applied to stain the cells for 15 min at room temperature in the dark. The status of stained cells was analyzed using a flow cytometer (BD, FACSCalibur, San Jose, CA, USA) [60,61]. 

#### 3.2.4. Mitochondrial Membrane Potential (MMP) Assay

MCF-7 cells were cultured into 12-well plates at a concentration of 1 × 10^6^ cells/well of the RPMI-1640 medium. Then, cells were co-cultured with compound **8f** (0, 3, 6 and 12 µM) for 24 h. After treatment, cells were stained with 3 µM cationic dye JC-1 at room temperature in the dark for 30 min according to the manufacturer’s instructions (BD Biosciences, San Jose, CA, USA). Finally, cells were harvested and washed with PBS twice, and then the results were recorded by flow cytometry analysis [62].

#### 3.2.5. ROS Level Assay

MCF-7 cells were cultured into 12-well plate at a concentration of 1 × 10^6^ cells/well for 24 h of the RPMI-1640 medium with 10% FBS. Then, cells were co-cultured with compound **8f** (0, 3, 6 and 12 µM) for 24 h. Then, the MCF-7 cells were stained with 5 µM H_2_DCFDA solution (400 µL/well) at room temperature in the dark for 30 min. The cells were harvested and washed with PBS twice according to the manufacturer’s instructions (Thermo Fisher Scientific, Waltham, MA, USA). The results were obtained by flow cytometry [63].

#### 3.2.6. Western Blot Assay

After treatment with compound **8f** (0, 3, 6 and 12 µM), MCF-7 cells were harvested and lysed in RIPA buffer and boiled for 10 min at 100 °C. Equal amount of protein (30 mg) were separated on a 10% SDS-PAGE gel and transferred to nitrocellulose membranes. The membranes were blocked with 5% BSA and probed with a 1:1000 dilution of primary antibody against Cl-caspase-3 (Cell Singaling Technology, Inc., Danvers, MA, USA), Cl-caspase-9 (Cell Singaling Technology Inc), P53 (Cell Singaling Technology, Inc; Danvers, MA, USA), Bcl-2 (Santa Cruz Biotechnology, Inc; Santa Cruz, CA, USA), Cytochrome c (Proteintech Group, Inc; Rosemont, IL, USA) and Bax (Proteintech Group, Inc; Rosemont, IL, USA). Then, the membranes were incubated with a 1:5000 dilution of horseradish peroxidase-conjugated secondary antibody for 2 h. Positive bands were visualized on a X-ray film using an enhanced chemiluminescence system (Kodak) [64,65].

## 4. Conclusions

In summary, according to the special structural features of betulin and thio-/semicarbazone groups, 14 new betulin derivatives with thiocarbazone or semicarbazone sidechains on the C-28 position were synthesized. All new compounds were evaluated for their in vitro cytotoxicities in human carcinoma cells (HepG, MCF-7, A549, HCT-116) and normal human gastric epithelial cells (GES-1). Among them, compound **8f** displayed the most potent cytotoxicity, with an IC_50_ value of 5.86 ± 0.61 μM against MCF-7 cells. Furthermore, the intracellular mechanism studies proved that compound **8f** could trigger the mitochondrial-mediated apoptosis pathway by losing MMP, which was associated with the downregulation of Bcl-2 and P53, the upregulation of Bax expression, the activation of the levels of caspase-3 and caspase-9, and the formation of ROS. The above results indicated that compound **8f** could be used as a valuable skeleton for developing novel antitumor agents.

## Figures and Tables

**Table 1:** Cytotoxicities of compounds in human cancer and normal cells.

Compound	X ^b^	R	IC_50_ (μM) ^a^				
			HepG2	MCF-7	A549	HCT-116	GES-1
**7a**	O	4-Cl	11.64 ± 0.62	8.57 ± 0.60	18.07 ± 1.03	34.80 ± 1.25	70.82 ± 2.12
**7b**	O	4-CN	15.43 ± 0.75	7.88 ± 0.54	25.14 ± 1.01	51.06 ± 2.52	85.36 ± 3.37
**7c**	O	4-OCH_3_	46.70 ± 1.68	44.35 ± 2.26	>80	>80	-
**7d**	O	4-CF_3_	12.60 ± 0.67	15.33 ± 1.23	29.42 ± 1.12	28.60 ± 1.36	168.32 ± 4.59
**7e**	O	3-CF_3_	8.93 ± 0.63	5.96 ± 0.80	43.77 ± 1.44	26.14 ± 1.17	196.18 ± 5.82
**7f**	O	3-CH_3_	49.02 ± 1.96	26.82 ± 1.34	>80	>80	-
**7g**	O	3-CF_3_-4-Cl	11.04 ± 1.02	7.06 ± 0.44	33.64 ± 1.15	13.46 ± 0.74	144.63 ± 3.88
**8a**	S	4-F	12.42± 0.48	9.80 ± 0.58	24.05 ± 1.11	32.53 ± 1.26	176.62 ± 5.02
**8b**	S	4-Cl	8.40 ± 0.34	7.79 ± 0.45	18.66 ± 0.98	49.51 ± 1.44	203.32 ± 5.74
**8c**	S	4-CH_3_	40.25 ± 1.28	28.56 ± 1.06	>80	46.31 ± 1.77	-
**8d**	S	4-OCH_3_	32.50 ± 1.17	38.37 ± 1.58	>80	>80	-
**8e**	S	4-*t*-Bu	>80	46.36 ± 3.42	>80	>80	-
**8f**	S	3-Cl	6.87 ± 0.76	5.86 ± 0.61	10.36 ± 0.74	24.65 ± 0.90	214.60 ± 6.28
**8g**	S	3,4-di-Cl	8.74 ± 0.48	11.30 ± 1.07	16.85 ± 1.05	20.15 ± 0.87	174.50 ± 5.14
**1**			20.42± 1.22	19.43 ± 0.93	27.06 ± 1.20	30.02 ± 1.22	108.07 ± 4.34
Mitomycin			27.42 ± 1.24	15.88 ± 1.03	12.84 ± 1.08	11.36 ± 0.87	20.54 ± 1.02

^a^ IC_50_: Concentration of the tested compound that inhibits 50% of cell growth. All data are recorded as means ± SD of three experiments. ^b^ X means oxygen atom (O) when the substituent is semicarbazone; X means sulphur atom (S) when the substituent is thiosemicarbazone. “-” not active.

## Data Availability

The datasets used and/or analyzed during the current study are available from the corresponding author on reasonable request.

## Supplementary Materials

The following are available online. NMR and HRMS spectra of new compounds.
