# Supplementary material for: Design and Synthesis of Novel Betulin Derivatives Containing Thio-/Semicarbazone Moieties as Apoptotic Inducers through Mitochindria-Related Pathways
Source: Molecules. 2021 Oct 21;26(21):6356. doi: 10.3390/molecules26216356 (PMC8587101; doi:10.3390/molecules26216356)
Supplement: Supplementary file 1 [file molecules-26-06356-s001.zip › molecules-1370739-supplementary.pdf]

Supplementary Material

Design and Synthesis of Novel Betulin Derivatives Containing Thio-/Semicarbazone Moieties as Apoptotic Inducers Through Mitochindria-related Pathways

Jiafeng Wang [1], Jiale Wu [1], Yinglong Han [1], Jie Zhang [1], Yu Lin [1], Haijun Wang [1], Jing Wang [1], Jicheng Liu [2] and Ming Bu [1,*]

1 College of Pharmacy, Qiqihar Medical University, Qiqihar 161006, China.

2 Research Institute of Medicine & Pharmacy, Qiqihar Medical University, Qiqihar 161006, China.

[*]E-mail address: buming@qmu.edu.cn (M. Bu).

^{1}H NMR and ^{13}C NMR of compound 2

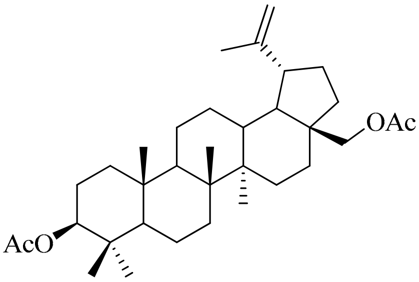

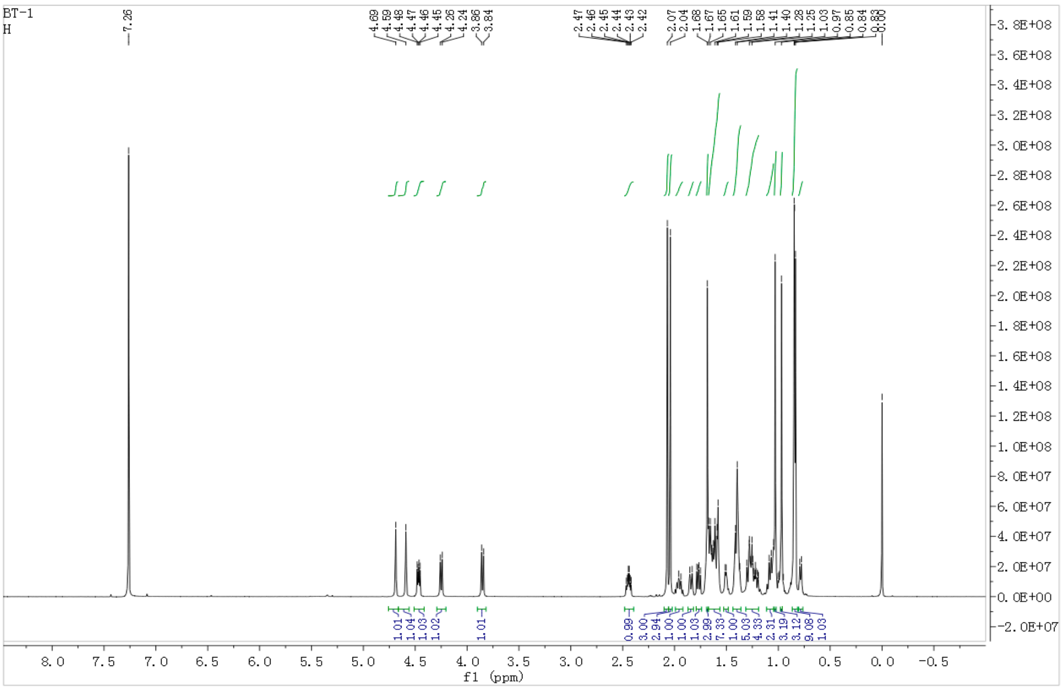

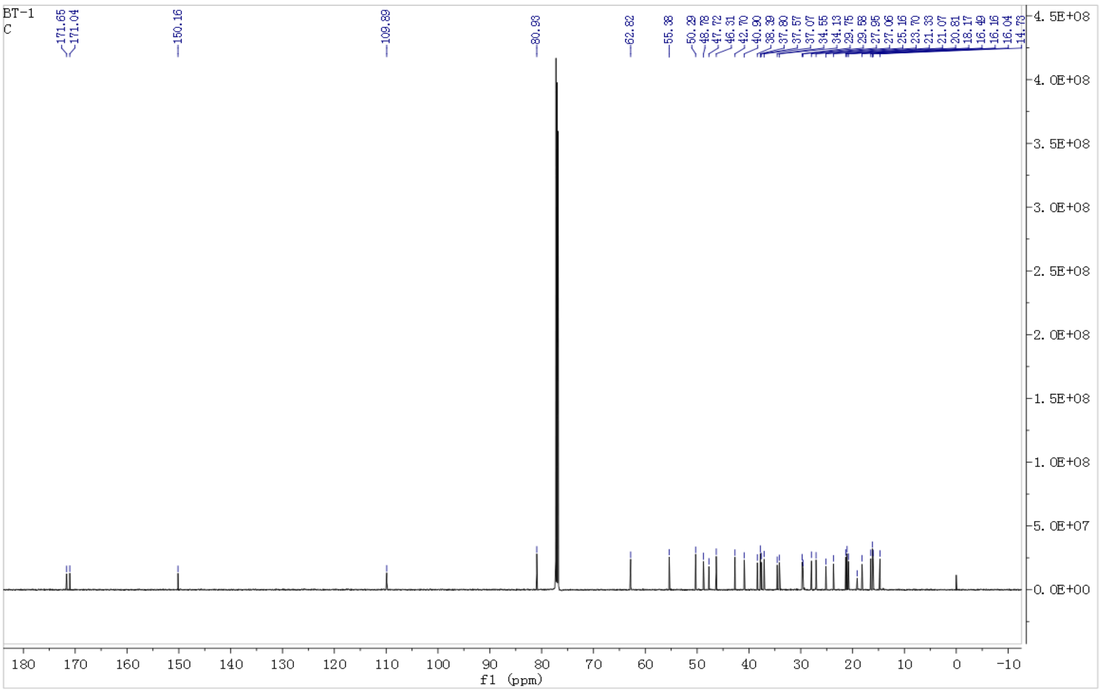

^{1}H NMR and ^{13}C NMR of compound 3

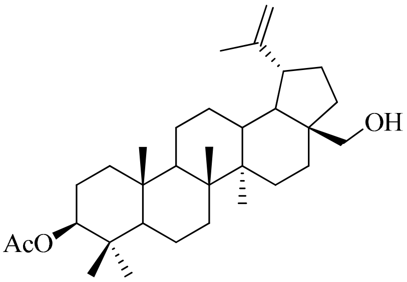

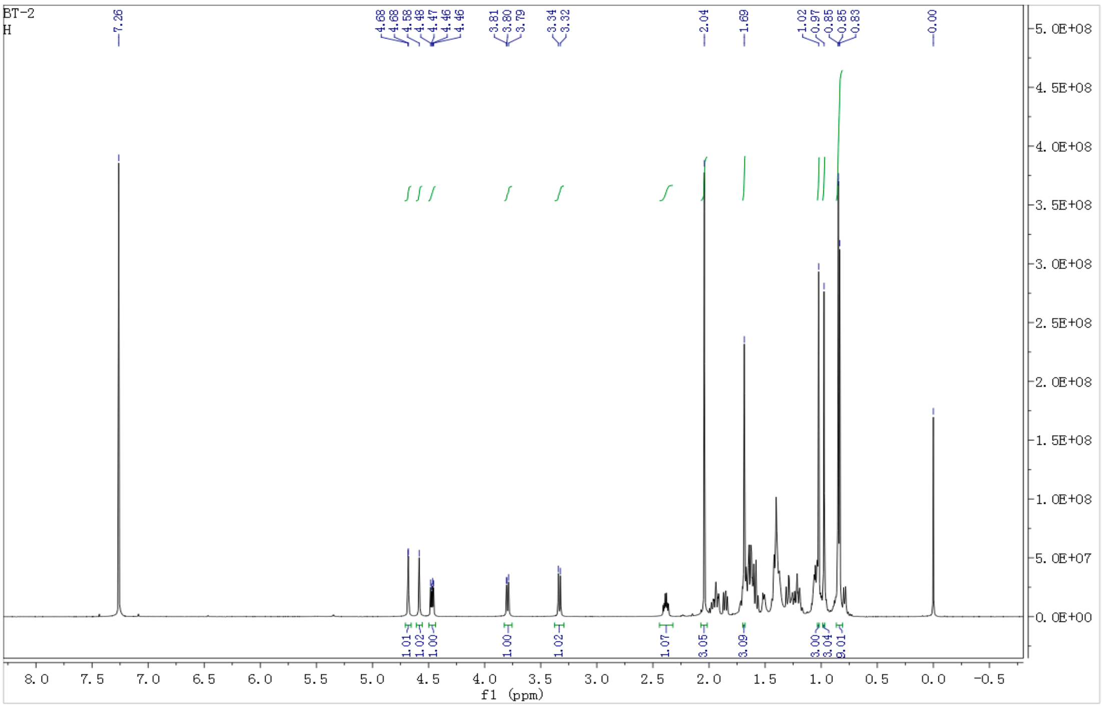

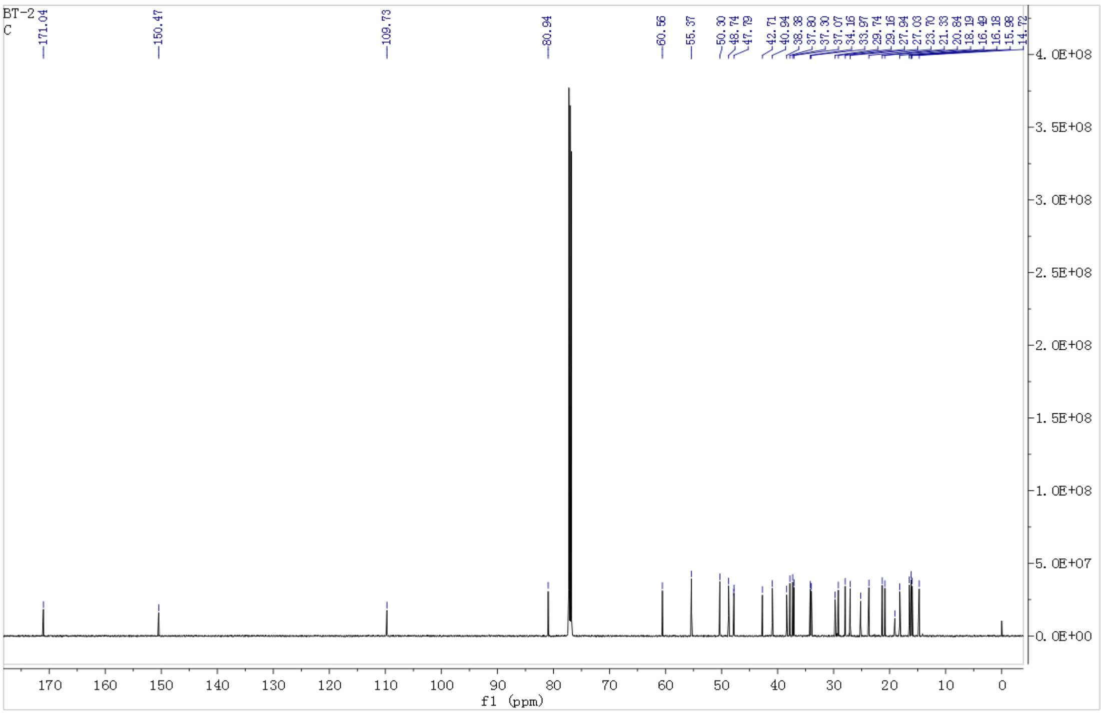

^{1}H NMR and ^{13}C NMR of compound 4

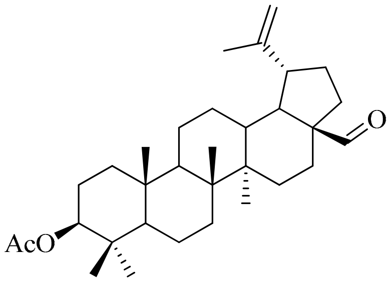

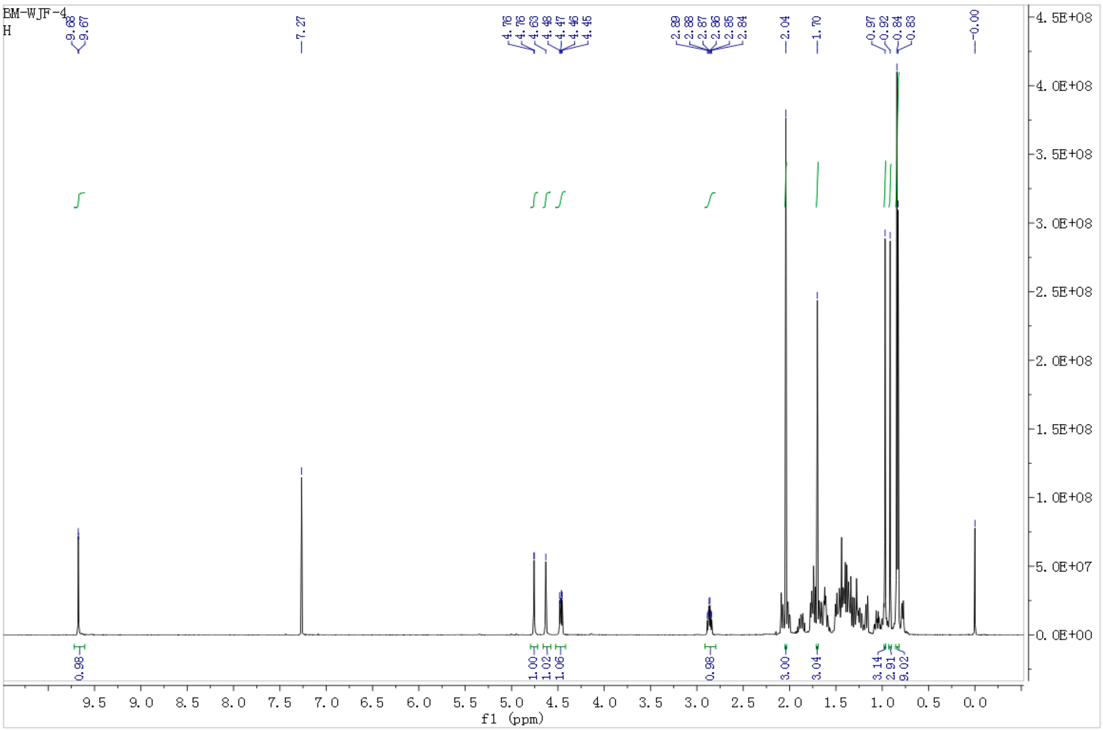

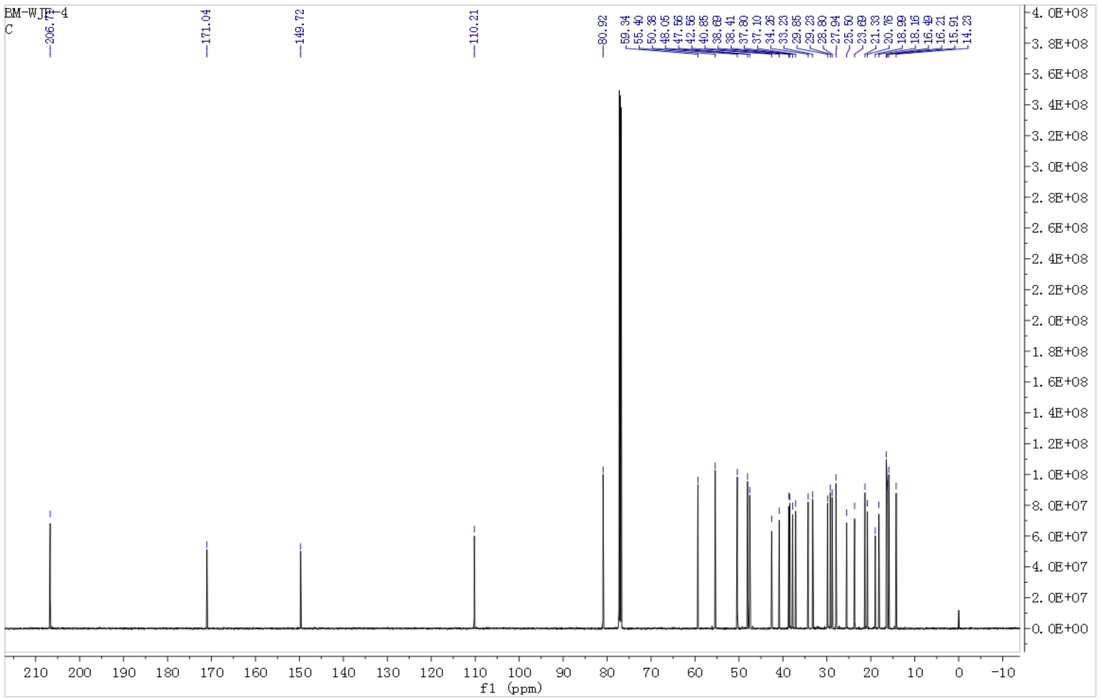

^{1}H NMR and ^{13}C NMR of compound 5

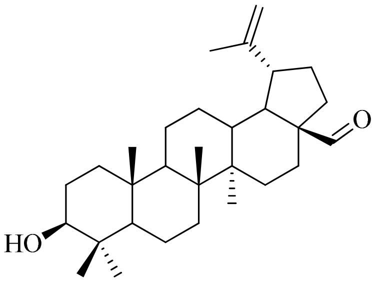

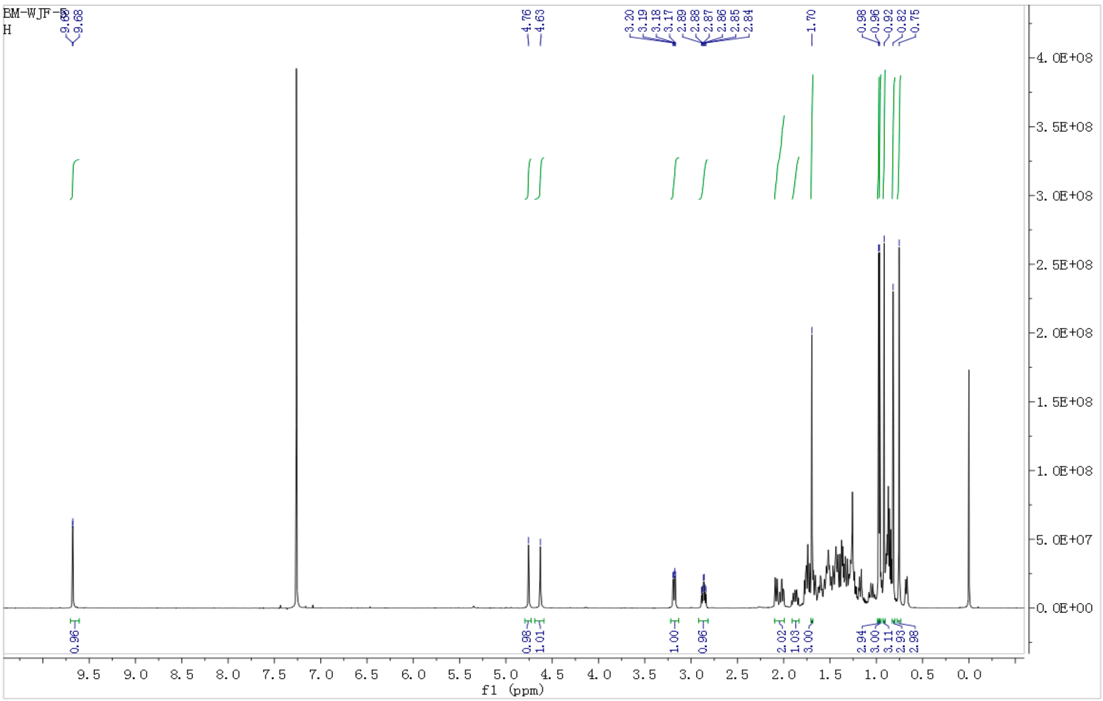

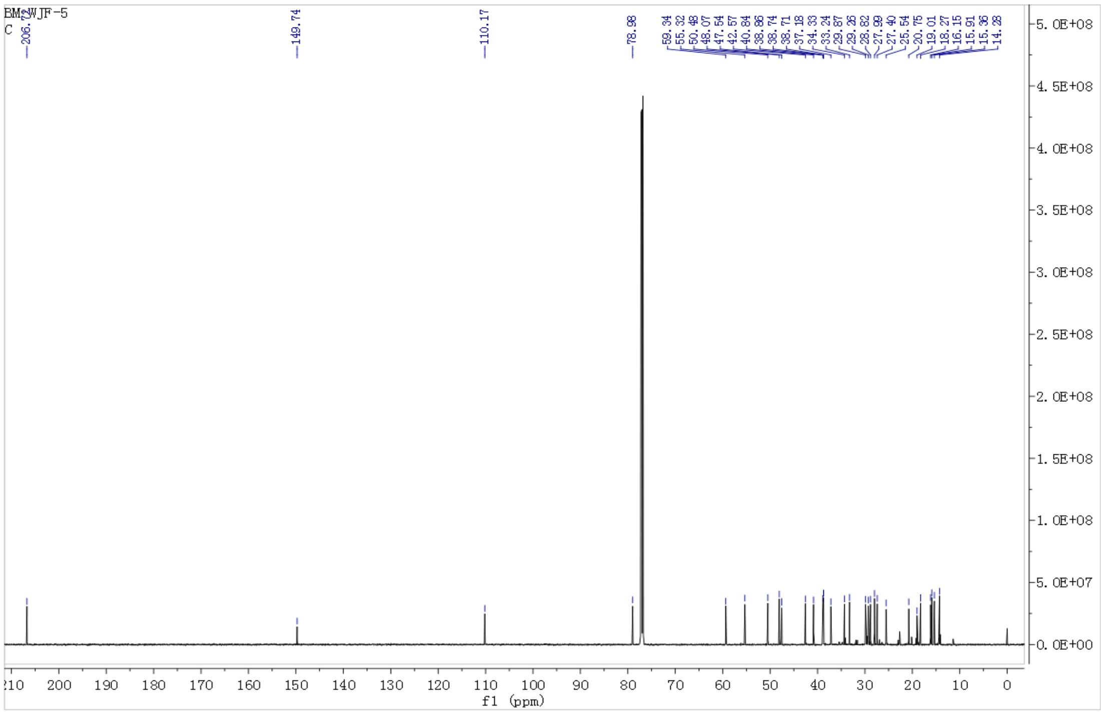

^{1}H NMR and ^{13}C NMR of compound 6

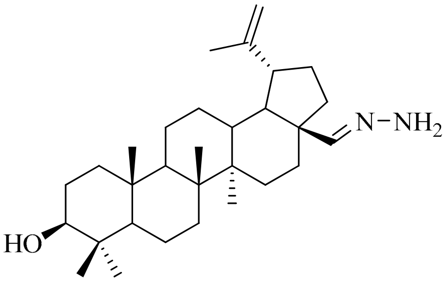

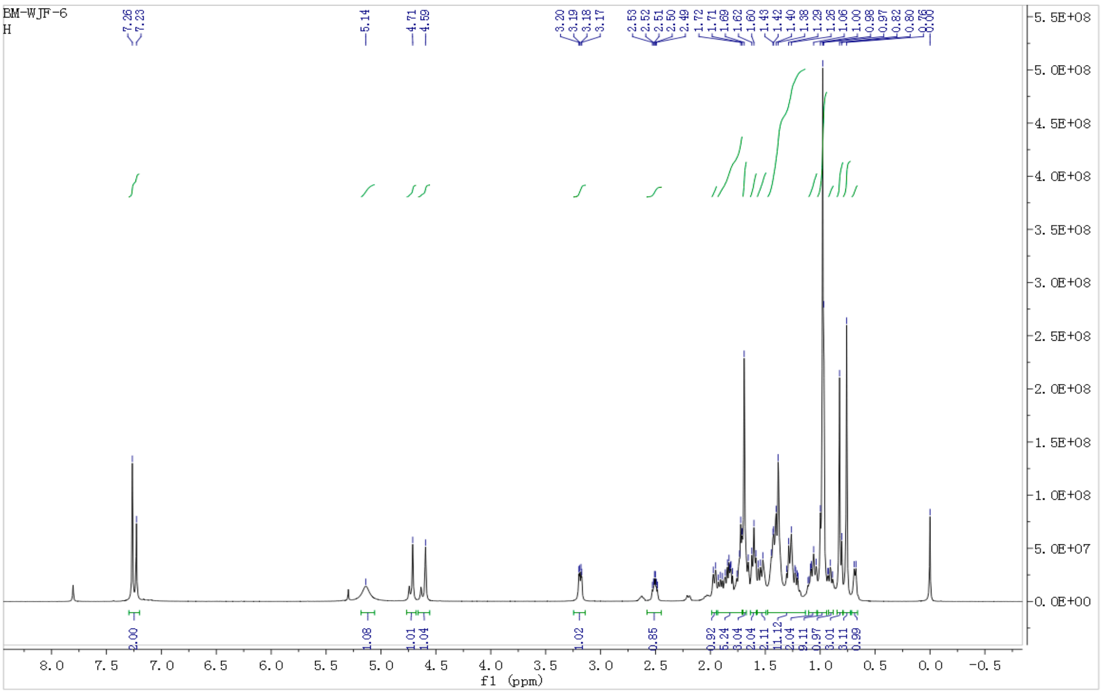

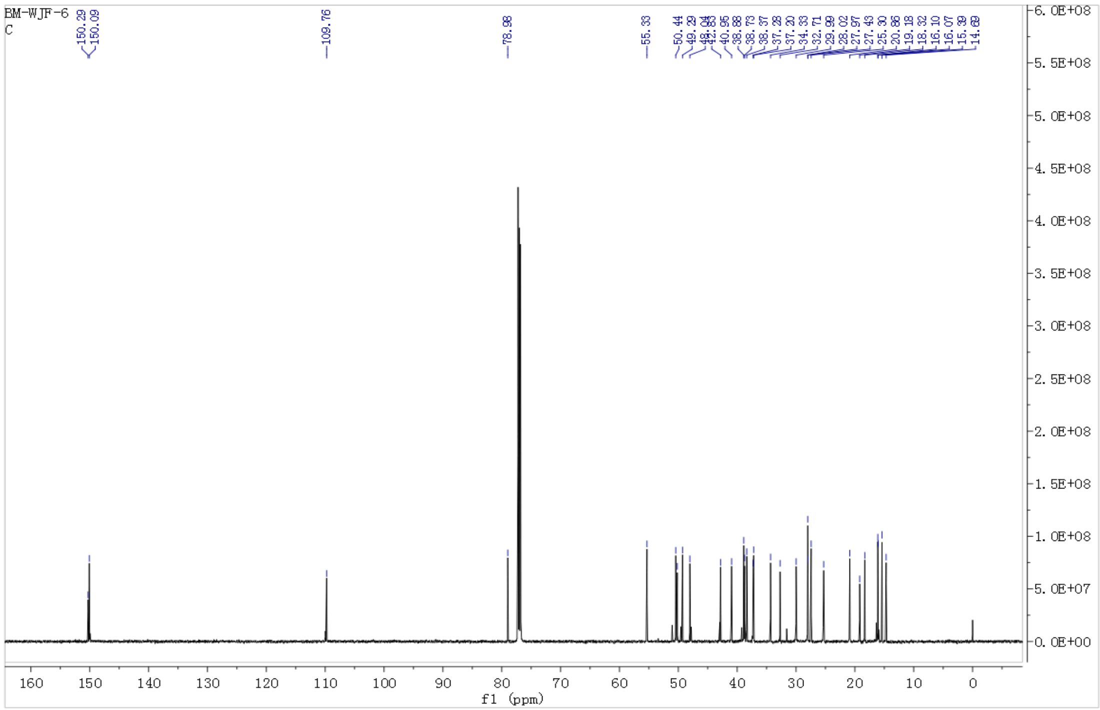

HRMS, ^{1}H NMR and ^{13}C NMR of compound 7a

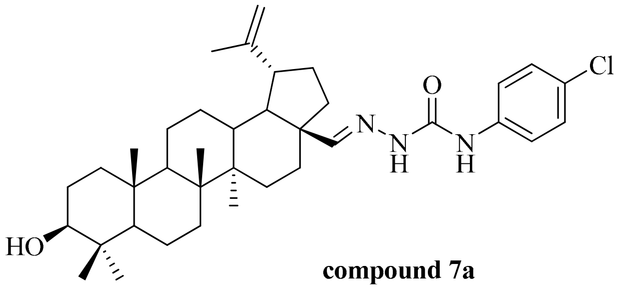

compound 7a

C37H54ClN3O2

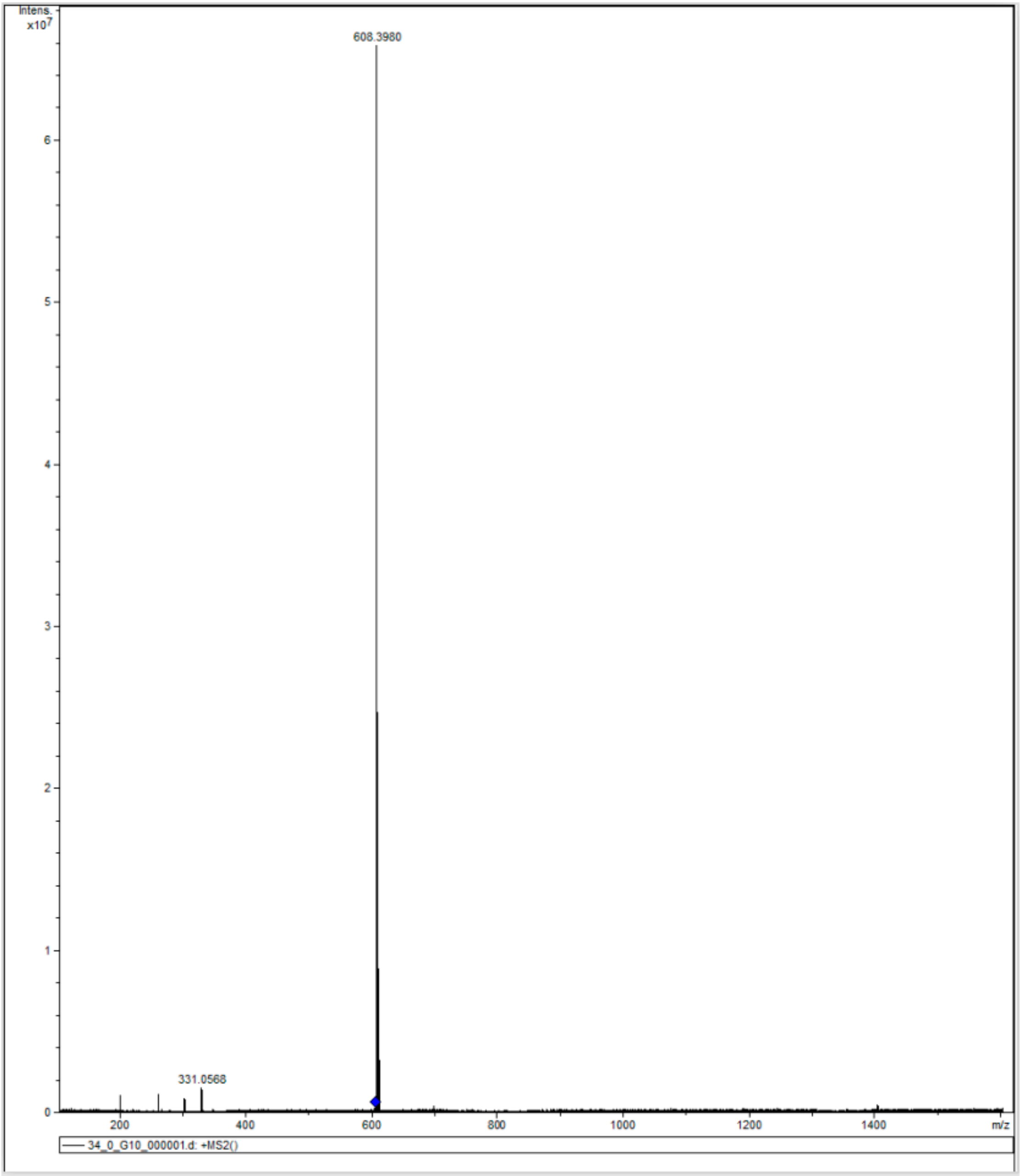

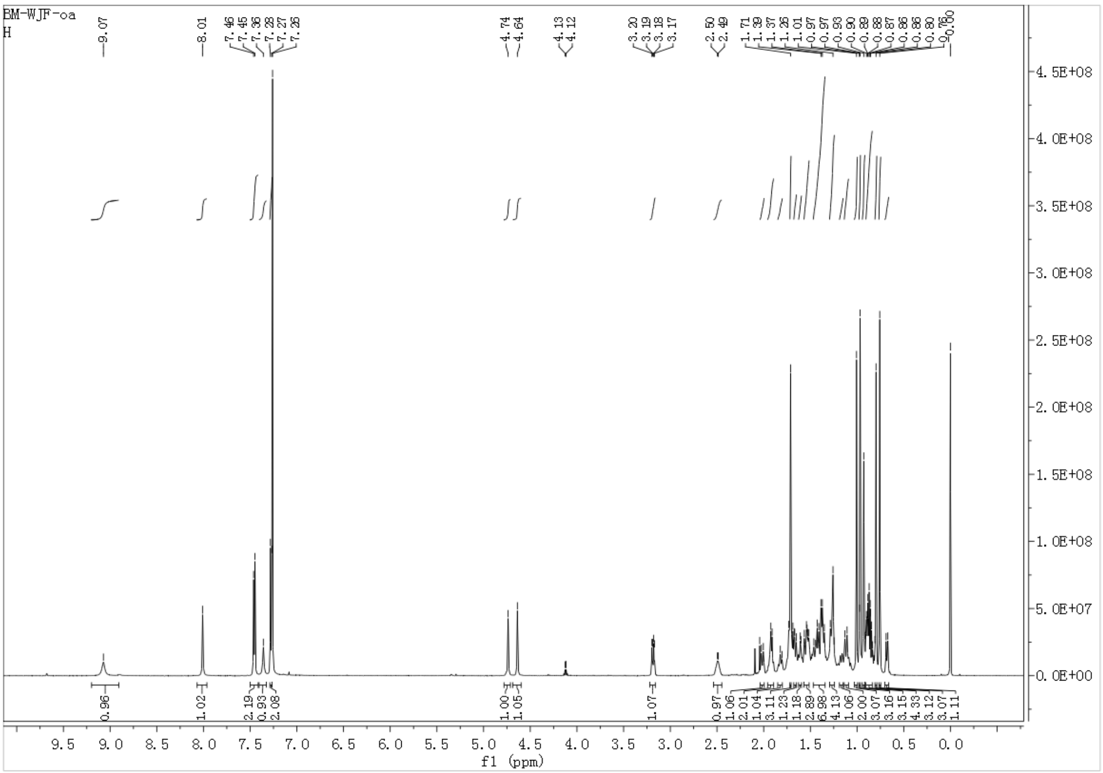
BM-WJF-oa
H
f1 (ppm)

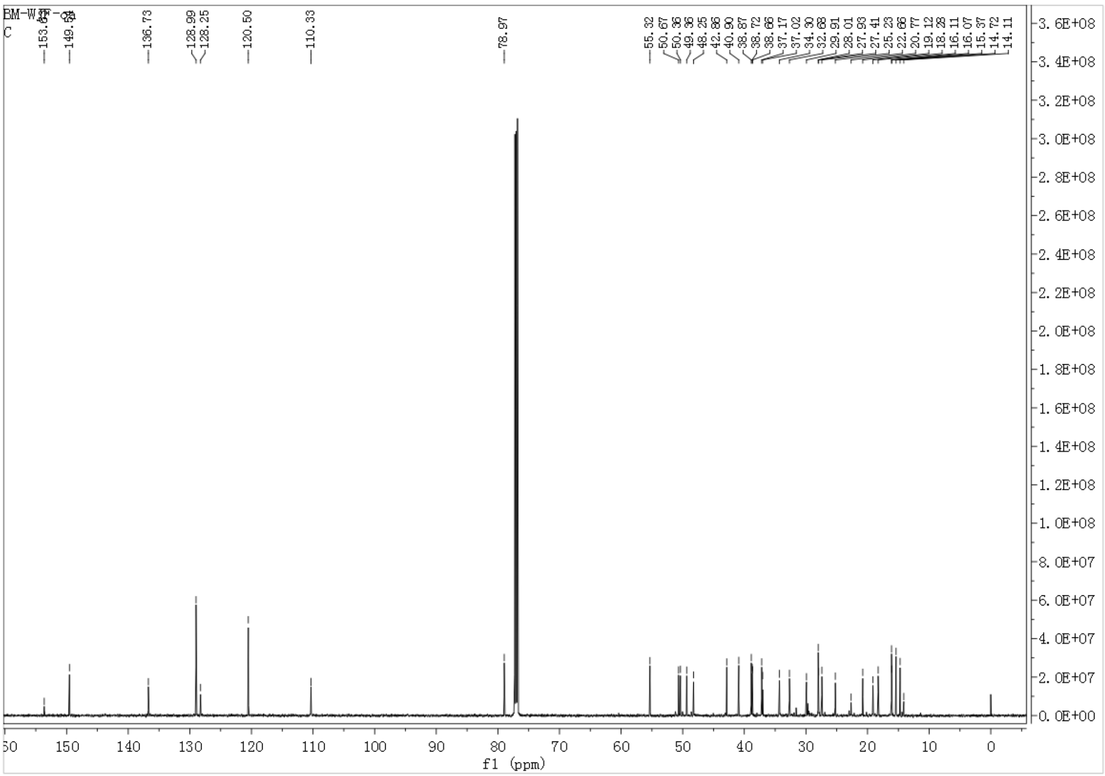
BM-WJF-oa
C
f1 (ppm)

HRMS, ^{1}H NMR and ^{13}C NMR of compound 7b

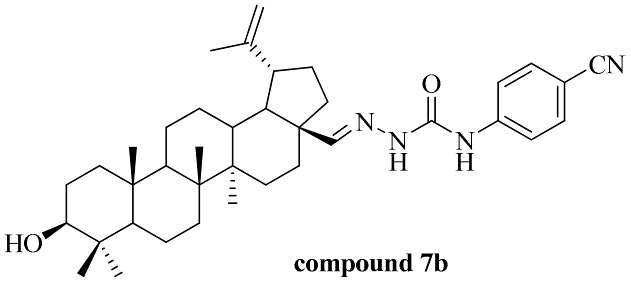

compound 7b

C38H54N4O2

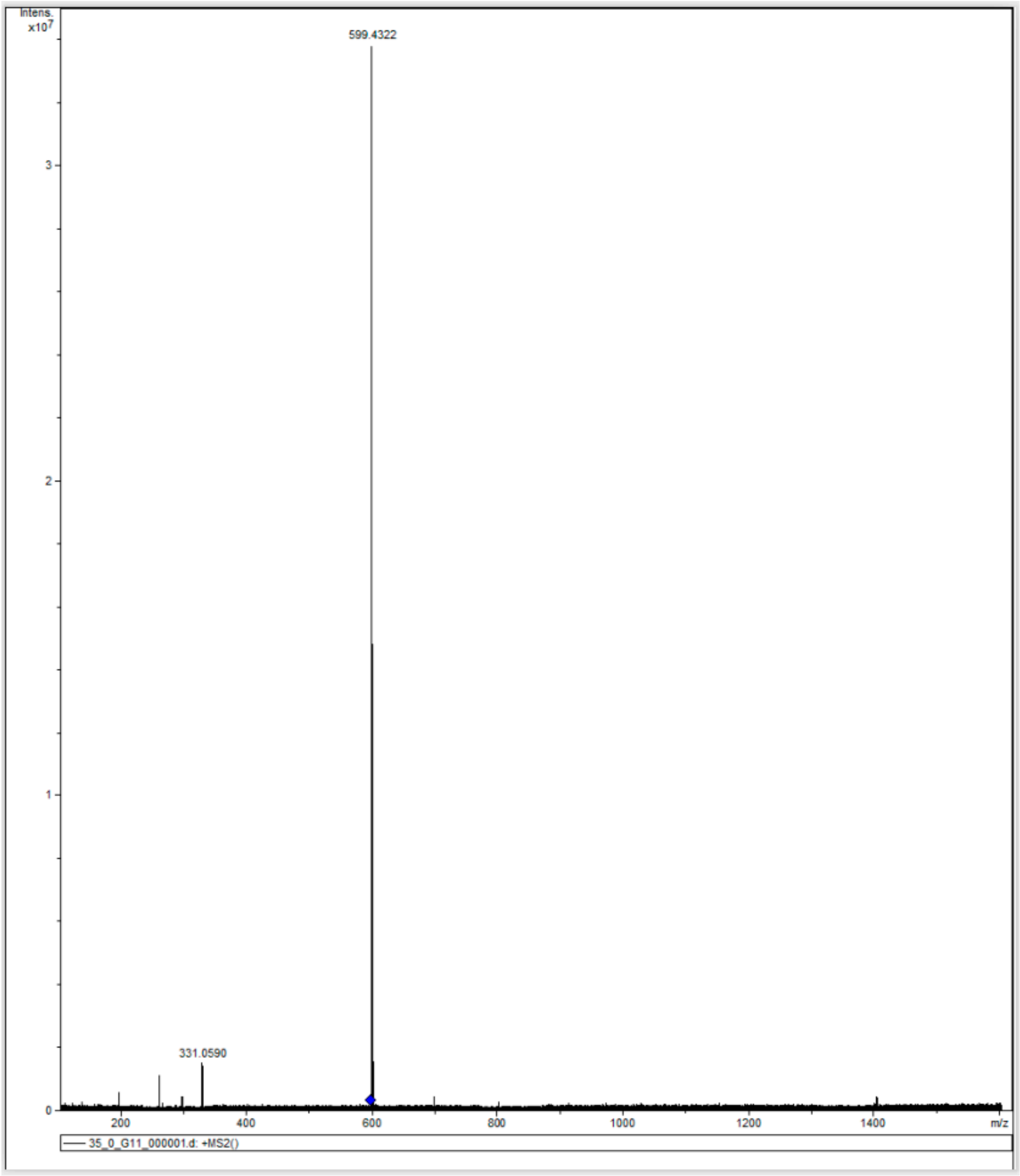

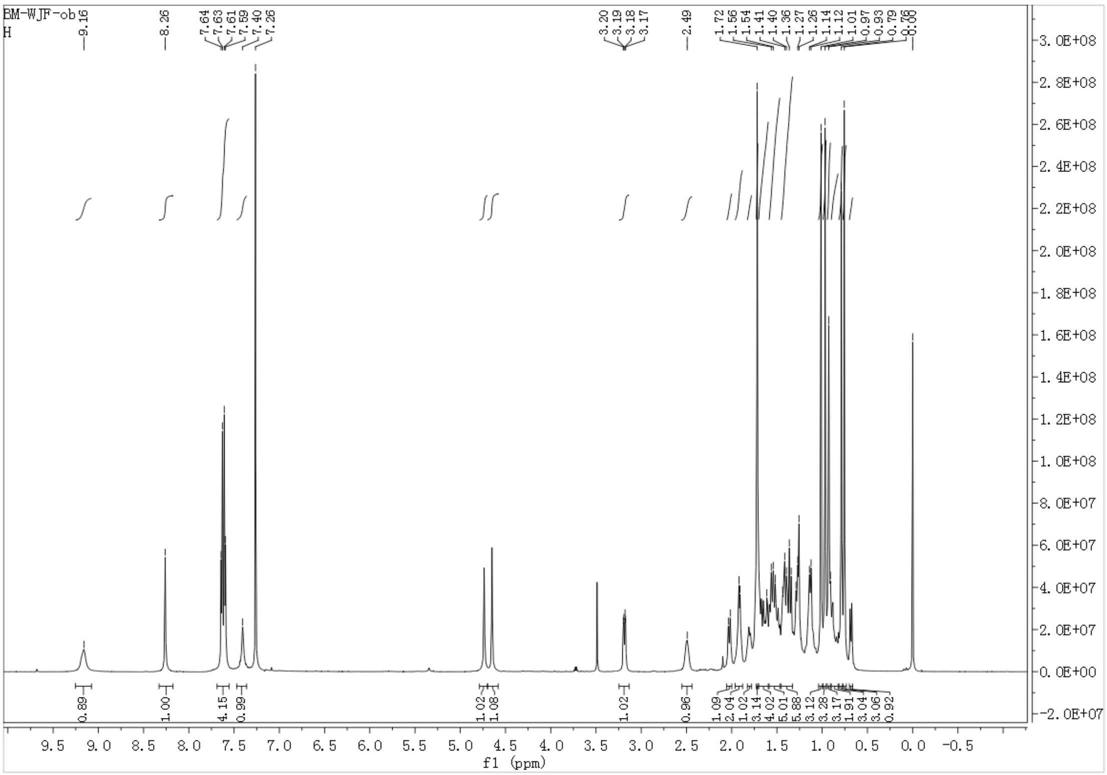
BM-WJF-ob
H
f1 (ppm)

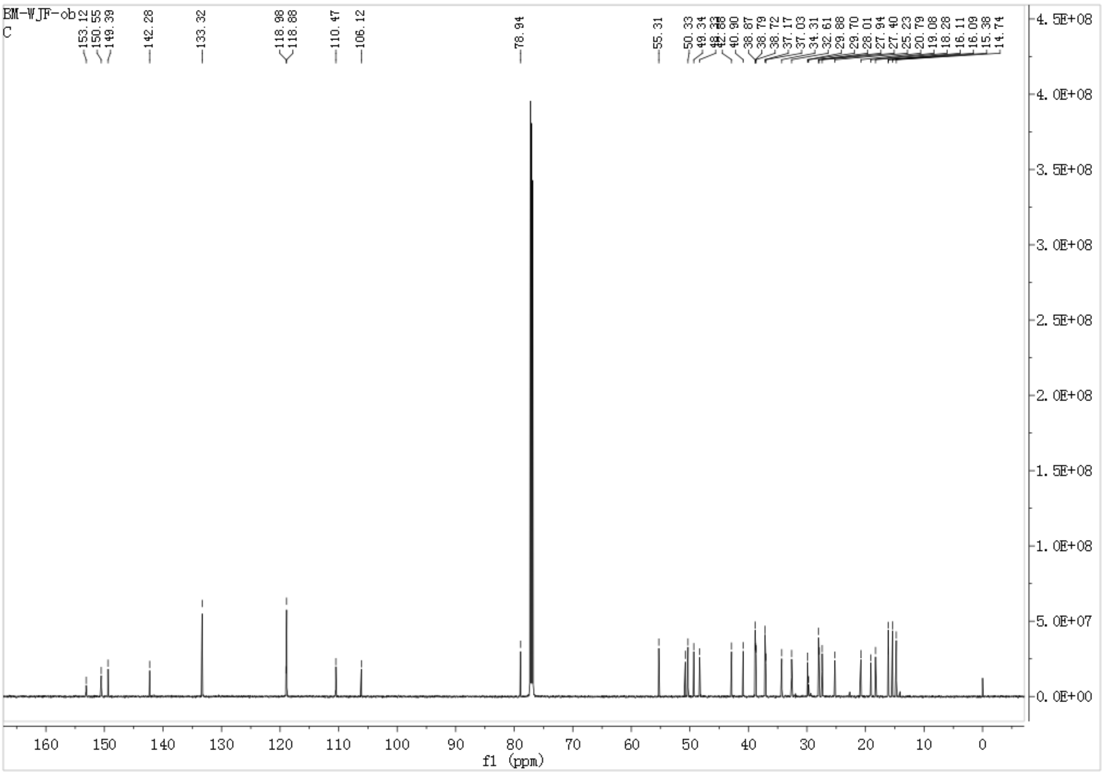
BM-WJF-ob
C
f1 (ppm)

HRMS, ^{1}H NMR and ^{13}C NMR of compound 7c

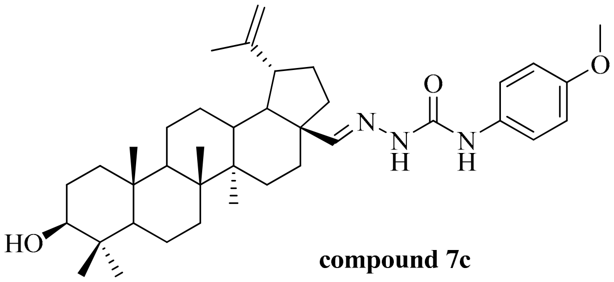

compound 7c

C38H57N3O3

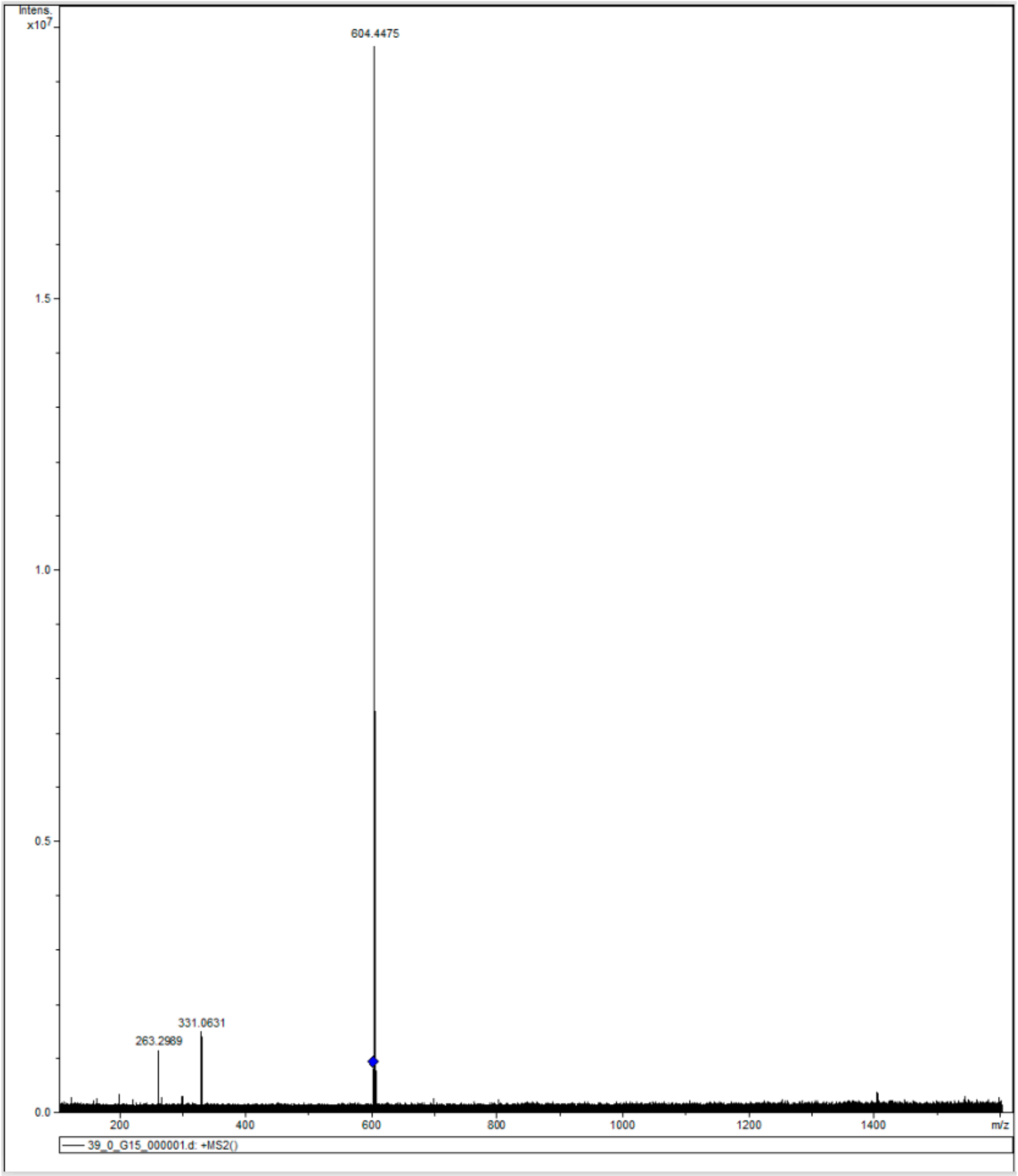

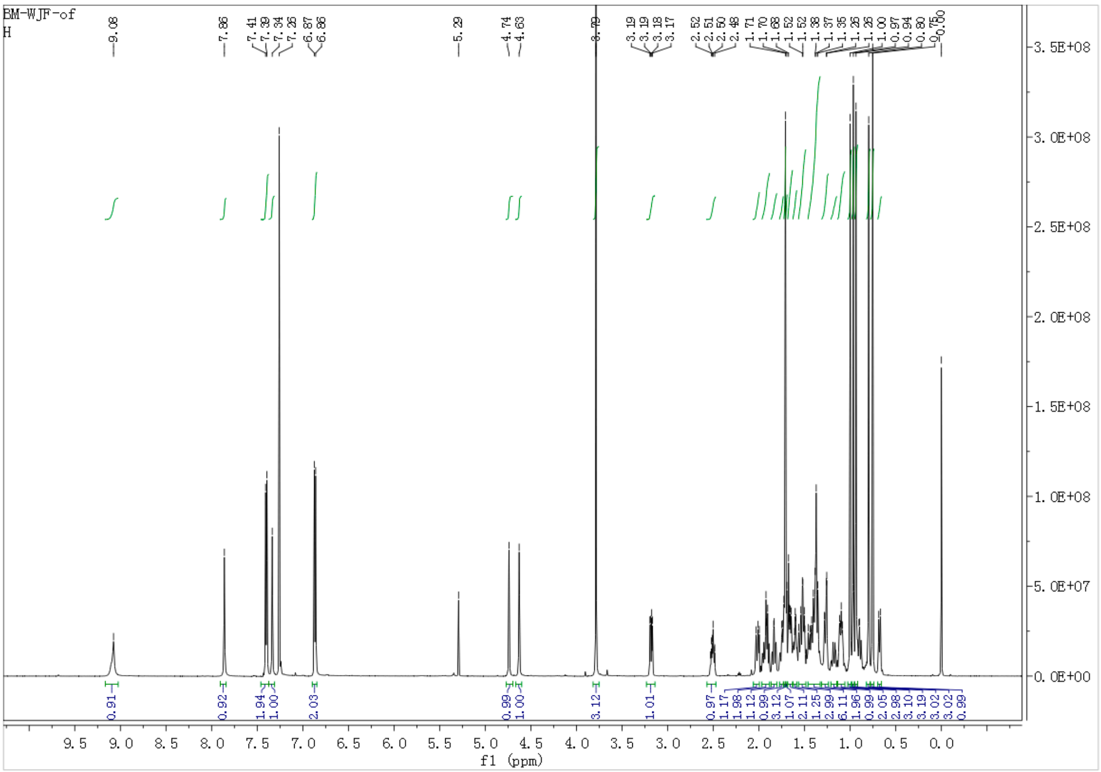
BM-WJF-of
H
f1 (ppm)

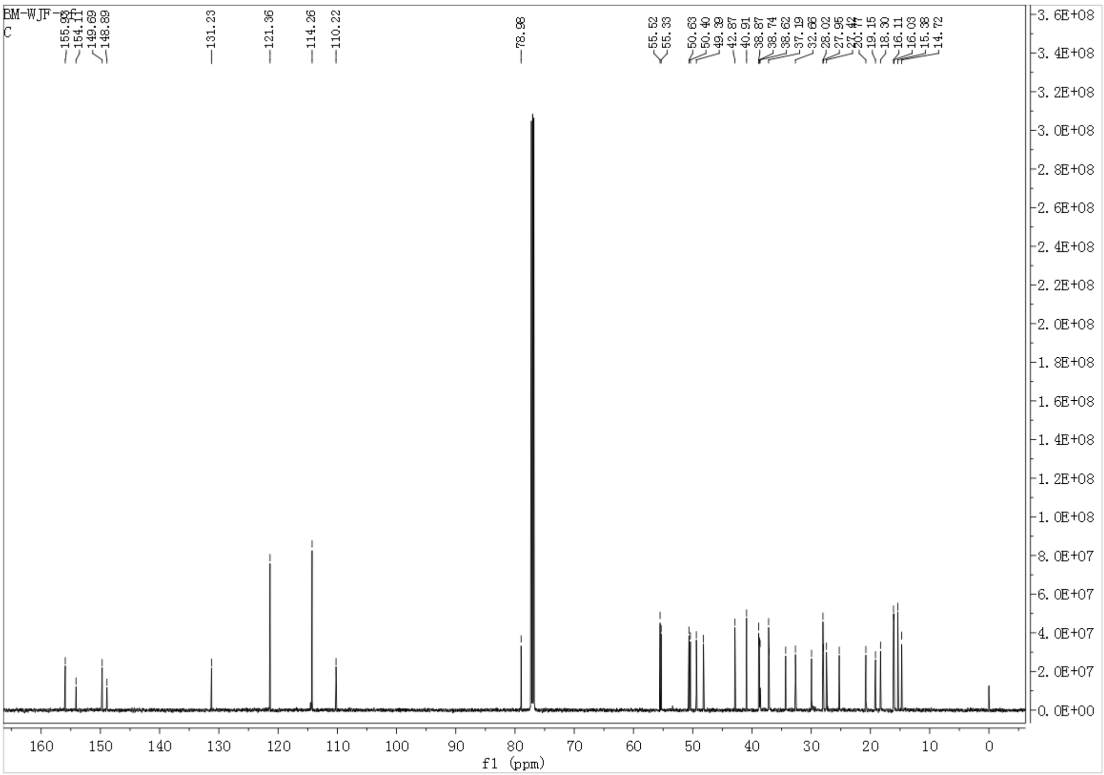
C
f1 (ppm)

HRMS, ^{1}H NMR and ^{13}C NMR of compound 7d

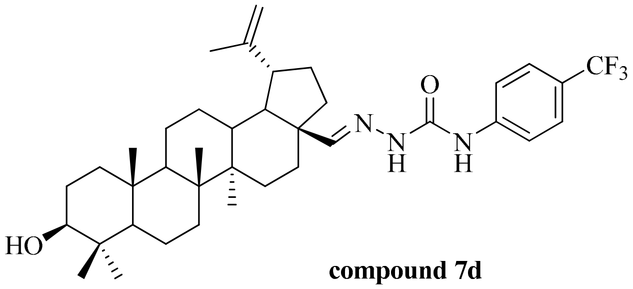

compound 7d

C38H54F3N3O2

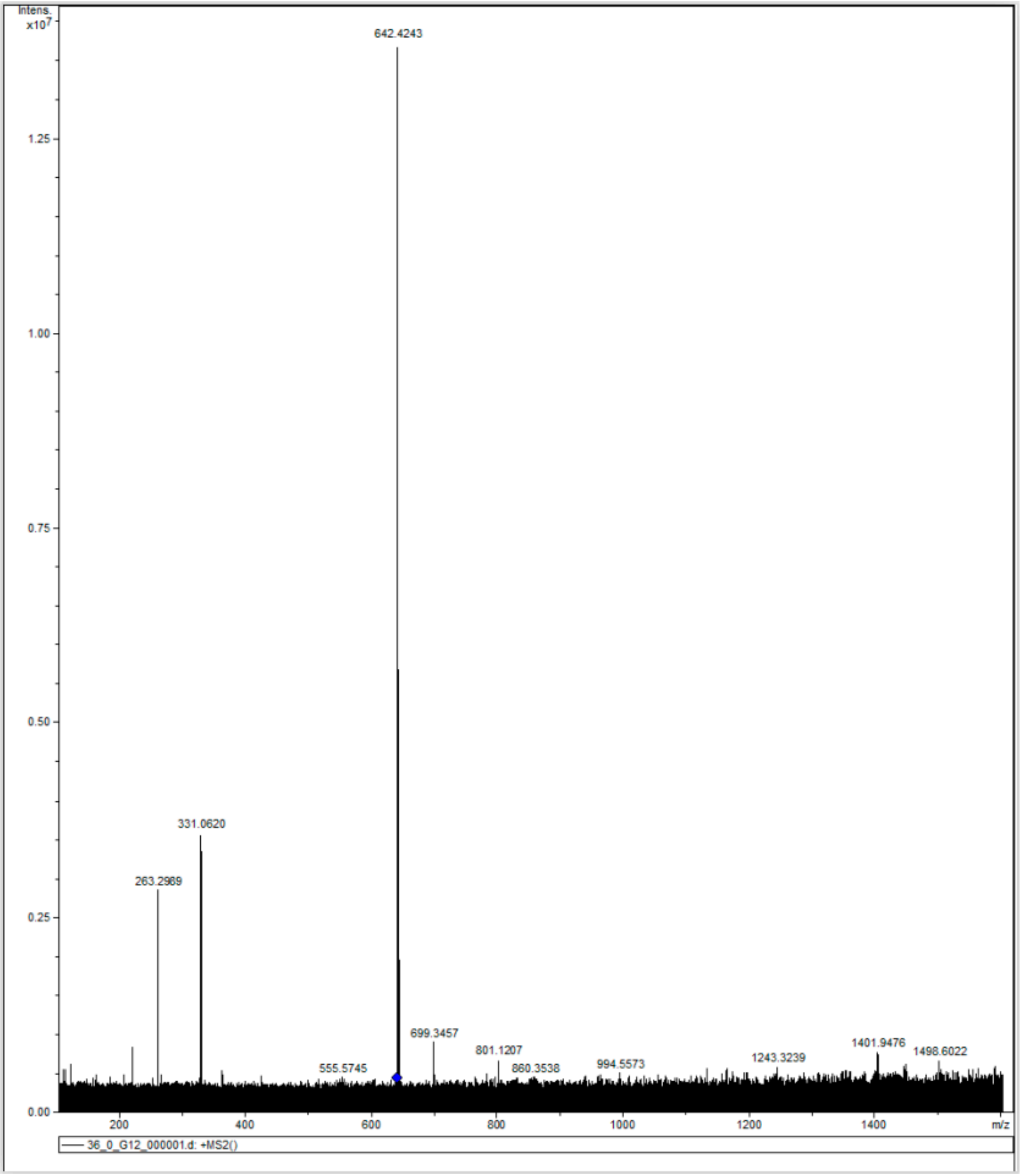

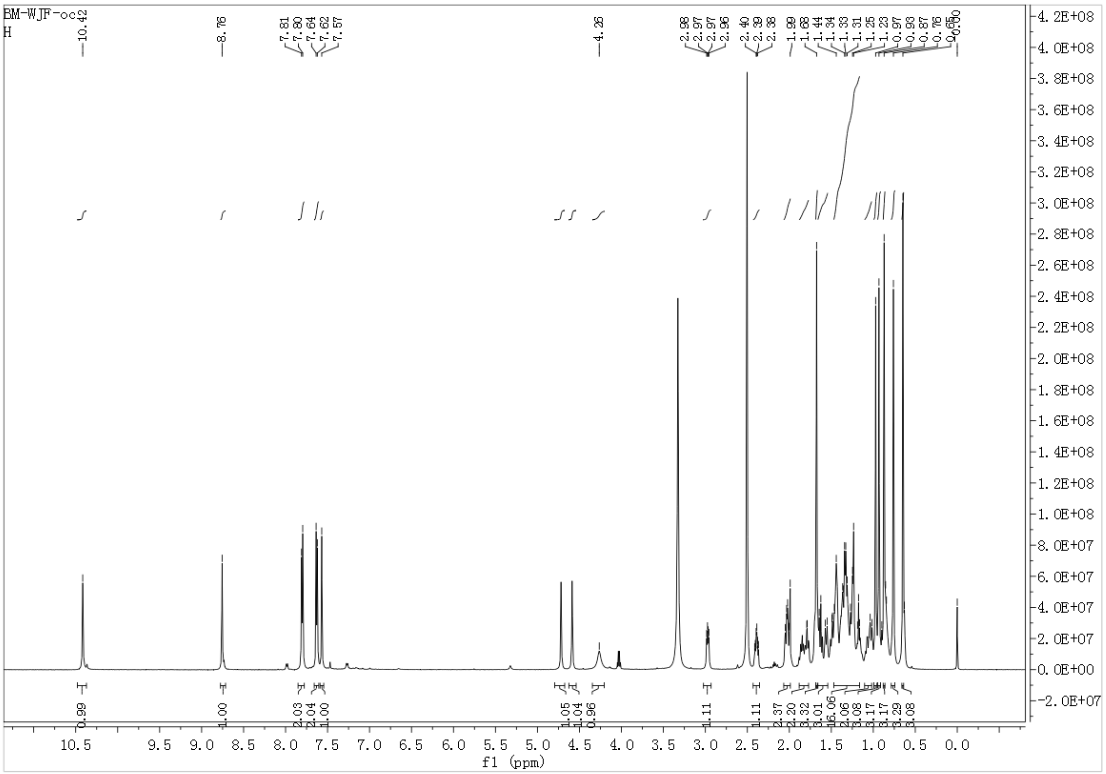

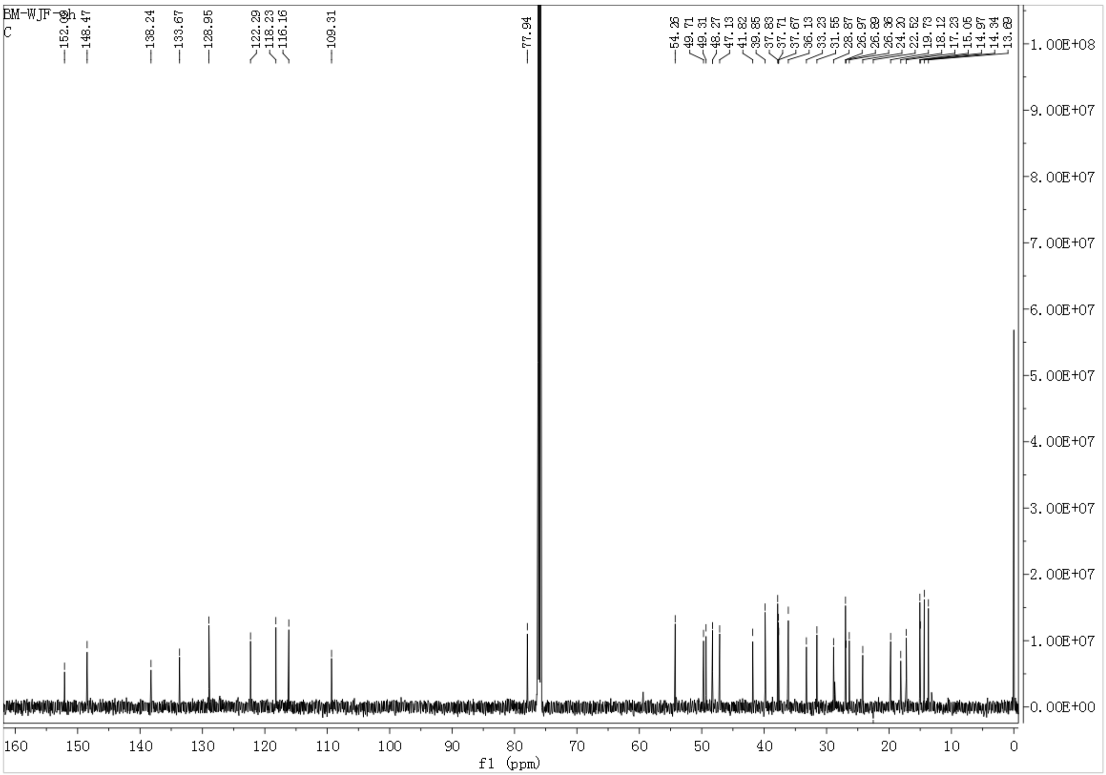

HRMS, ^{1}H NMR and ^{13}C NMR of compound 7e

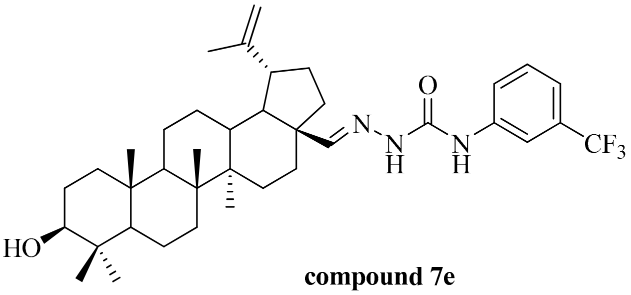

compound 7e

C38H54F3N3O2

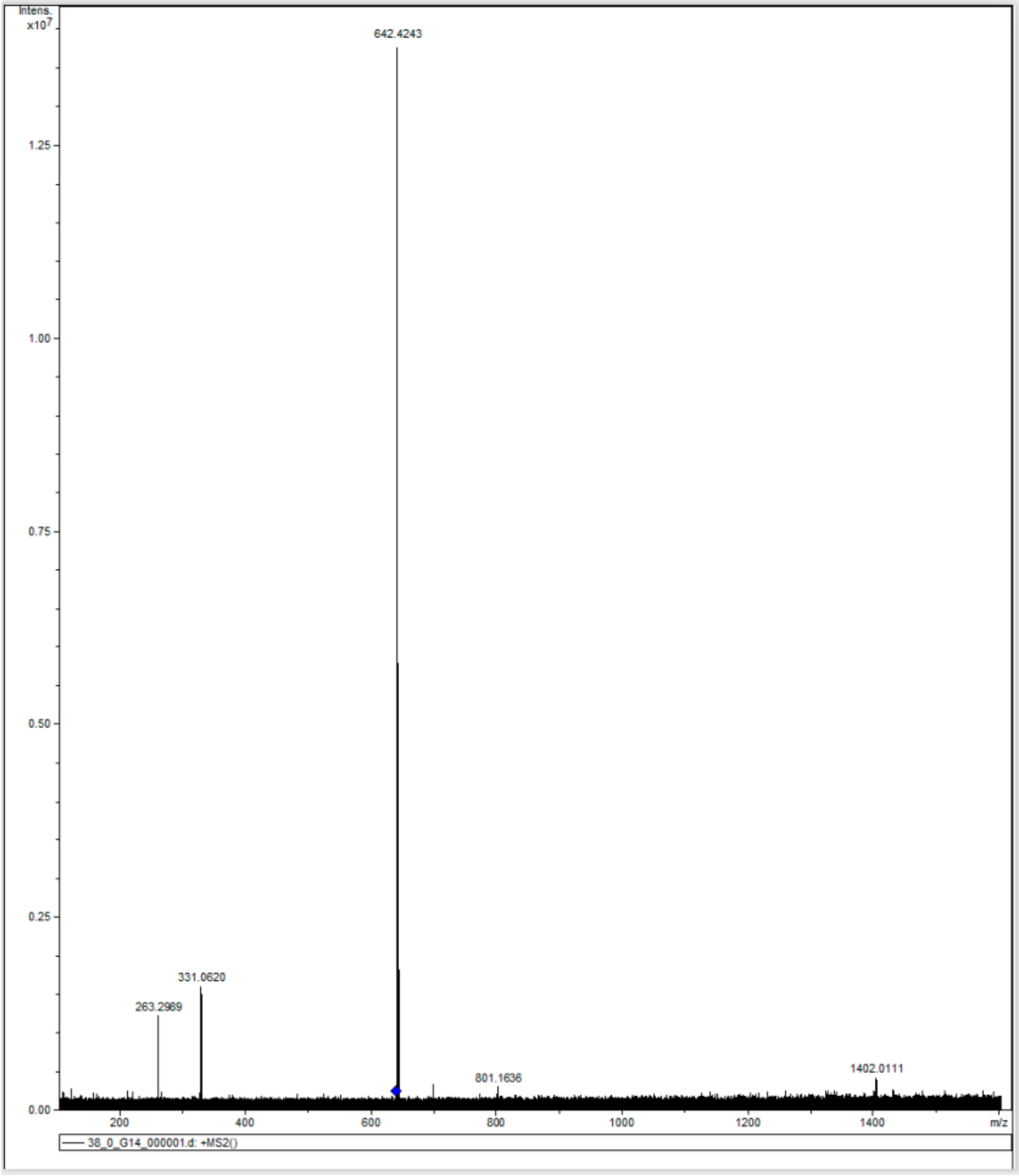

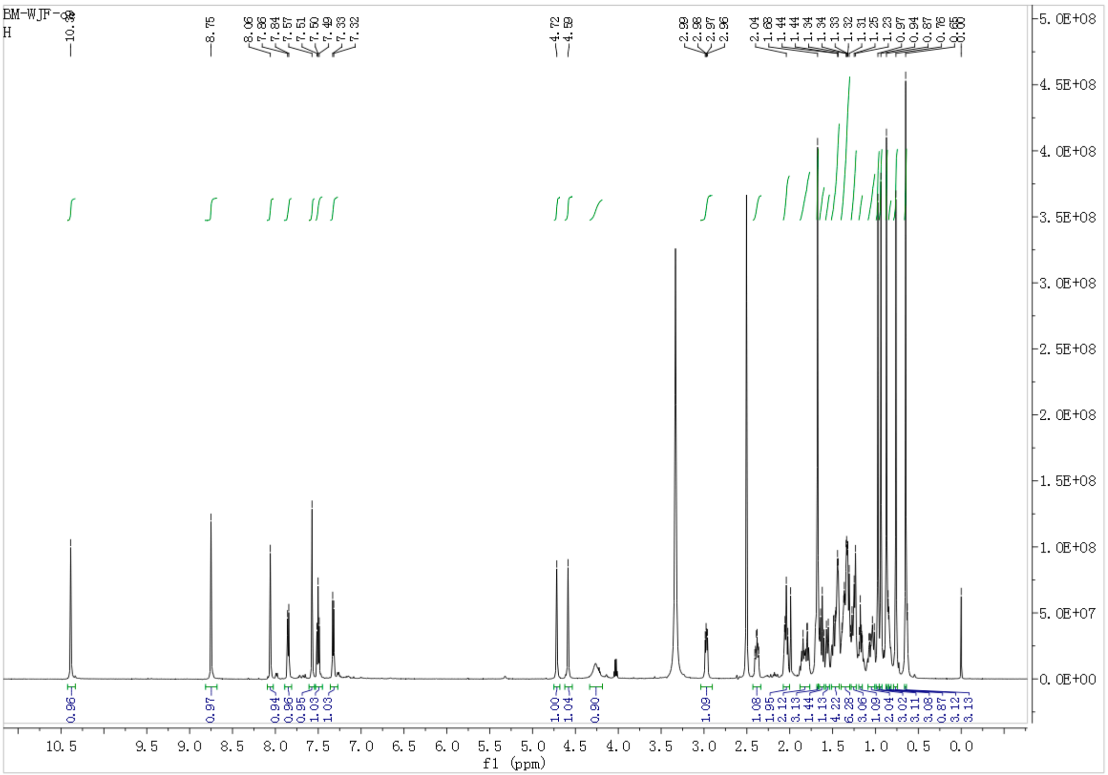
H
10.39
8.75
8.06 7.86 7.84 7.57 7.51 7.50 7.49 7.33 7.32
4.72 4.59
2.99 2.98 2.97 2.96
2.04 1.68 1.44 1.44 1.34 1.34 1.33 1.32 1.31 1.25 1.23 0.97 0.94 0.87 0.76 0.65 0.00
0.96 0.97 0.94 0.96 0.95 1.03 1.03 1.00 1.04 0.90 1.09 1.08 1.95 2.12 3.13 1.44 1.13 4.22 6.28 3.06 1.09 2.04 3.02 3.11 3.08 0.87 3.12 3.13
f1 (ppm)

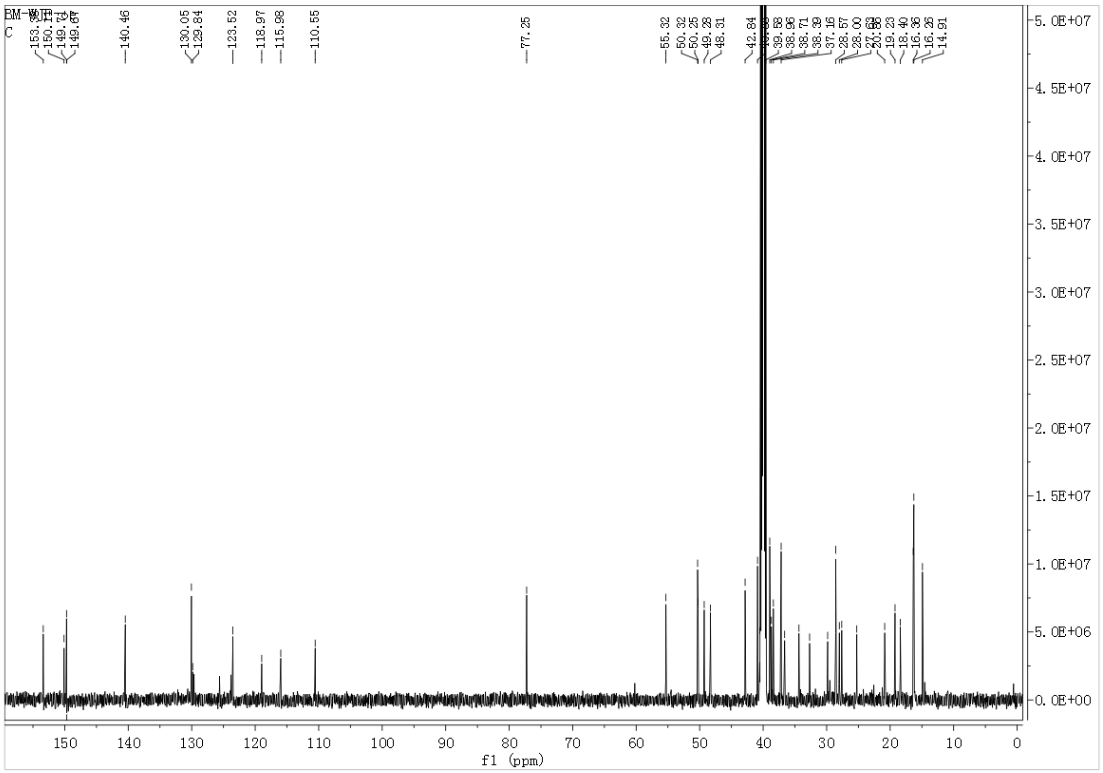
C
153.39 150.11 149.71 149.67
140.46
130.05 129.84
123.52
118.97
115.98
110.55
77.25
55.32 50.32 50.25 49.28 48.31
42.84 39.58 38.96 38.71 38.39 37.16 28.57 28.00 19.23 18.40 16.36 16.26 14.91
f1 (ppm)

HRMS, ^{1}H NMR and ^{13}C NMR of compound 7f

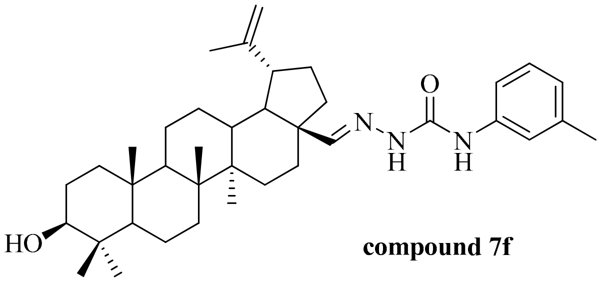

compound 7f

C38H57N3O2

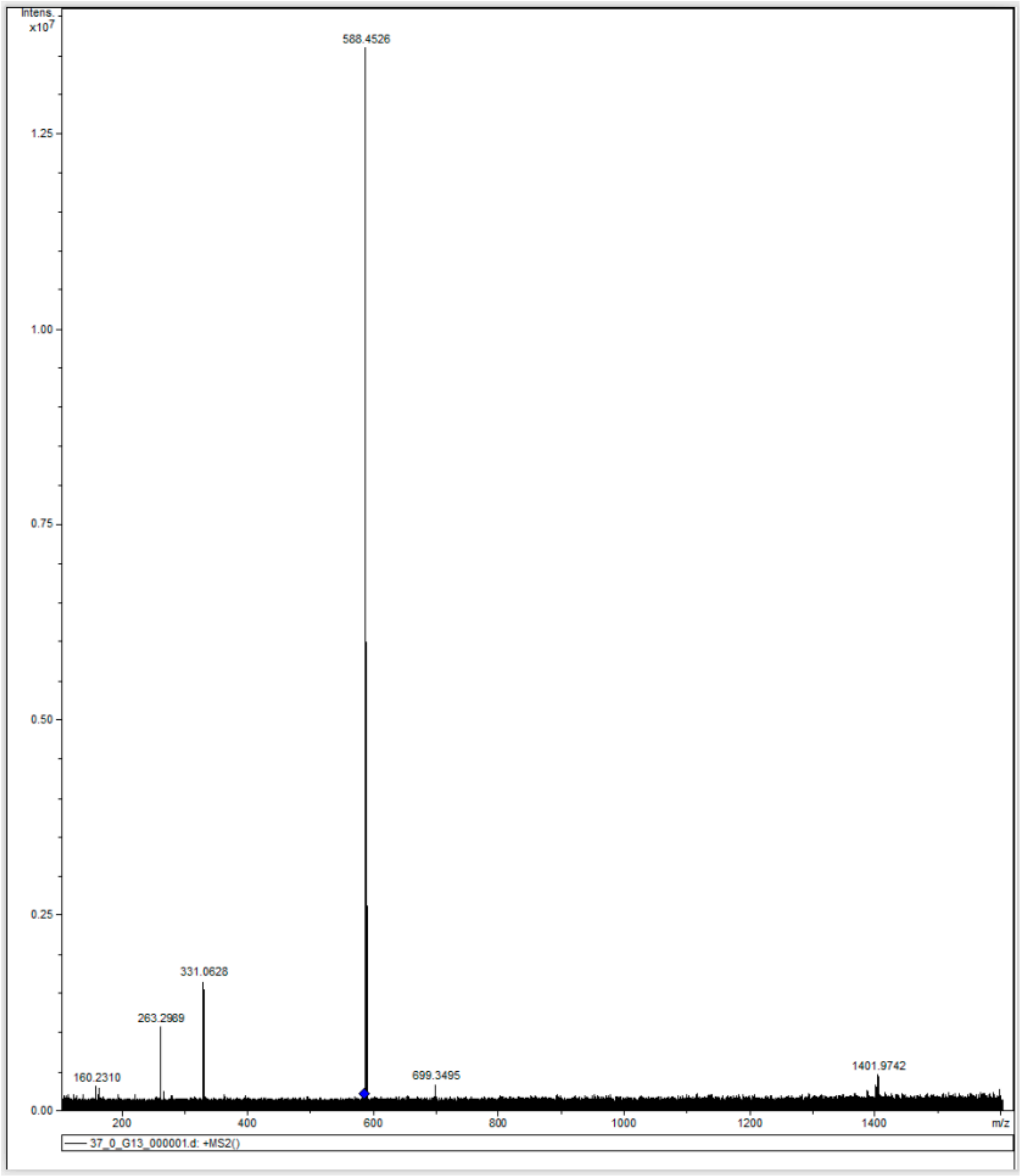

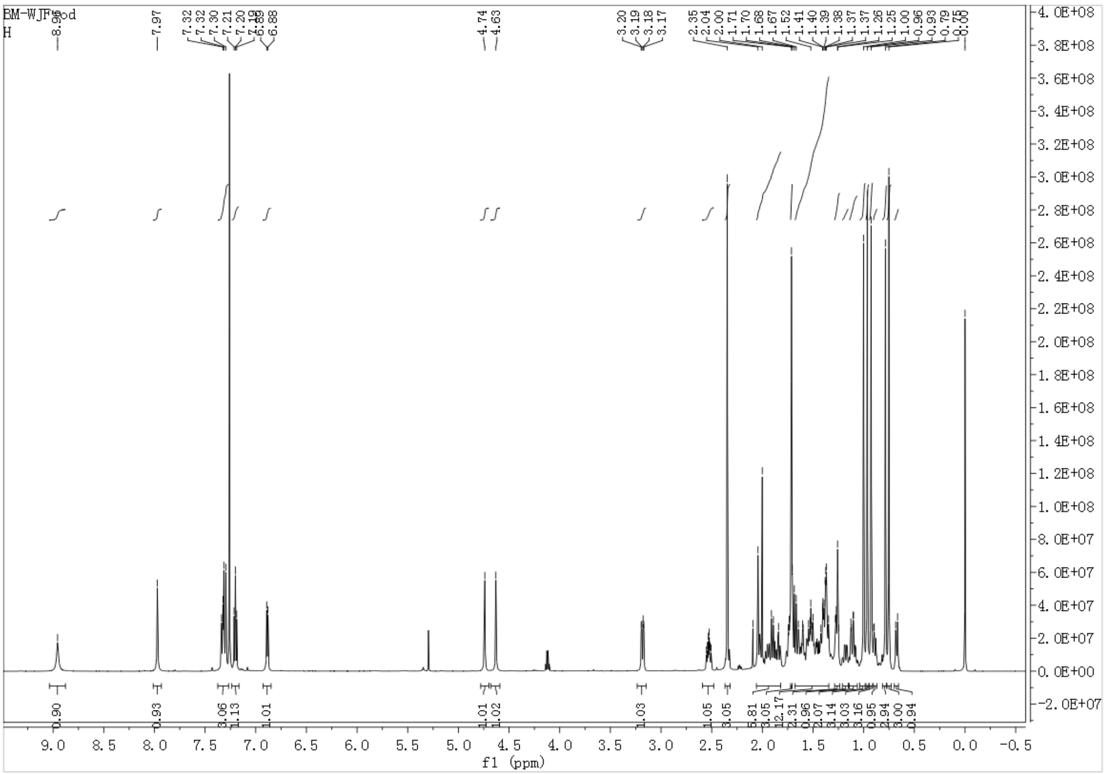
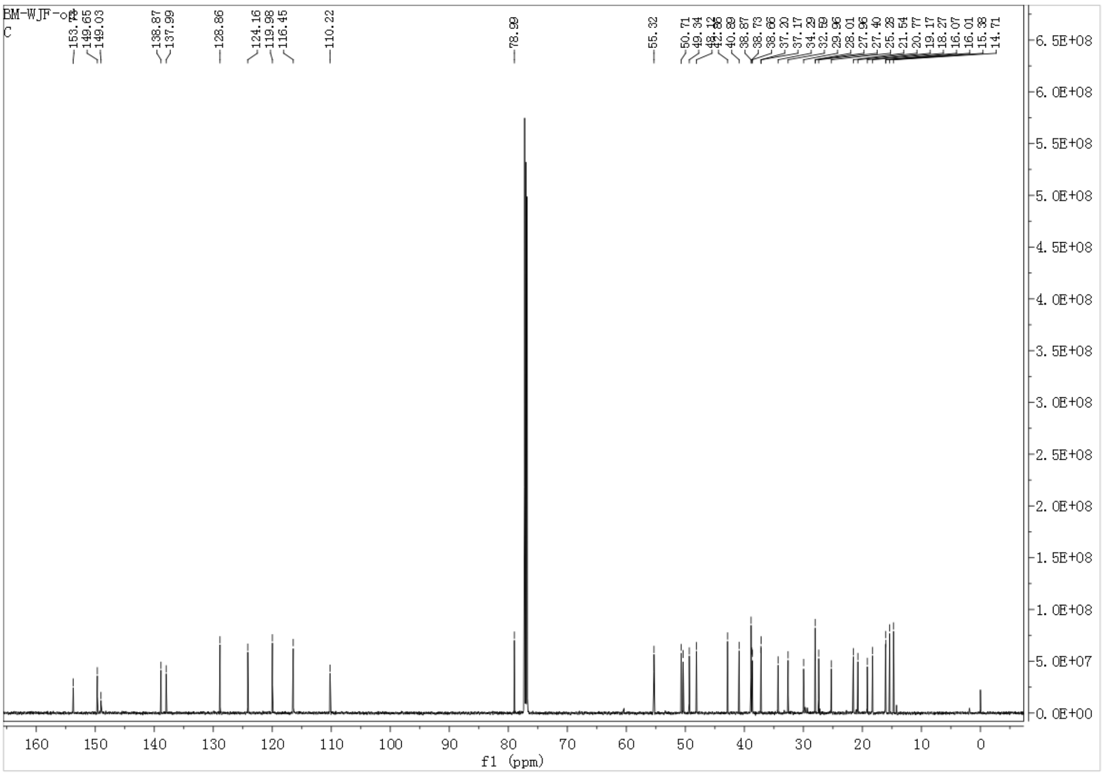

HRMS, ^{1}H NMR and ^{13}C NMR of compound 7g

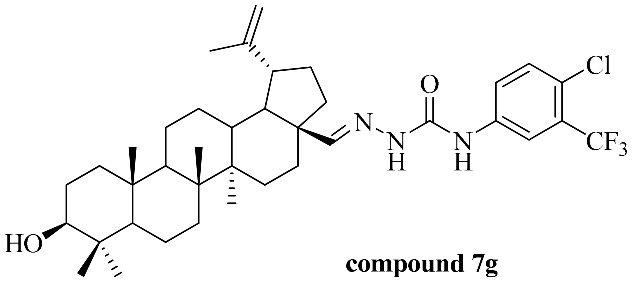

compound 7g

C38H53ClF3N3O2

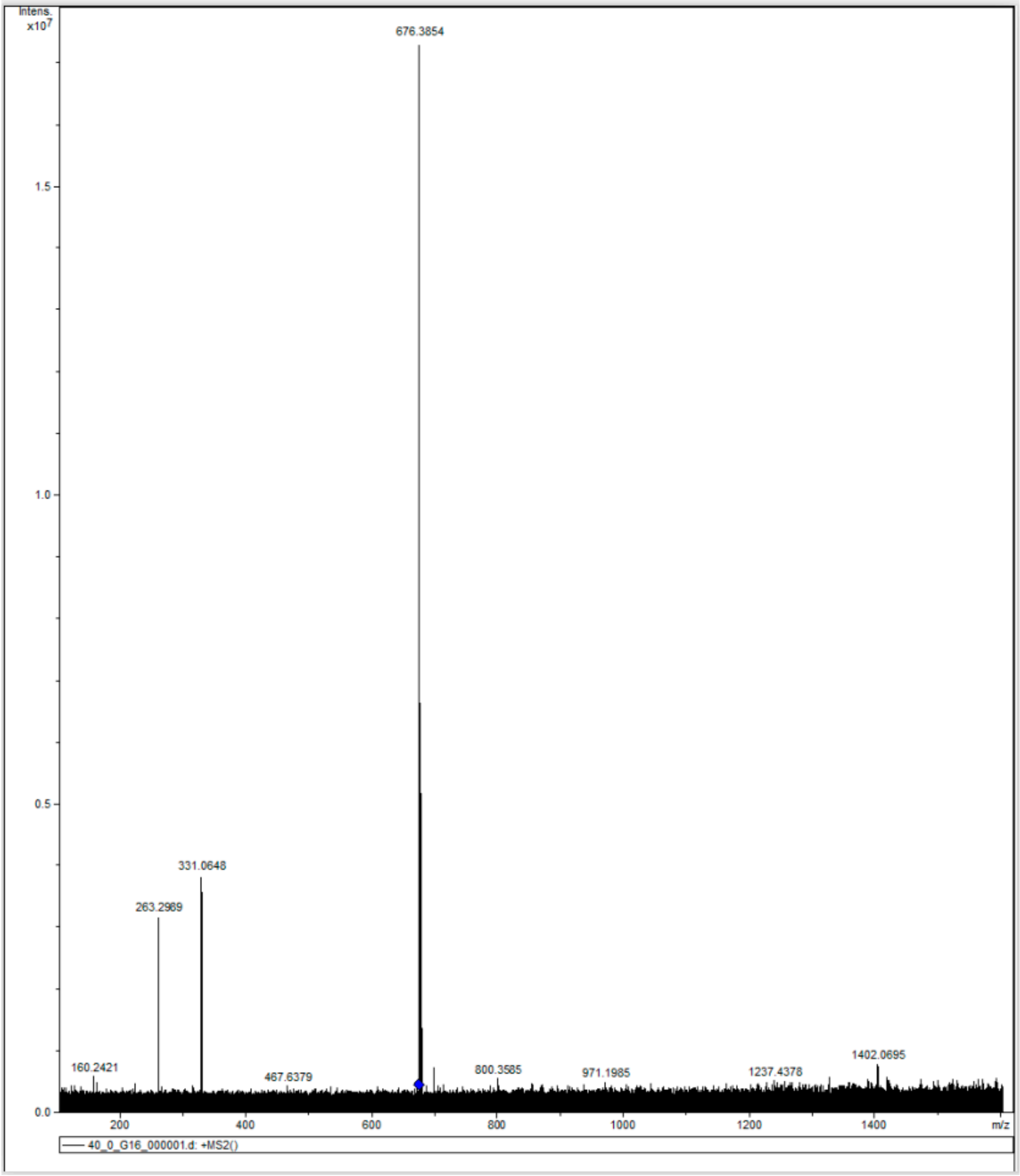

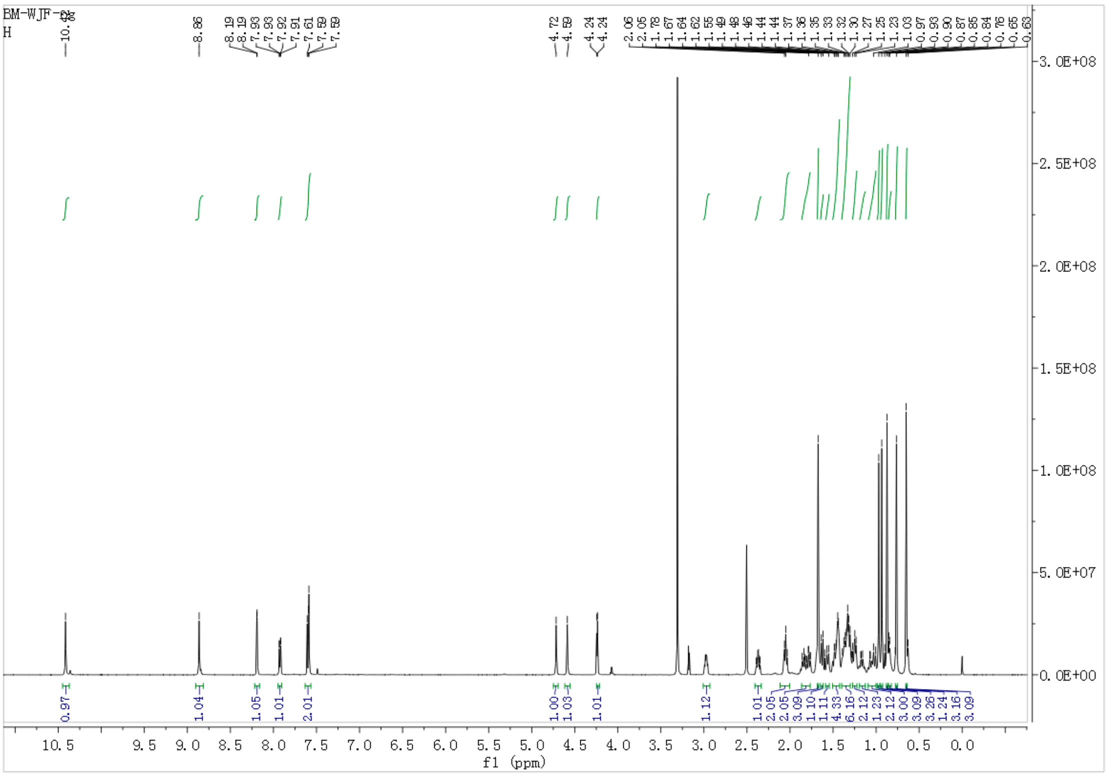
BM-WJF-og
H
f1 (ppm)

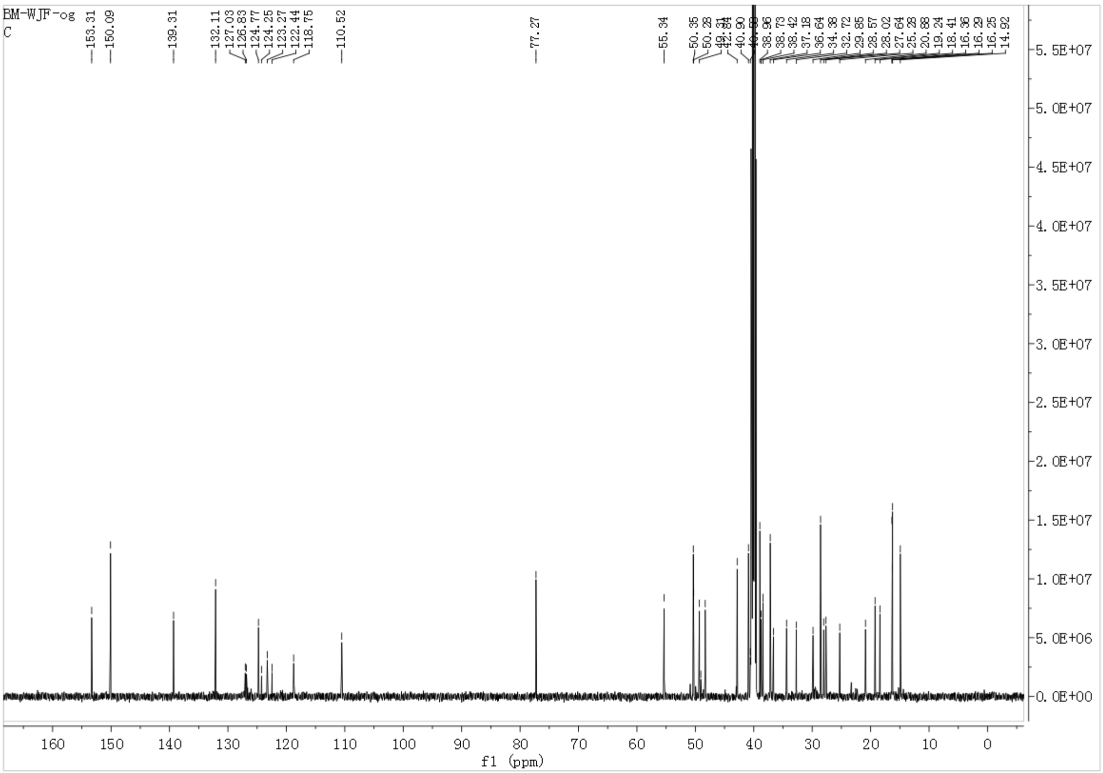
BM-WJF-og
C
f1 (ppm)

HRMS, ^{1}H NMR and ^{13}C NMR of compound 8a

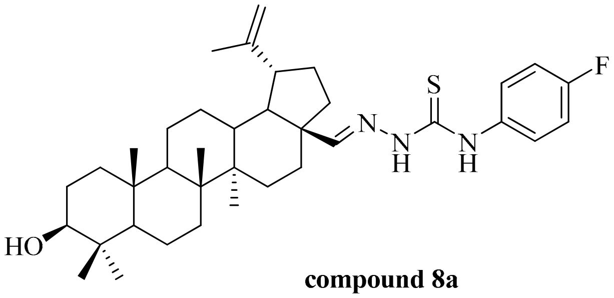

compound 8a

C37H54FN3OS

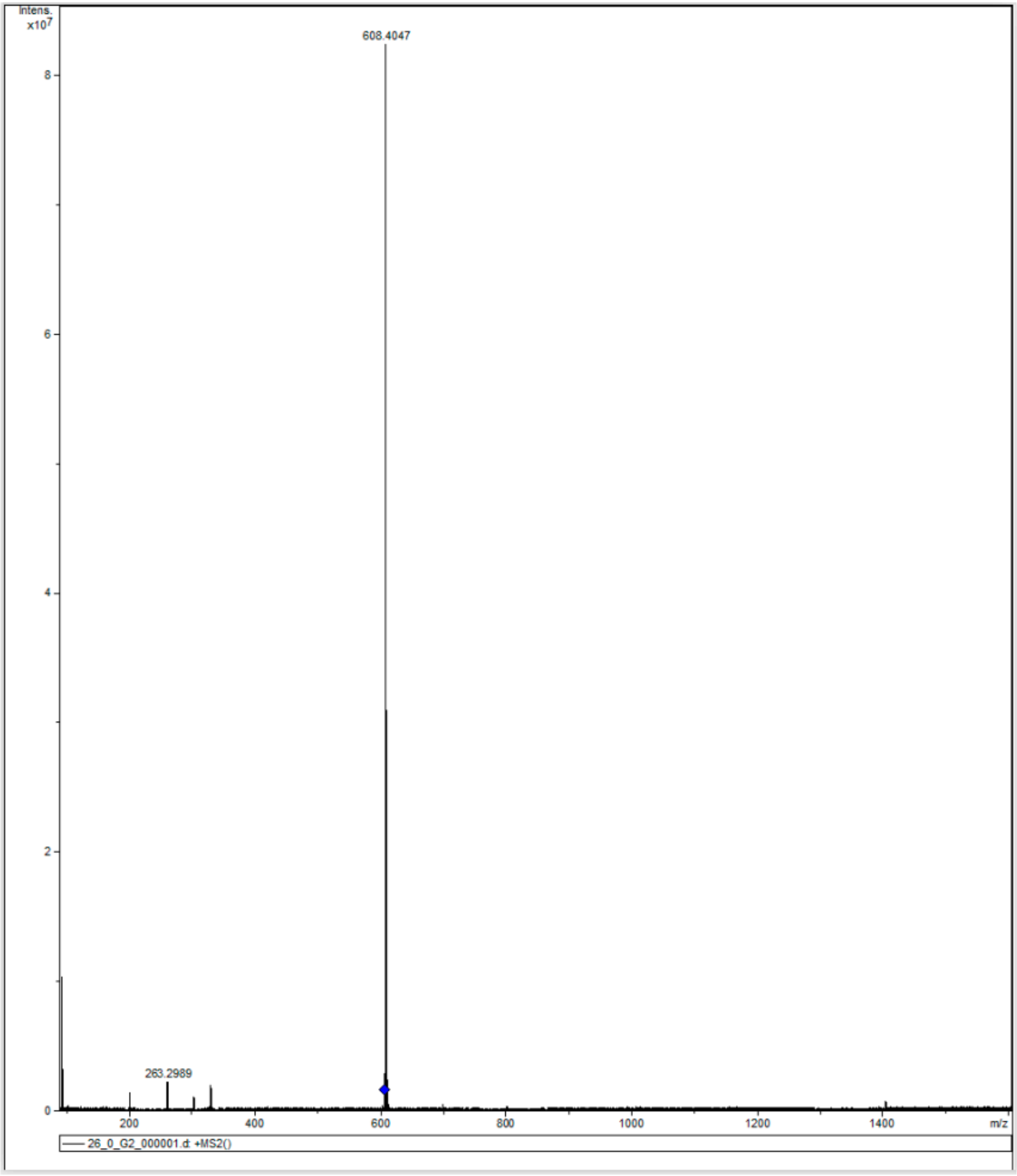

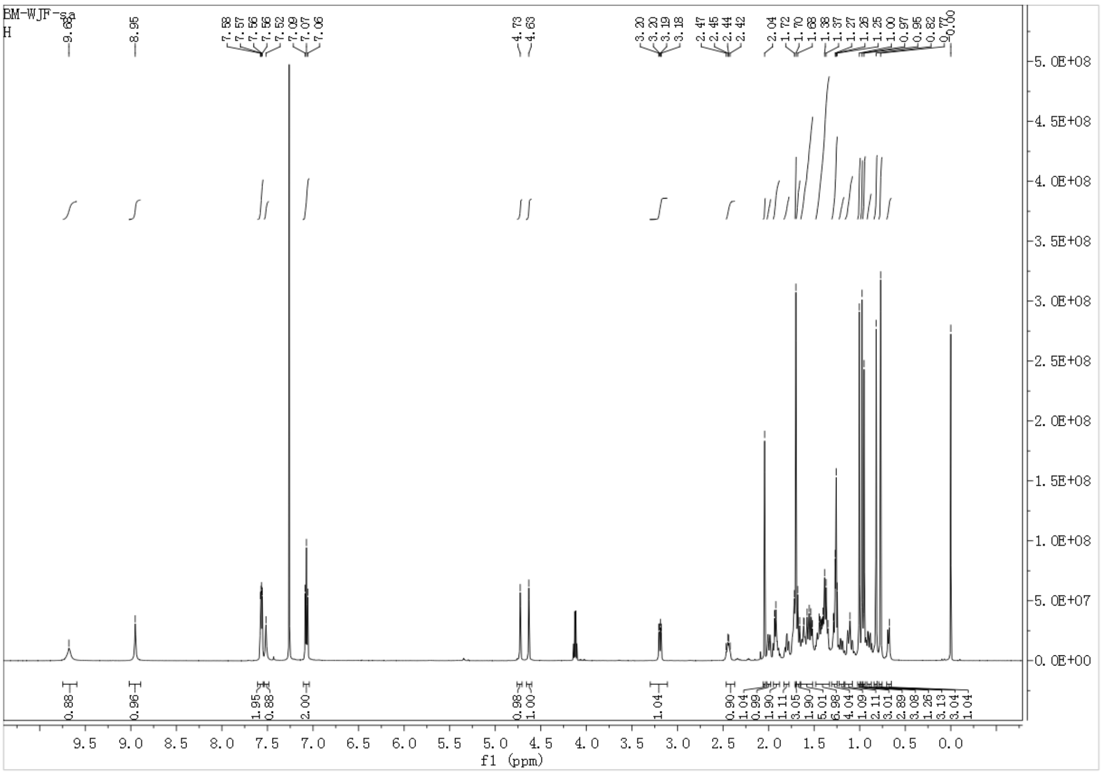
BM-WJF-sa
H
f1 (ppm)

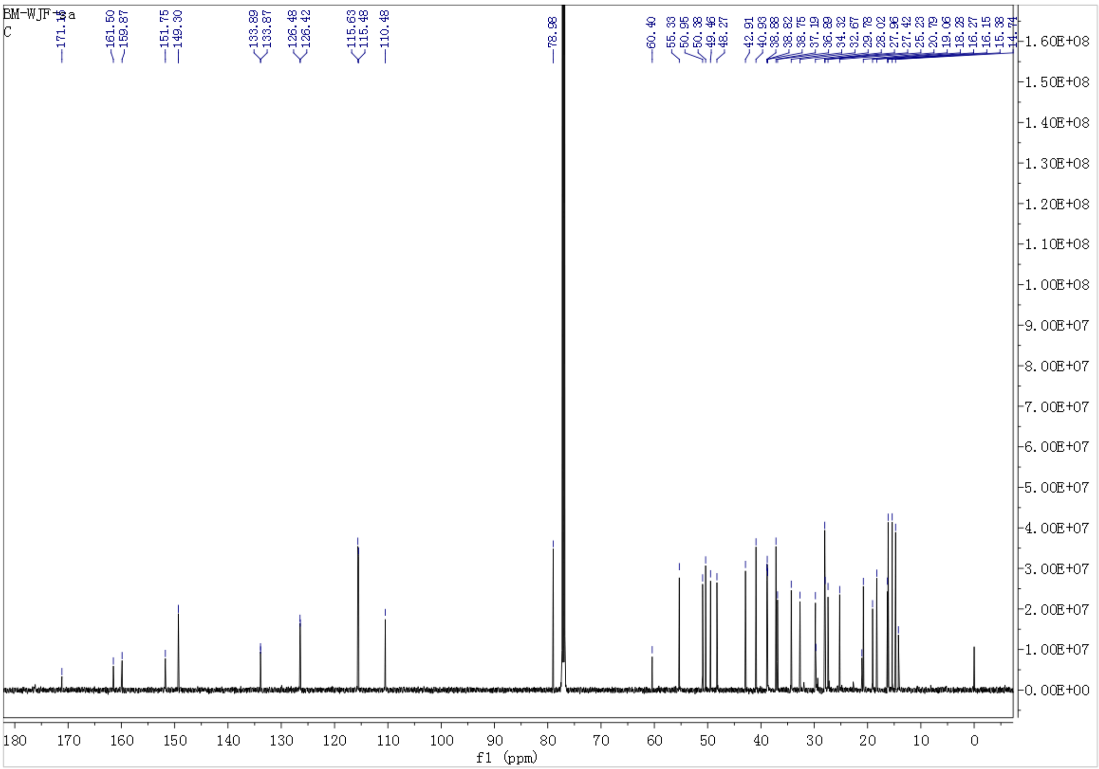
BM-WJF-sa
C
f1 (ppm)

HRMS, ^{1}H NMR and ^{13}C NMR of compound 8b

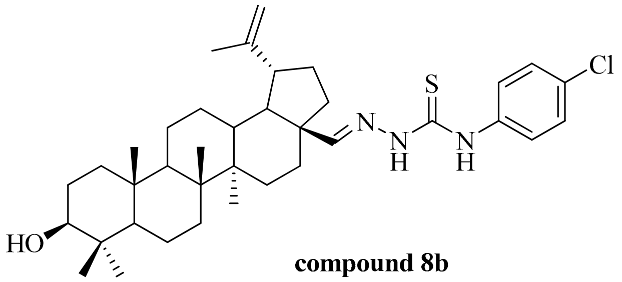

compound 8b

C37H54ClN3OS

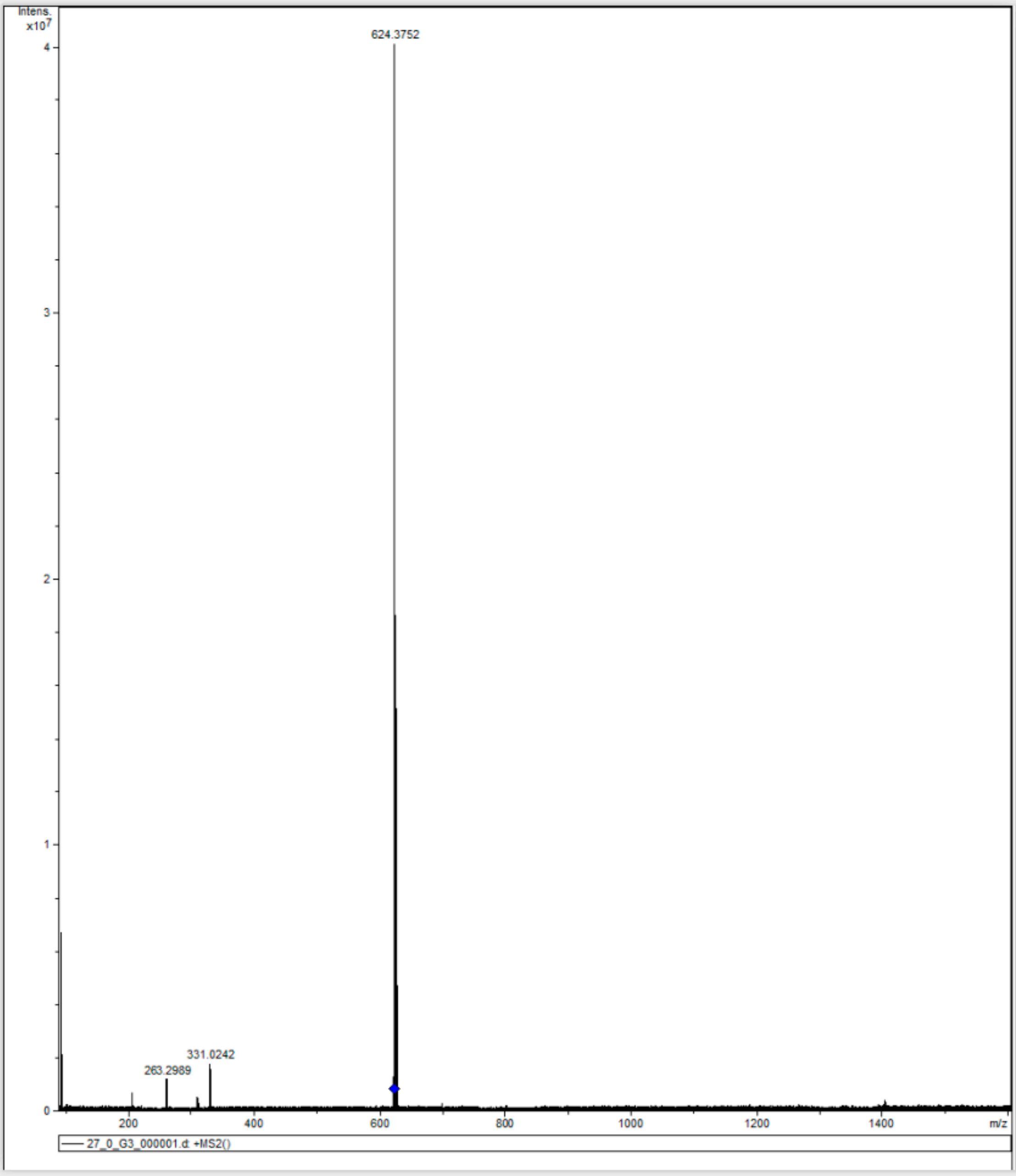

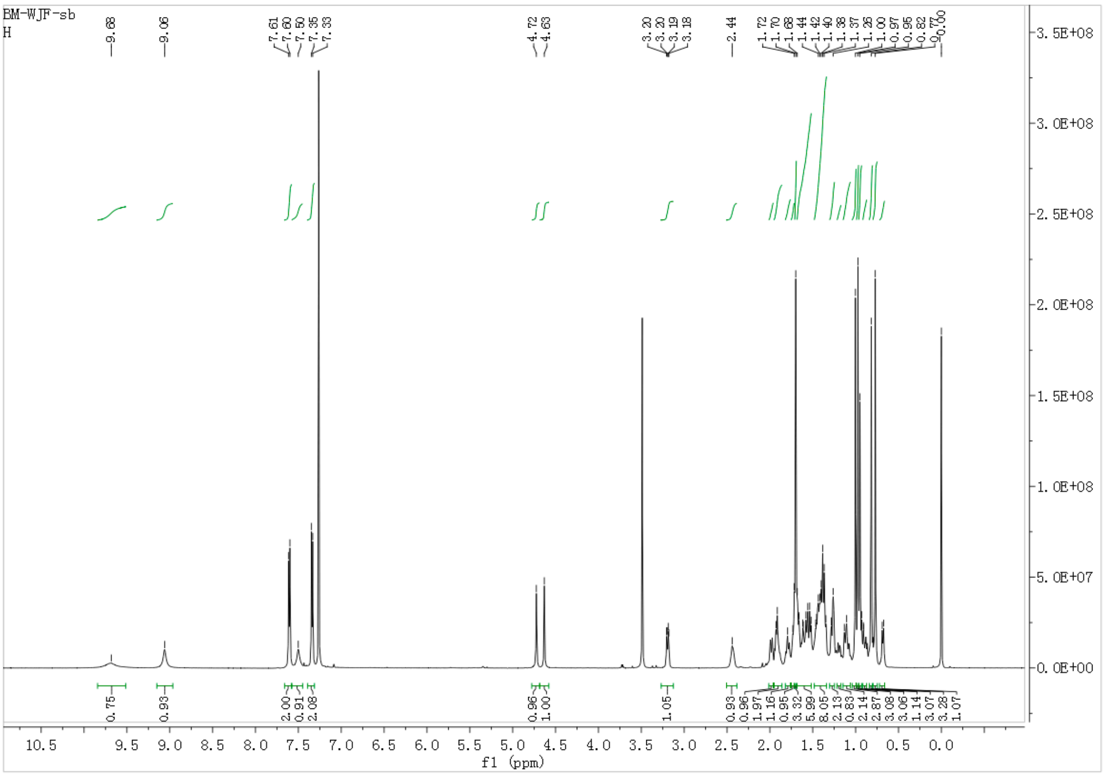

BM-WJF-sb
H
f1 (ppm)

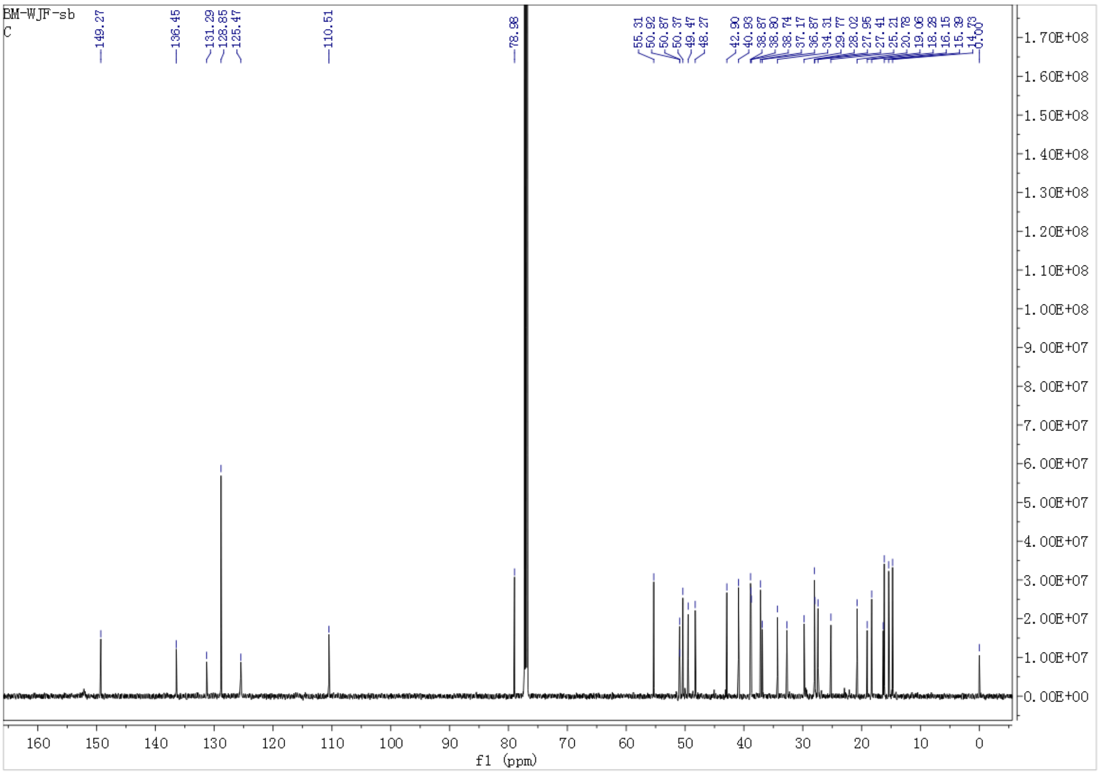

BM-WJF-sb
C
f1 (ppm)

HRMS, ^{1}H NMR and ^{13}C NMR of compound 8c

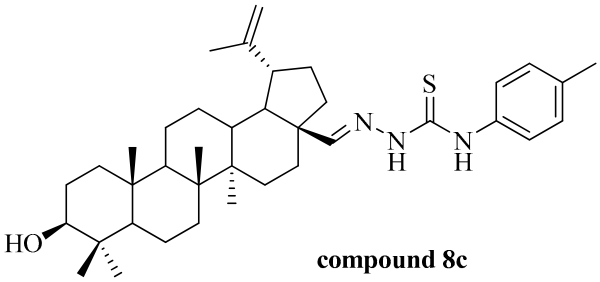

compound 8c

C38H57N3OS

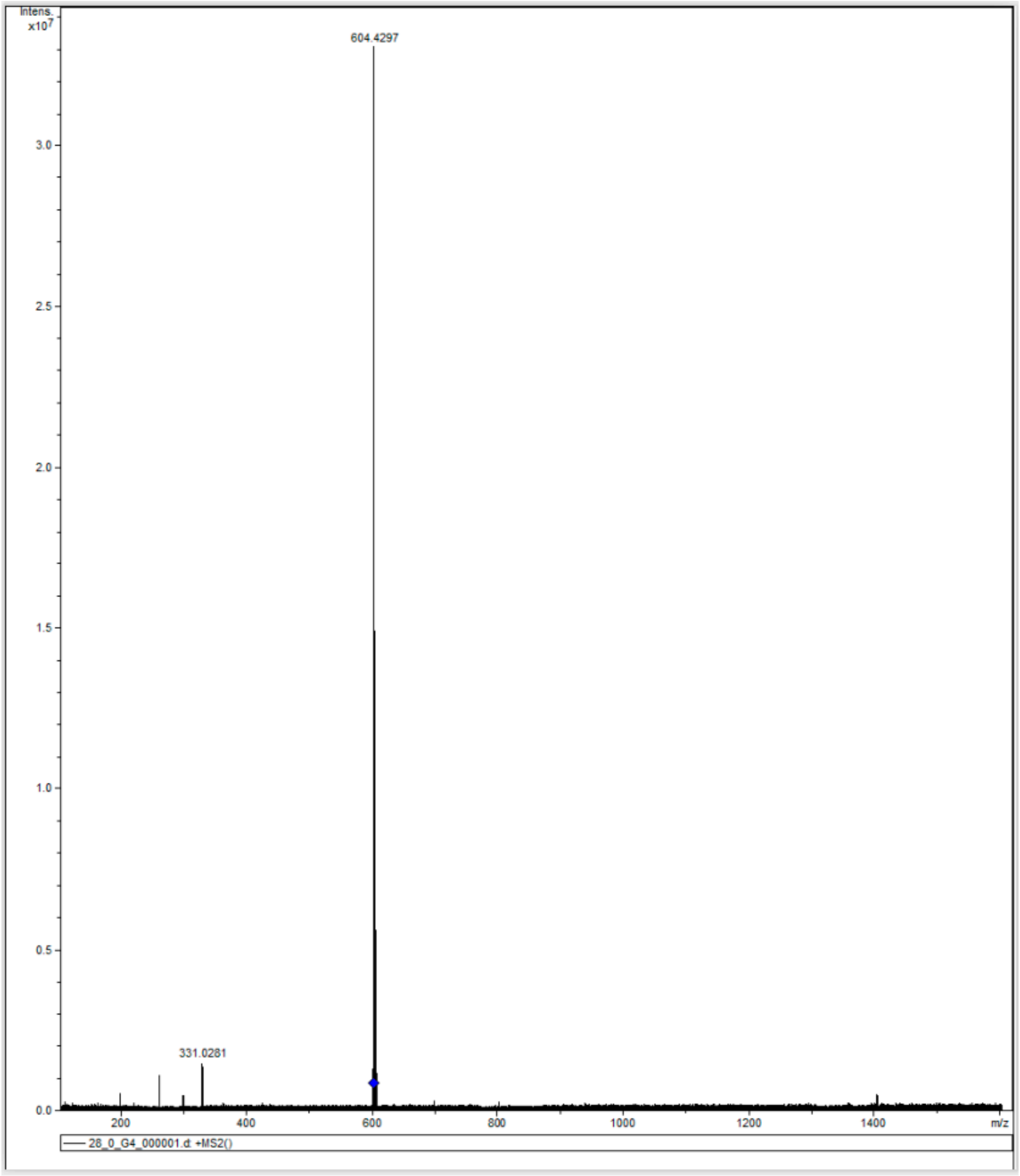

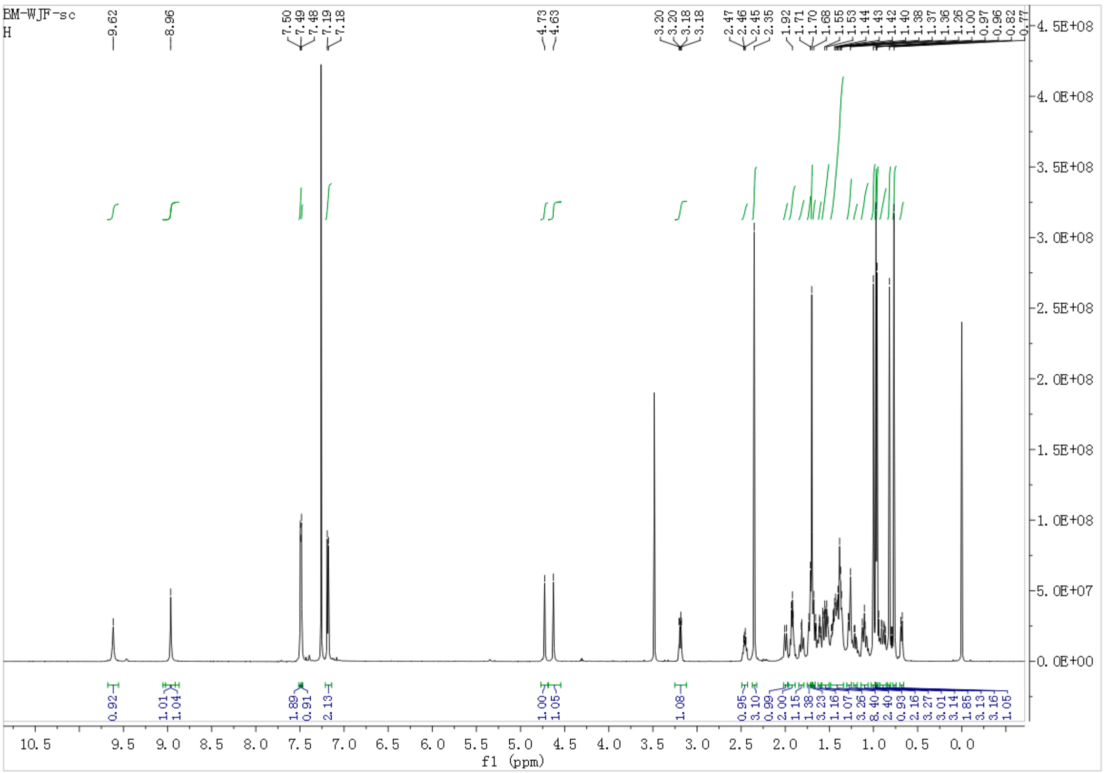

BM-WJF-sc
H
f1 (ppm)

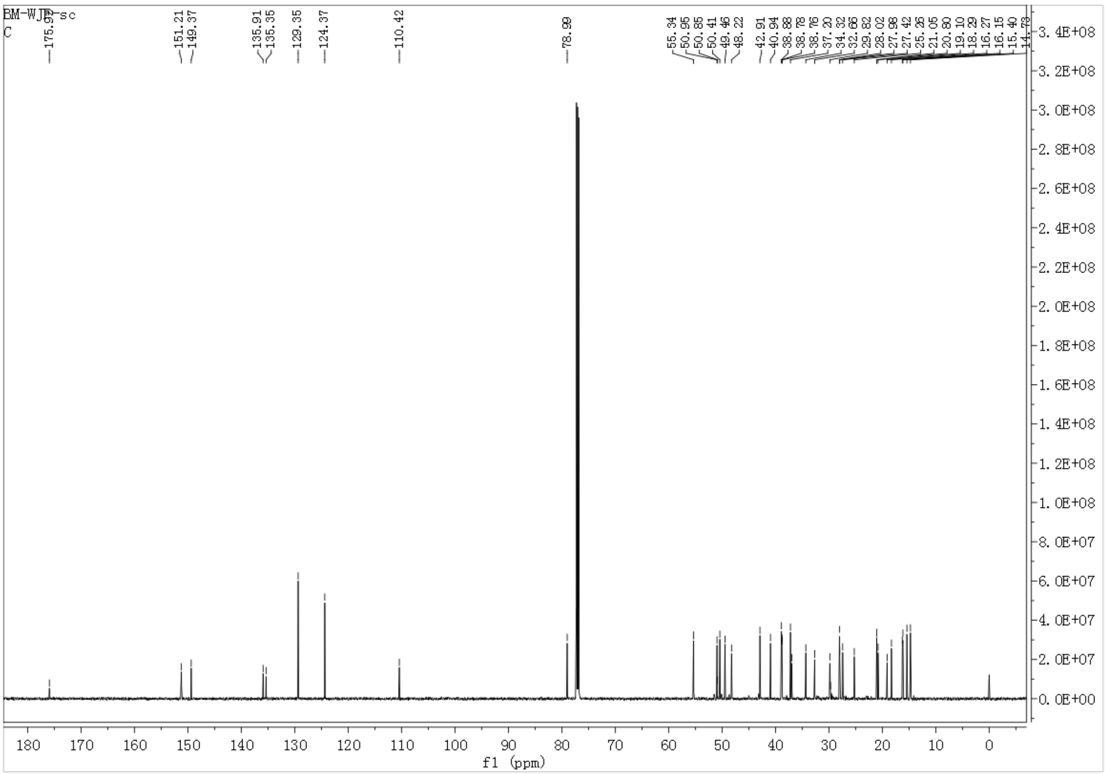

BM-WJF-sc
C
f1 (ppm)

HRMS, ^{1}H NMR and ^{13}C NMR of compound 8d

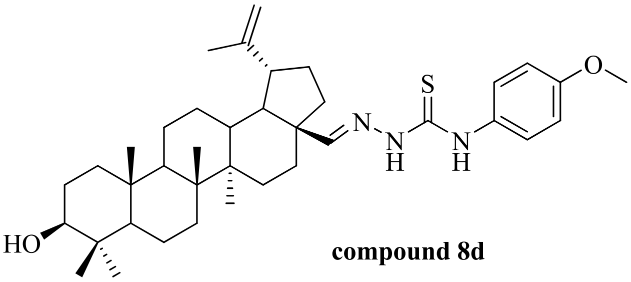

compound 8d

C38H57N3O2S

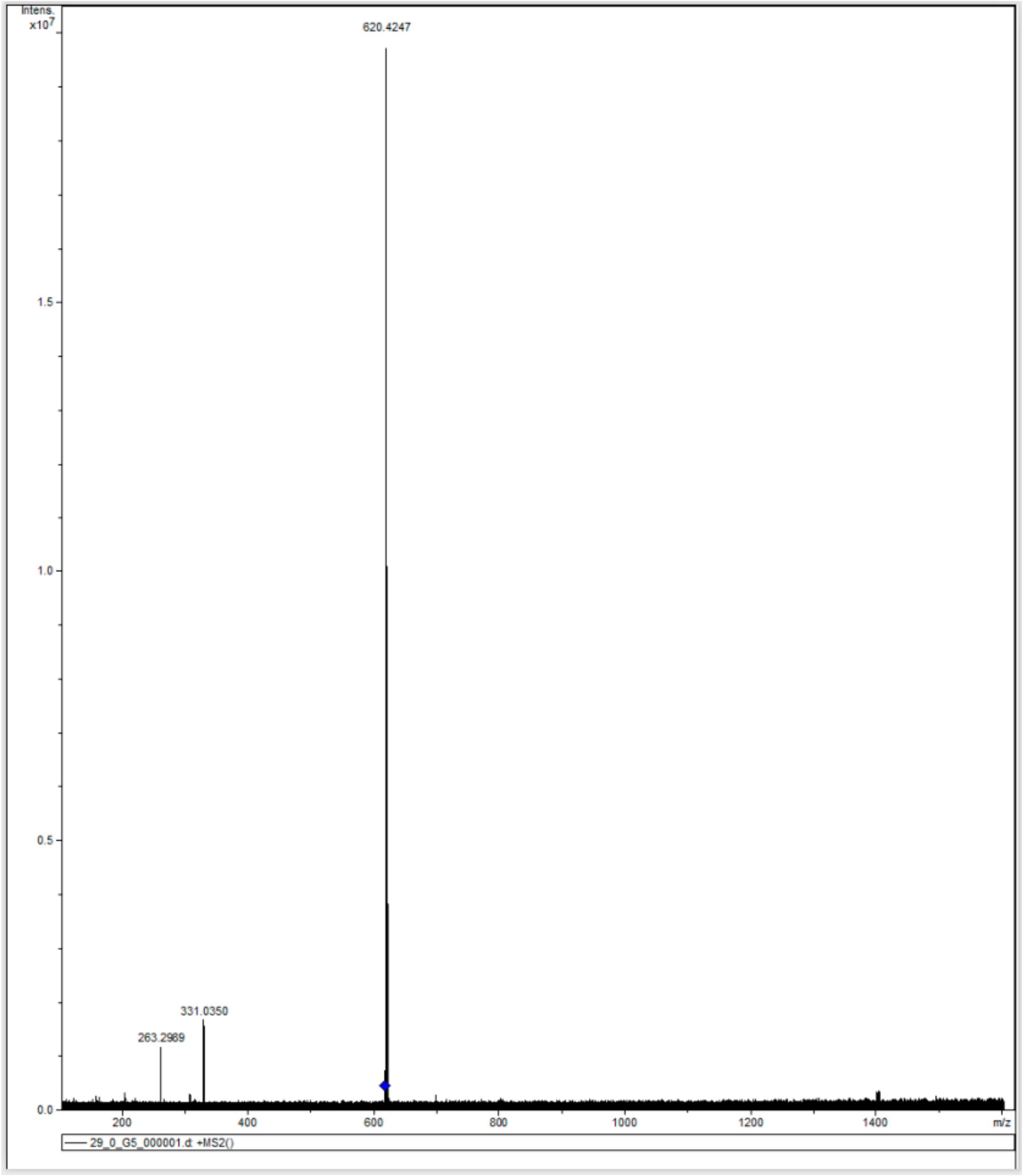

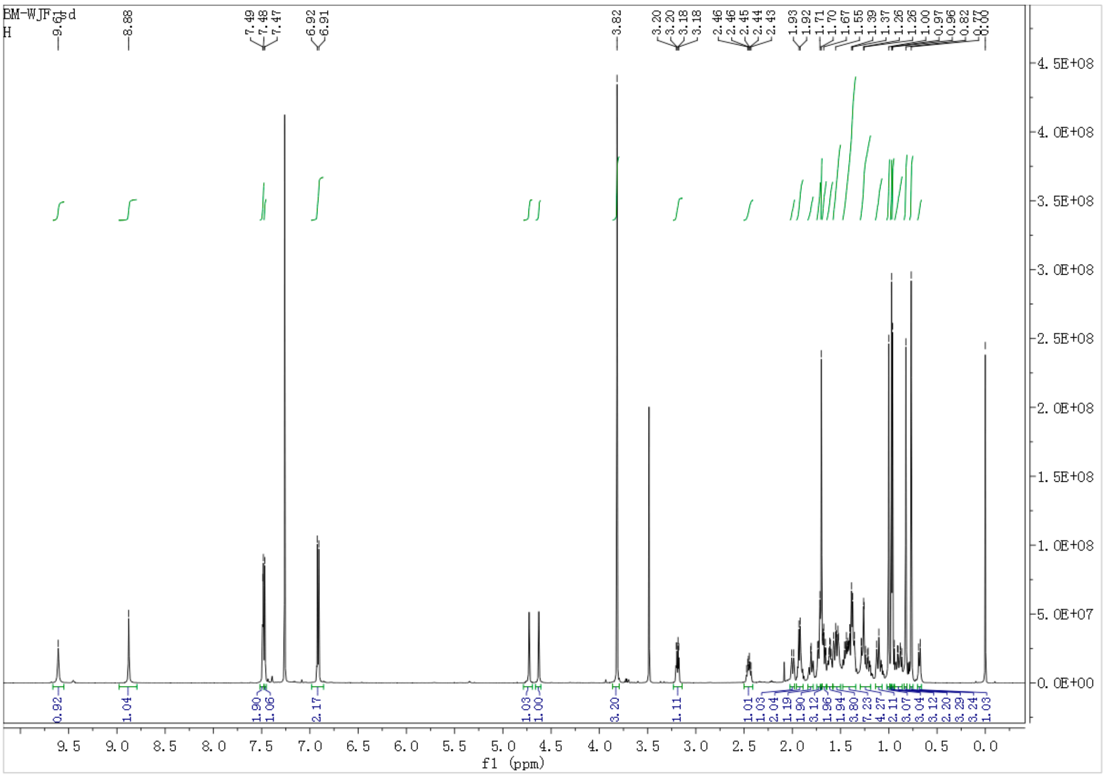
BM-WJF-Hd
H
f1 (ppm)

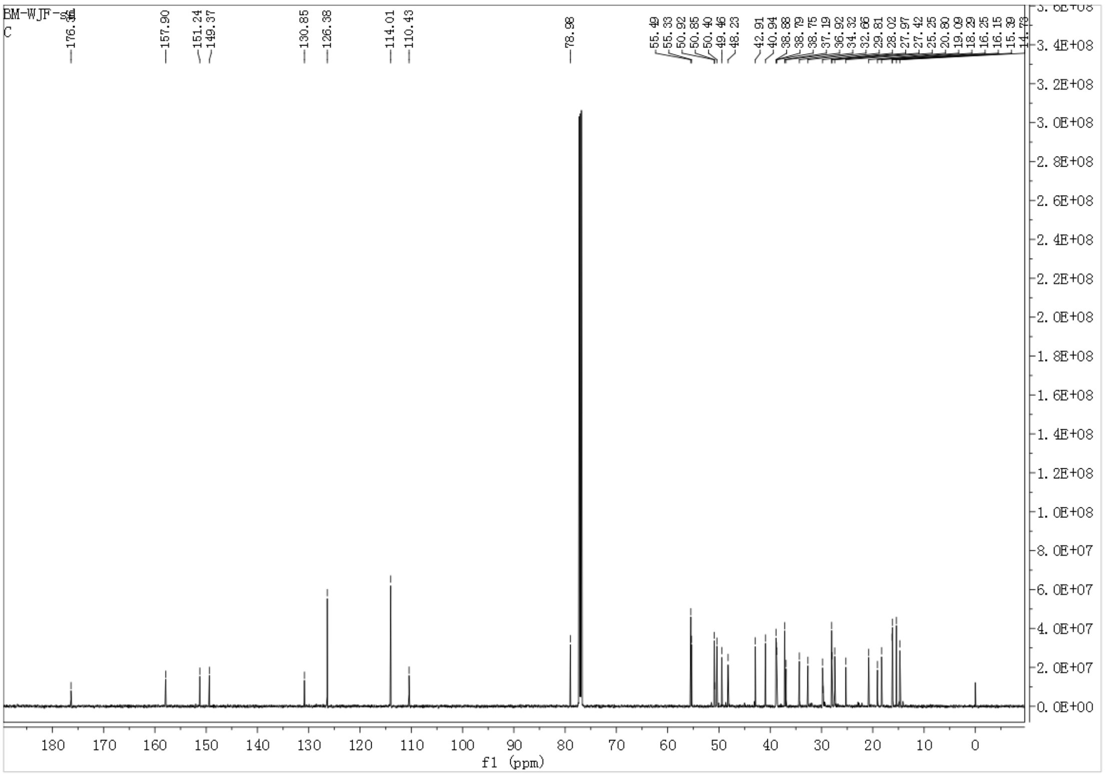
BM-WJF-sd
C
f1 (ppm)

HRMS, ^{1}H NMR and ^{13}C NMR of compound 8e

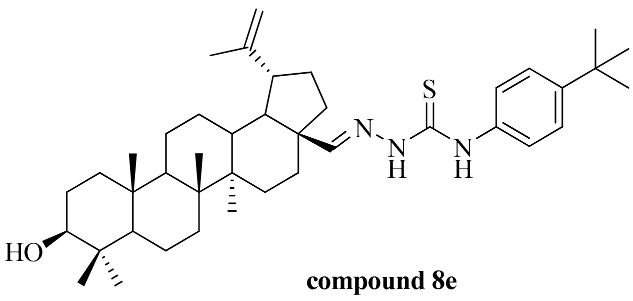

compound 8e

C41H63N3OS

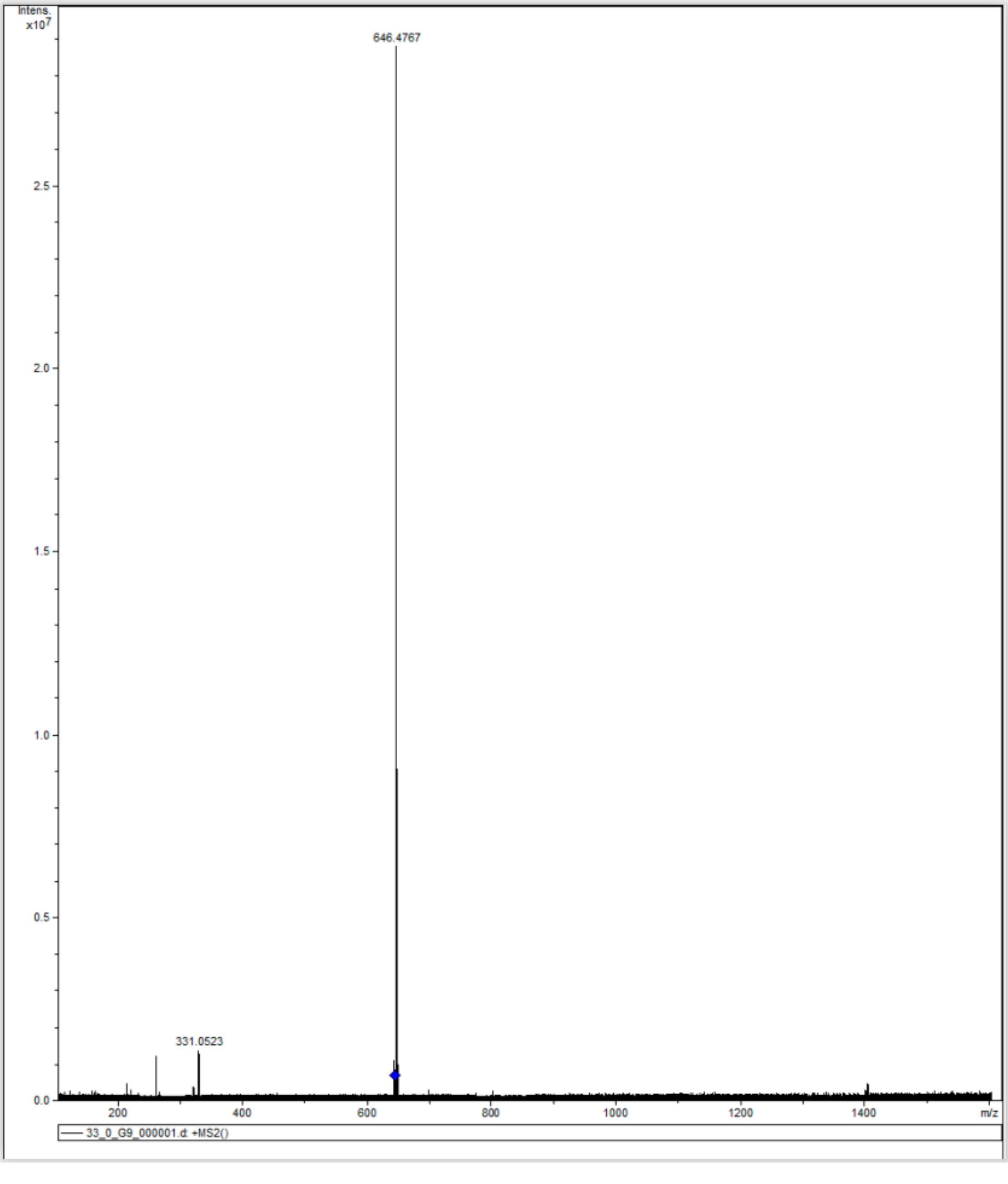

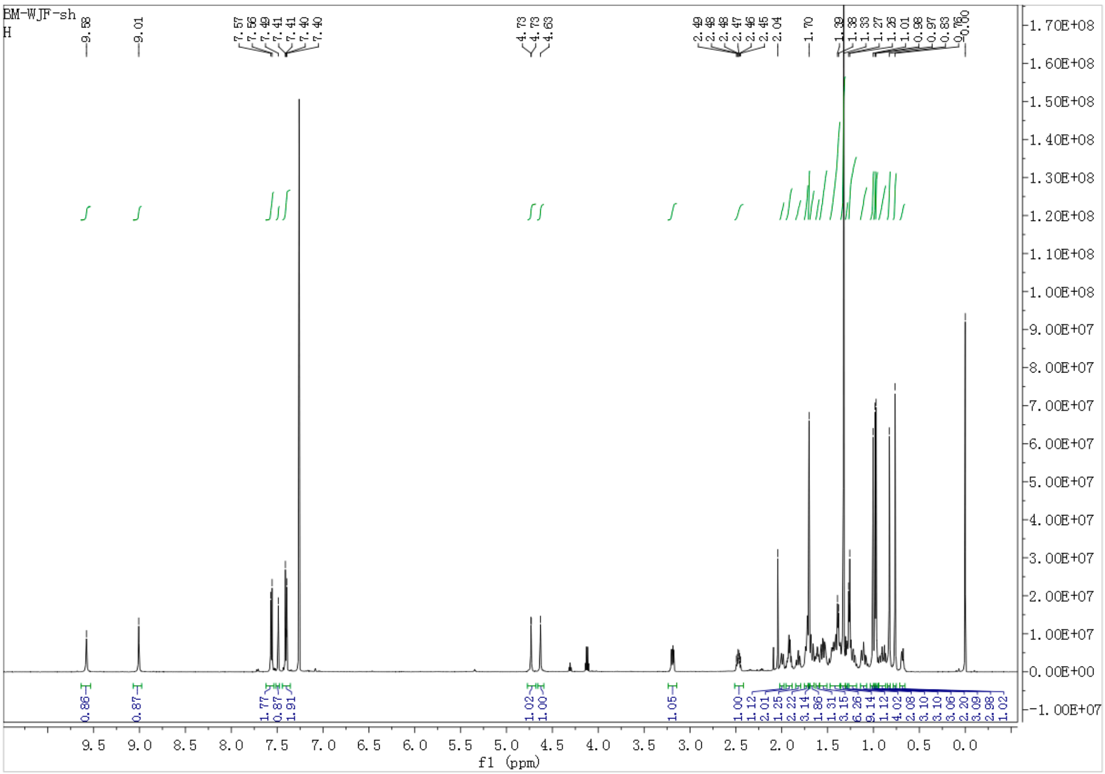

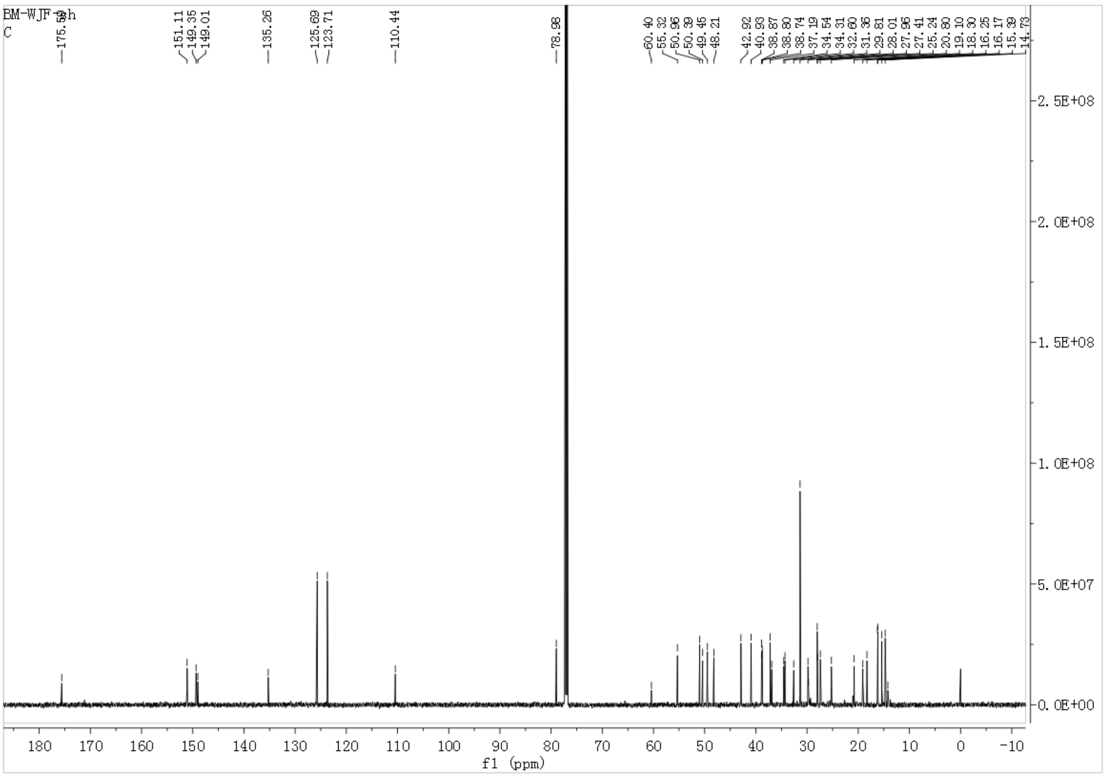

HRMS, ^{1}H NMR and ^{13}C NMR of compound 8f

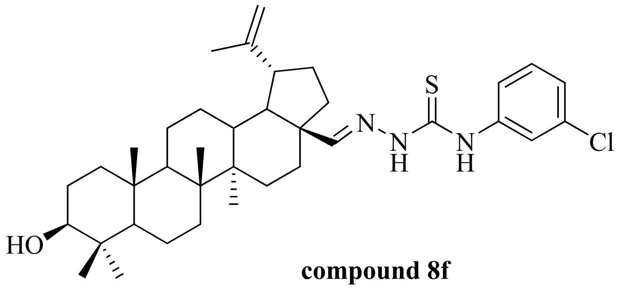

compound 8f

C37H54ClN3OS

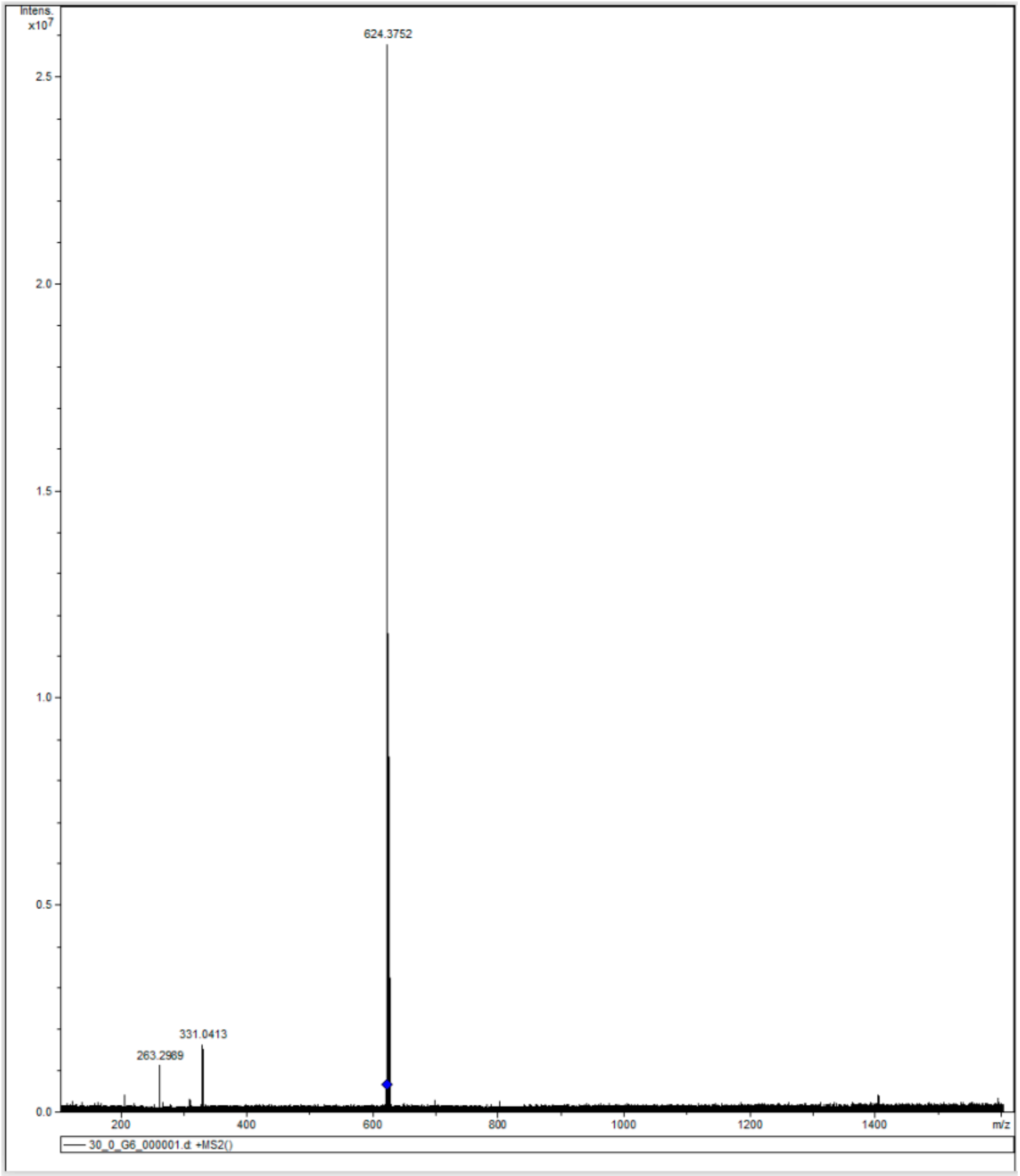

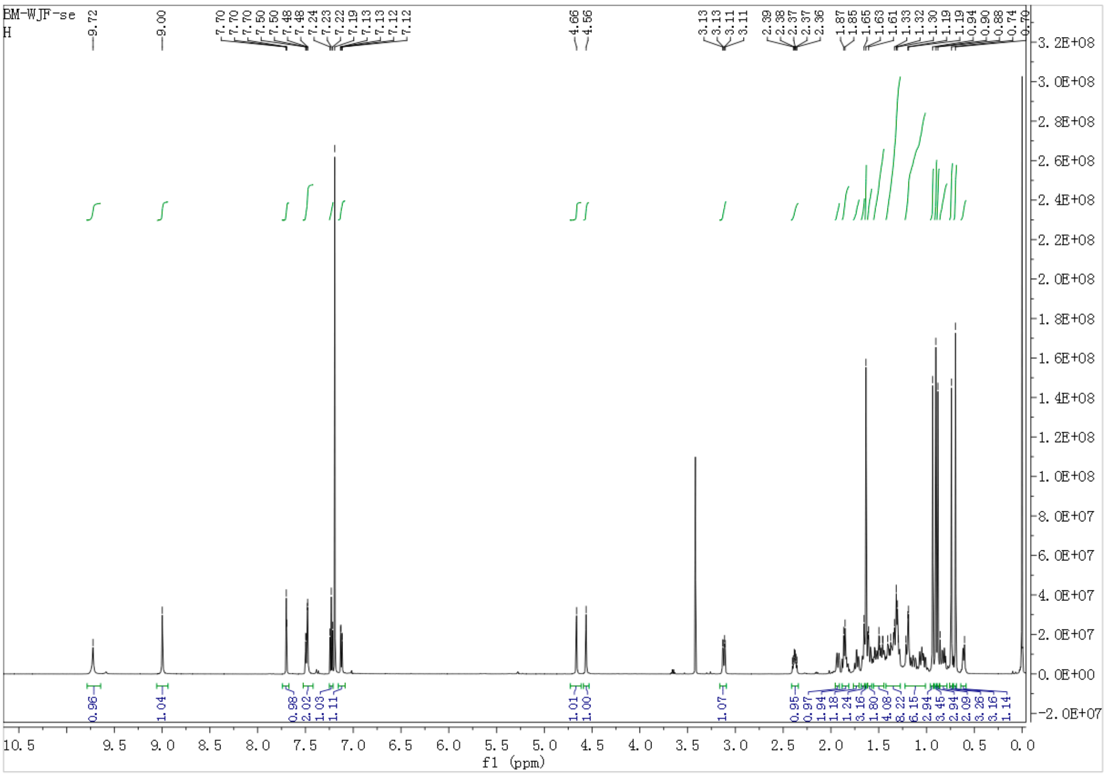
BM-WJF-se
H
f1 (ppm)

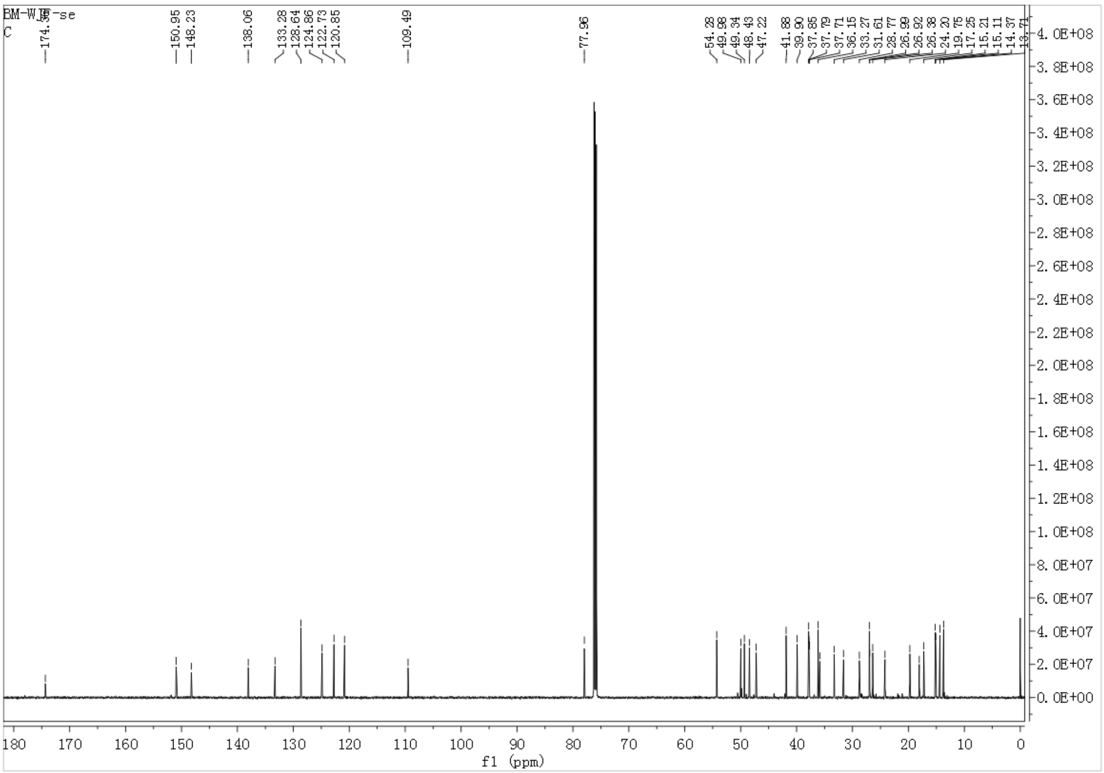
BM-WJF-se
C
f1 (ppm)

HRMS, ^{1}H NMR and ^{13}C NMR of compound 8g

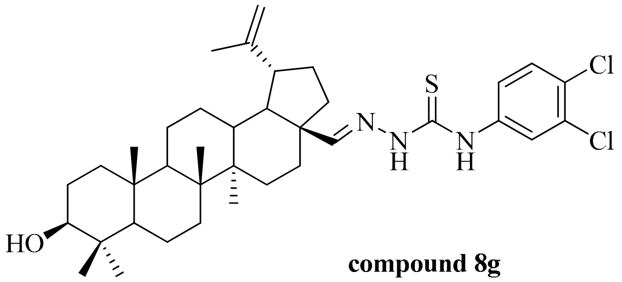

compound 8g

C37H53Cl2N3OS

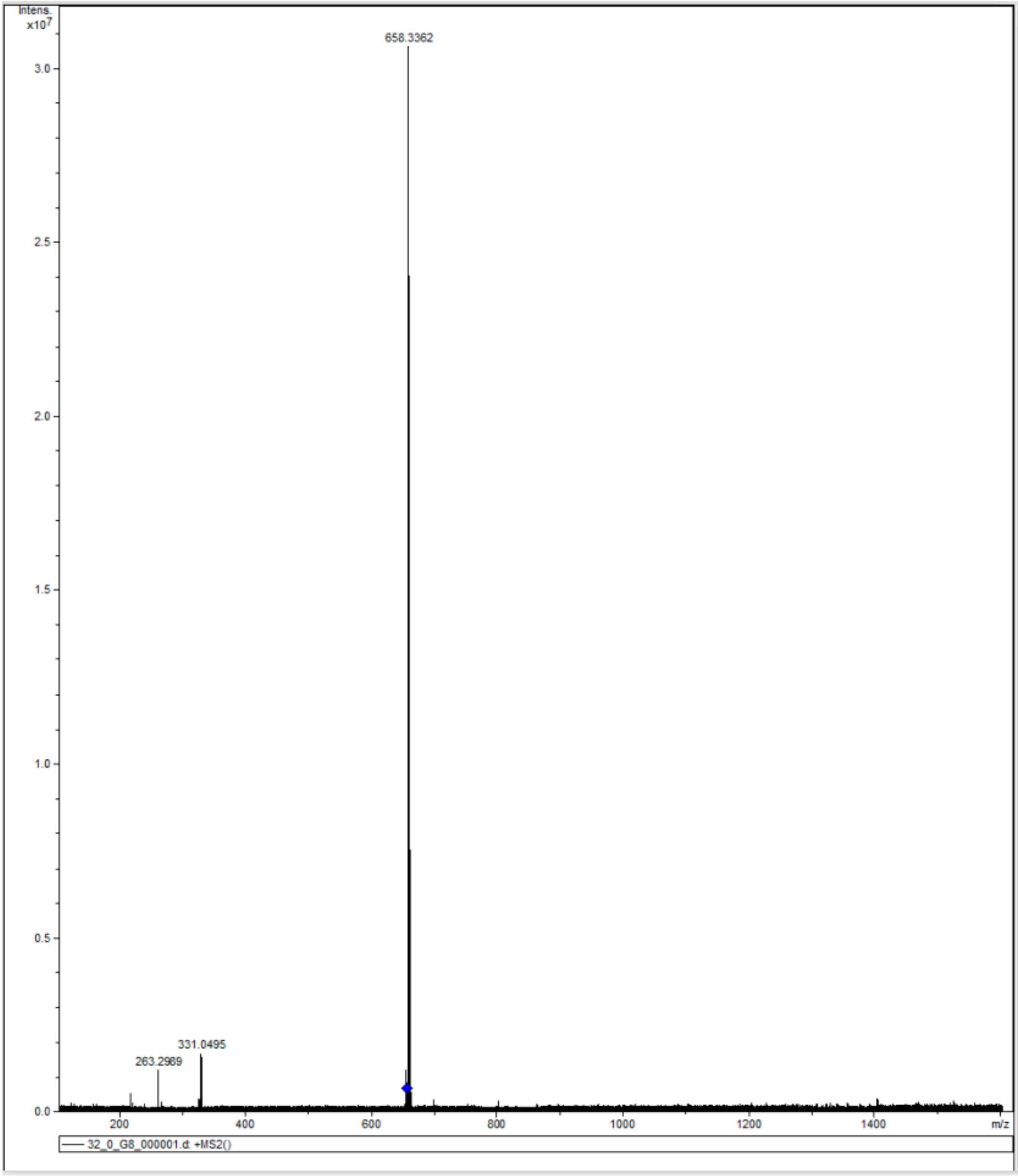

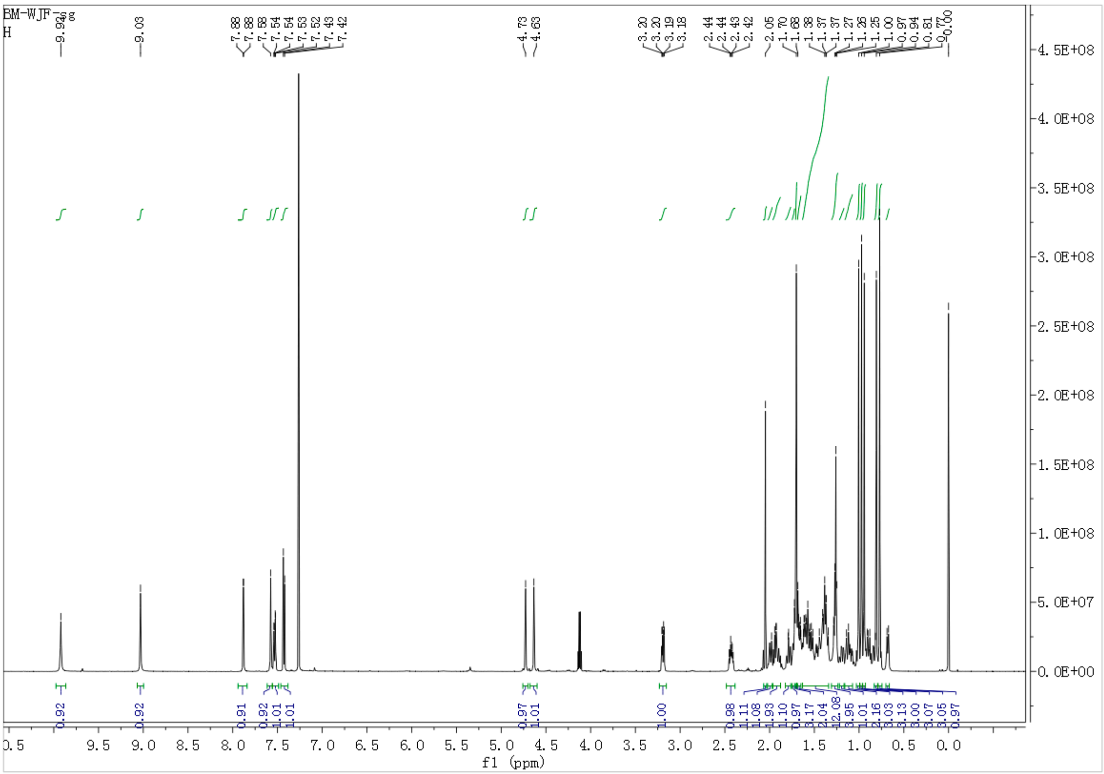
BM-WJF-sg
H
f1 (ppm)

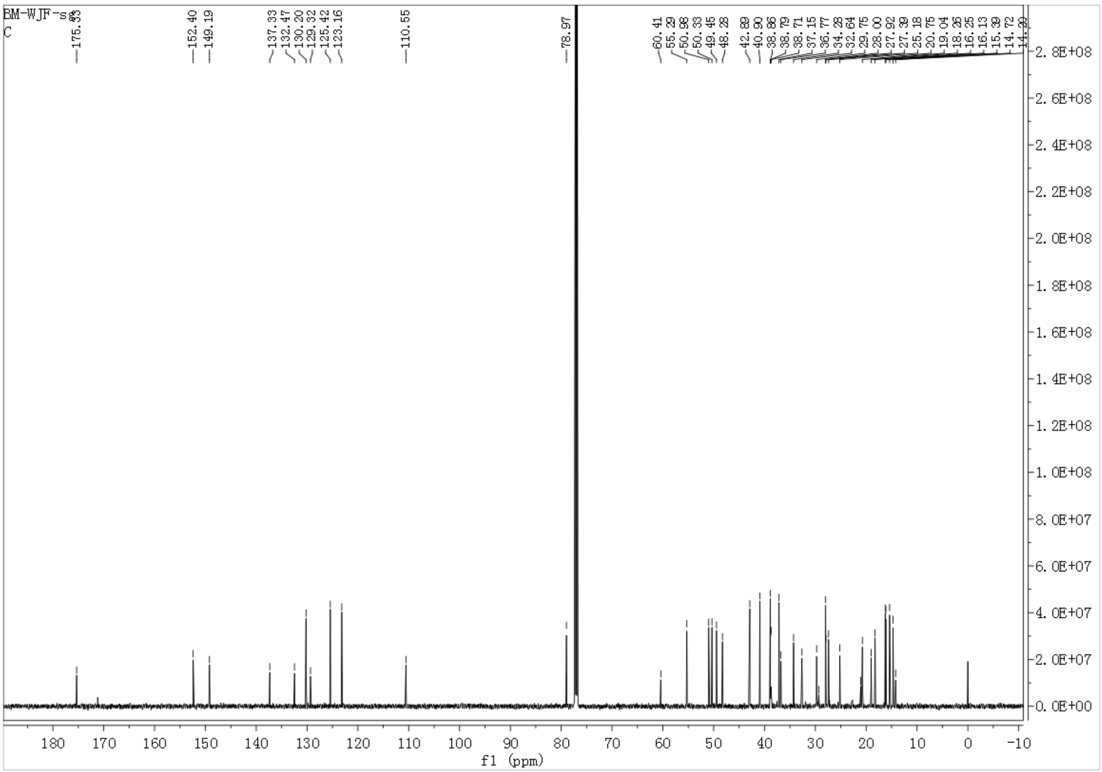
BM-WJF-sg
C
f1 (ppm)
